# Polarity protein MALS3 suppresses breast cancer stem cell-like properties by promoting TAZ inactivation and ubiquitination-dependent degradation

**DOI:** 10.1038/s41419-026-09010-7

**Published:** 2026-06-21

**Authors:** Yangyang Shang, Xinyi Gan, Bixia Tian, Yazhao Li, Ruiqi Wang, Ying Wang, Nanxin Liu, Huiwen Zhang, Jingyue Zhang, Shaoran Song, Zejian Yang, Yuchen Xie, Rumeng Pan, Juan Li, Bo Wang, Zhangjun Song, Yu Ren, Jie Liu, Peijun Liu

**Affiliations:** 1 Center for Translational Medicine, The First Affiliated Hospital of Xi’an Jiaotong University, Xi’an, China; 2 Key Laboratory for Tumor Precision Medicine of Shaanxi Province, The First Affiliated Hospital of Xi’an Jiaotong University, Xi’an, China; 3 Gansu Province Maternity and Child Health Hospital, Lanzhou, China; 4 Department of Radiation Oncology, The First Affiliated Hospital of Xi’an Jiaotong University, Xi’an, China; 5 Department of Radiation Oncology, Shaanxi Provincial People’s Hospital, Xi’an, China; 6 Department of Surgical Oncology, Shaanxi Provincial People’s Hospital, Xi’an, Shaanxi China; 7 Department of Breast Surgery, The First Affiliated Hospital of Xi’an Jiaotong University, Xi’an, China

**Keywords:** Cancer stem cells, Apicobasal polarity

## Abstract

Breast cancer stem cells are recognized as the key factors contributing to breast cancer progression and drug resistance, while polarity proteins play a pivotal role in regulating the fate of cancer stem cells and the progression of malignant tumors. However, the function of the polarity protein MALS3 in modulating cancer stem cell-like properties remains unclear. Here, we identify that the loss of the polarity protein MALS3 disrupts the homeostasis of mammary epithelial cells while increasing breast cancer stem cell-like properties. Mechanistically, MALS3 utilizes its L27 domain to mediate the interaction between the kinase LATS1 and TAZ, thereby promoting the phosphorylation of TAZ. Furthermore, MALS3 can recruit the E3 ubiquitin ligase TRIM29 to interact with TAZ. Subsequently, TRIM29 ultimately drives the degradation of TAZ via K48-linked polyubiquitination, and this regulatory process operates independently of the classical Hippo signaling pathway. We discover that the MALS3 upregulator mirtazapine suppresses stem cell-like properties and effectively enhances tumor sensitivity to doxorubicin in vivo. This study reveals the molecular mechanism by which the polarity protein MALS3 regulates breast cancer stem cell-like properties and suggests that MALS3 upregulation via mirtazapine may enhance tumor sensitivity to chemotherapeutic drugs.

## Introduction

The increased ability of stem cells to self-renew and the acquisition of malignant characteristics are crucial features of cancer [1, 2]. Cancer stem cells (CSCs) act as pivotal mediators in cancer development, exhibiting distinct specific markers across different malignancies. These include CD44⁺CD24⁻ and ALDH^hi^ for breast cancer, CD133⁺ for lung cancer and CD44⁺, Lgr5⁺ for colorectal cancer [3–5]. Unlimited self-regeneration capacity, multidirectional differentiation potential, anti-apoptotic properties, as well as drug resistance constitute the core characteristics through which CSCs regulate and sustain malignant tumor progression [2, 6, 7]. Currently, multiple chemotherapeutic agents have been identified as ineffective against cancer treatment, a phenomenon closely linked to CSC-like properties [8–11]. In treating breast cancer, commonly used chemotherapeutic drugs, including doxorubicin, paclitaxel, cisplatin, and gemcitabine frequently encounter issues of drug resistance and treatment insensitivity [12, 13]. Among these, doxorubicin is a chemotherapy drug that primarily works by inducing apoptosis in cancer cells. However, its inability to efficiently eliminate CSCs leads to breast cancer recurrence and insensitivity to chemotherapy drugs [14, 15]. On this basis, overcoming stem cell-like properties to enhance chemotherapy sensitivity remains a formidable challenge in breast cancer.

TAZ, as a pivotal molecule in the classic Hippo signaling pathway[16, 17], orchestrates multiple cellular processes and is abundantly expressed in various cancers, with its expression levels showing a positive correlation with CSC-associated markers expression [18–20]. Mechanistically, TAZ maintains the self-renewal capacity and multipotent differentiation capacity of CSCs by activating downstream stem cell transcription factors, thereby promoting tumorigenesis and development of drug resistance [19]. Current research on TAZ regulation focuses on phosphorylation via the kinase cascade within the classical Hippo pathway and subsequent ubiquitination-mediated degradation catalyzed by E3 ubiquitin ligase β-TRCP, exhibiting dynamic intracellular distribution, shuttling between the cytoplasm and nucleus [21, 22]. Moreover, some studies have indicated that regulatory pathways independent of the classical Hippo pathway are able to directly adjust TAZ stability or activity through non-kinase cascades [23, 24]. The involvement of other molecules in TAZ protein inactivation remains to be further explored.

Polarity proteins are key molecules regulating the establishment of cell polarity and normal cell division. In normal epithelial cells, apical polarity regulators (APRs), one type of polarity protein, participate in establishing the apical-basal axis through dynamic cooperation, thereby controlling epithelial morphogenesis and cell growth and survival [25]. When polarity proteins become dysregulated, cell division may become disordered, stem cells may acquire increased self-renewal capacity and malignant characteristics, leading to abnormal differentiation and the occurrence and malignant progression of tumors [1, 26, 27]. Disruption of polarity proteins occurs in numerous cancer types, including pancreatic, breast, bladder, and prostate cancers [28–31]. MALS3, as a crucial polarity protein, synergistically interacts with other critical polarity molecules within the CRB complex, serving as a central hub for epithelial cell morphogenesis, normal division, and functional homeostasis [32]. However, the impact of the polarity protein MALS3 in cancer remains largely unknown. A thorough grasp of the intrinsic mechanisms by which MALS3 acts in cancer progression is crucial, as this could provide novel insights for targeted therapies against malignant tumors.

In the current study, we establish that the depletion of polarity protein MALS3 impairs the preservation of acinar architecture in mammary epithelial cells and enhances breast cancer stem cell (BCSC)-like properties. MALS3 promotes the binding of kinase LATS1 and E3 ubiquitin ligase TRIM29 to TAZ through its L27 domain, thereby enhancing TAZ phosphorylation and K48-linked polyubiquitination, leading to TAZ protein inactivation and degradation. Additionally, we find that the MALS3 upregulator mirtazapine increases the therapeutic responsiveness of breast cancer cells toward the chemotherapeutic agent doxorubicin.

## Results

### The absence of MALS3 impairs the maintenance of acinar architecture in mammary epithelial cells MCF10A

**Fig. 1:**
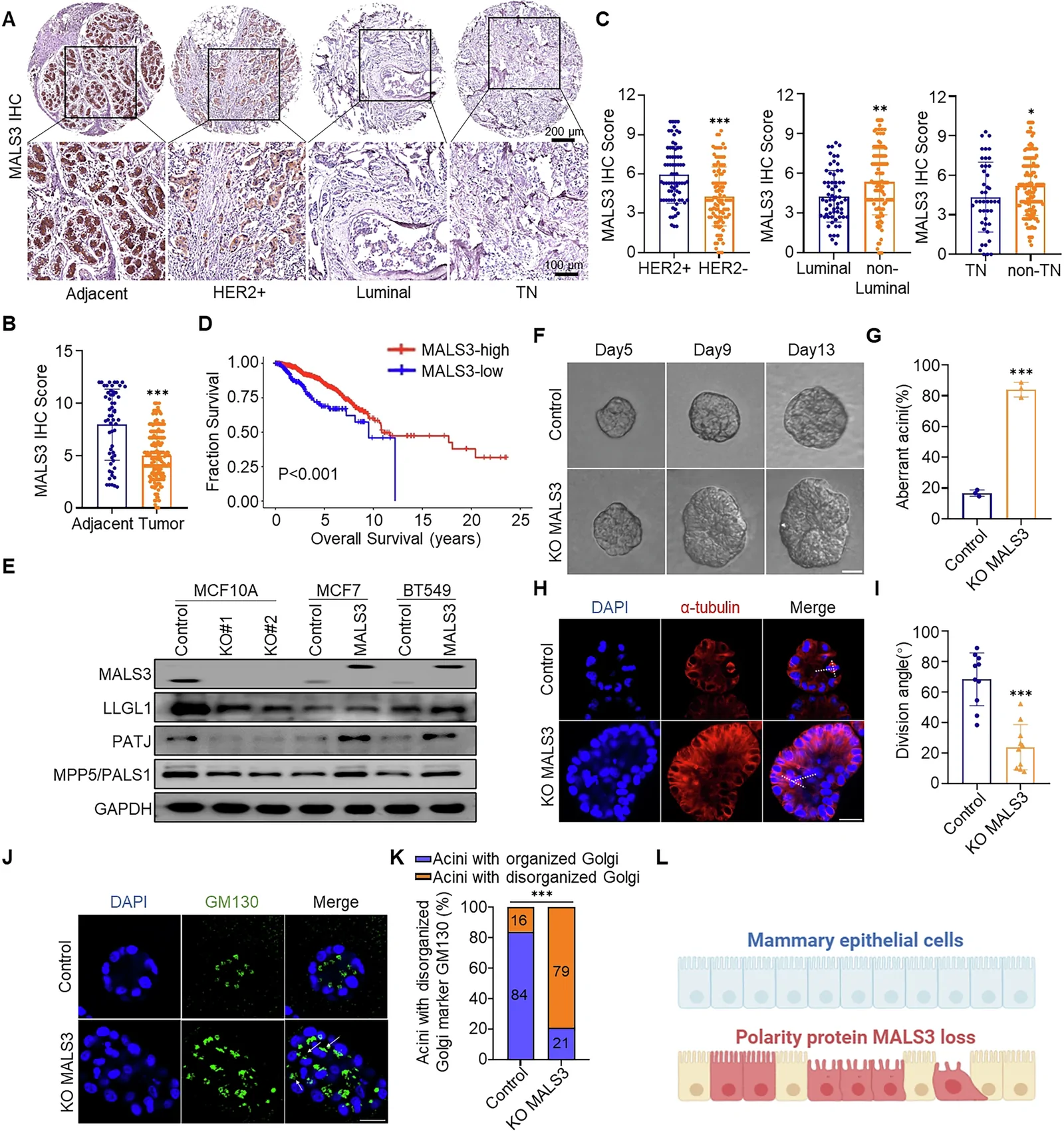
The absence of MALS3 impairs the maintenance of acinar architecture in mammary epithelial cells MCF10A. **A** Representative immunohistochemical images of breast cancer subtypes and adjacent breast tissue specimens. Scale bar: ×20 field of view = 200 μm; ×40 field of view = 100 μm. **B** Quantification of MALS3 immunohistochemical scores in 181 breast cancer specimens and 56 adjacent breast tissue specimens. **C** Quantification of MALS3 immunohistochemical scores in breast cancer specimens of different subtypes. **D** Stratified by MALS3 expression levels, survival analysis was conducted on breast carcinoma cases derived from TCGA breast cancer dataset. **E** Western blotting assays demonstrating other polarity proteins in MCF10A, MCF7, and BT549 cells with MALS3 knockout or overexpression. **F**, **G** Morphological changes and quantification of abnormal acinar formation rate in control versus MALS3-knockout MCF10A cells at day 13 of 3D culture. Scale bar: 50 μm. **H**, **I** Spindle axis alignment and quantitative assessment were performed in control versus MALS3-knockout 3D-cultured MCF10A cells at day 13 via immunofluorescence. Scale bar: 50 μm. **J** GM130 staining of control versus MALS3-knockout MCF10A cells cultured in 3D at day 13. Scale bar: 50 μm. **K** Quantification of Golgi disorganization based on GM130 staining. Data were obtained from three independent experiments with 100 samples per group. **L** Schematic diagram of polarity changes after MALS3 loss. Created with BioRender.com. Data are indicated as the mean ± SD of 3 independent replicates. ns not significant. *P < 0.05, **P < 0.01, ***P < 0.001. Statistical comparisons were conducted using by two-tailed unpaired t-test (**B**, **C**, **G**, **I**), log-rank test (**D**) or Fisher’s exact test (**K**).

Immunohistochemical images demonstrated that MALS3 expression was decreased in breast cancer tissues relative to adjacent breast tissues, particularly in Luminal and triple-negative (TN) subtypes (Fig. 1A–C). In 40 pairs of matched breast cancer tissue samples, MALS3 expression in tumor tissues was also lower than that in paired adjacent non-tumor tissues, especially in Luminal and TN subtypes (Fig. S1A, B). To investigate the potential link between MALS3 expression and prognosis in breast cancer patients, we grouped patients using an optimal cutoff value and analyzed clinical outcomes from the TCGA breast cancer dataset. Results revealed that high MALS3 expression was positively associated with increased overall survival (OS), and this correlation was particularly significant in HER2+, Luminal, and TN subtypes (Figs. 1D and S1C). In cell lines overexpressing or knocking out the polarity protein MALS3, we examined its effects on other polarity protein expression. Knocking out MALS3 reduced LGL1, PATJ, and MPP5/PALS1 expression, while overexpressing MALS3 promoted LLGL1, PATJ, and MPP5/PALS1 expression (Figs. 1E and S1D). To further investigate the role of MALS3 as a polarity protein in mammary epithelial cells, we performed three-dimensional morphogenesis experiments in both control and MALS3-knockout MCF10A cells. After 13 days of culture, compared to controls, MALS3-knockout cells formed enlarged, multi-lobular abnormal acini with no apparent apoptosis in luminal cells, and an increased number and volume of these abnormal acini structures (Figs. 1F, G and S1E). The α-tubulin staining revealed increased spindle misalignment in mitotic cells within the MALS3 knockout group (Fig. 1H, I). Notably, cell polarity exhibits a highly synergistic correlation with Golgi apparatus localization. Golgi marker GM130 was used to assess cell polarity, as the Golgi is apically positioned between epithelial nuclei and the apical lumen in polarized epithelial cells [33–35]. In three-dimensional morphogenesis assays, Golgi marker GM130 in control acini exhibited a regular distribution toward the acinar lumen. In contrast, in acini formed by MALS3-knockout cells, GM130 showed a disordered localization, and the proportion of acini with normal Golgi positioning was significantly reduced (Fig. 1J, K). These results indicate that loss of MALS3 directly disrupts the normal arrangement of the Golgi apparatus. E-cadherin staining, an epithelial polarity marker, revealed discontinuous E-cadherin distribution following MALS3 knockout (Fig. S1F). Therefore, MALS3 is essential for maintaining the polarity and normal structure of mammary epithelial cells (Fig. 1L). Our data indicate that the polarity protein MALS3 is downregulated in breast cancer tissues, and its absence impairs the maintenance of acinar architecture in the mammary epithelial cell line MCF10A.

### High expression of MALS3 suppresses breast cancer stem cell-like properties and increases sensitivity to chemotherapeutic drugs

**Fig. 2:**
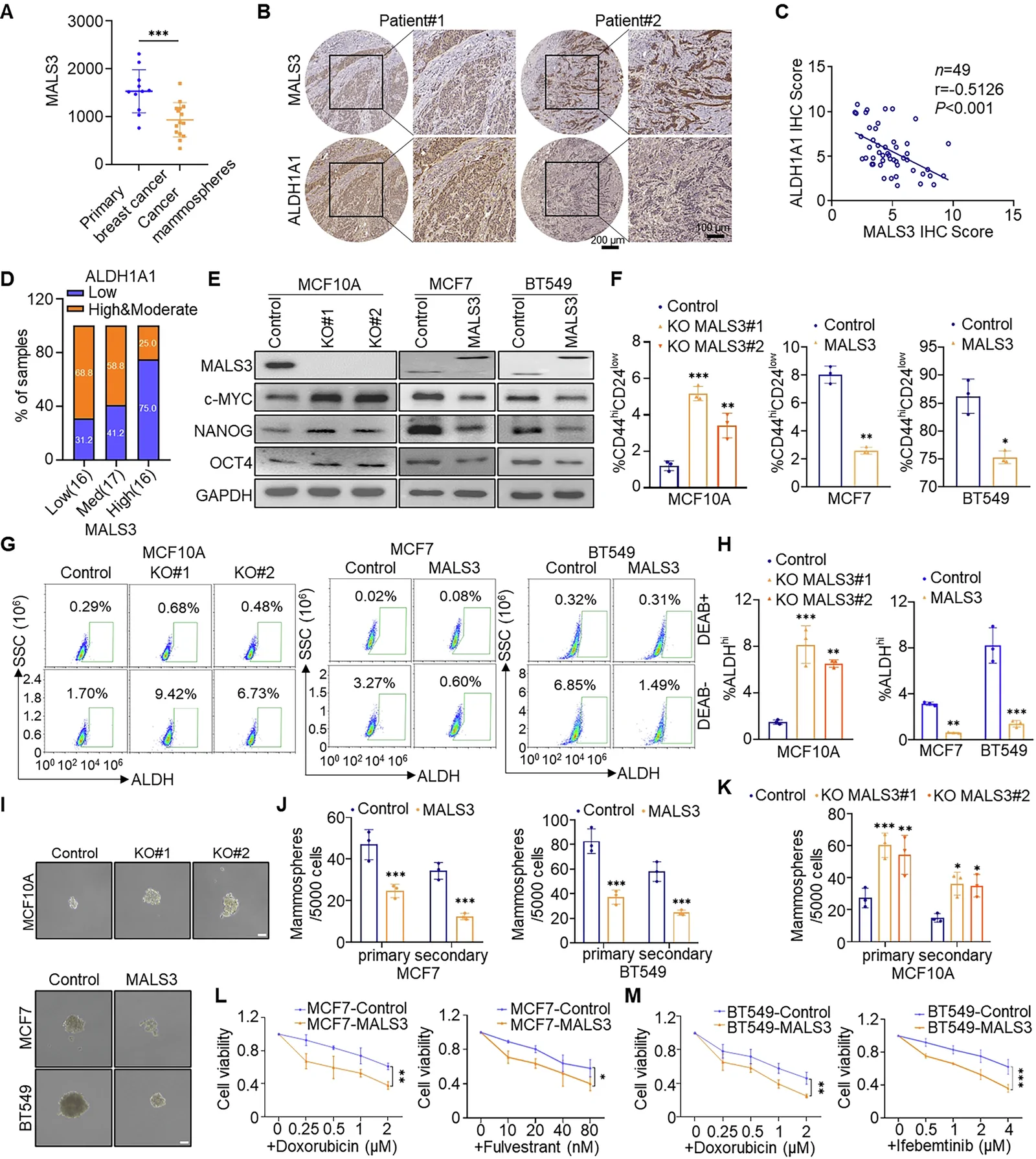
High expression of MALS3 suppresses breast cancer stem cell-like characteristics and increases sensitivity to chemotherapeutic drugs. **A** Analysis of MALS3 expression in primary tumors and mammospheres from the GSE7515 dataset. **B** Representative immunohistochemical images of MALS3 and ALDH1A1 in tissues from two breast cancer patients. **C** Correlation assessment of MALS3 and ALDH1A1 expression across 49 breast carcinoma specimens using immunohistochemical scoring. **D** Analysis of ALDH1A1 expression with varying MALS3 expression levels using immunohistochemical scoring. **E** Detection of CSC-associated c-MYC, NANOG, and OCT4 expression levels in MCF10A, MCF7, and BT549 cells with MALS3 knockout or overexpression via Western blotting. **F–H** Flow cytometry and quantification of the ratio of ALDH^hi^ and CD44^+^CD24^−^ subpopulations after knockout or overexpression. **I**–**K** Representative images and quantitative analysis of primary and secondary tumor spheroids in MCF10A, MCF7, and BT549 cells after MALS3 knockout or overexpression. Scale bar: 100 μm. MTT assays in control and MALS3-overexpressing cells following exposure to varying concentrations of doxorubicin and fulvestrant in MCF7 (**L**) and different concentrations of doxorubicin and ifebemtinib in BT549 (**M**). Data are indicated as the mean ± SD of 3 independent replicates. Abbreviation: *P < 0.05, **P < 0.01, ***P < 0.001. Statistical comparisons were conducted using by two-tailed unpaired t-test (**A**, **F**, **H**, **J**, **K**), Pearson’s r (**C**) or two-way ANOVA (**L**, **M**).

Whereas the structural homeostasis of mammary epithelial cells is closely linked to self-renewal and differentiation capacity of stem cells, and our existing findings confirmed that the loss of the polarity protein MALS3 impacts the maintenance of the MCF10A acinar structure in epithelial cells, we went on to explore how MALS3 influences stem cell-like characteristics. By analyzing the expression profiles from the GSE7515 gene set, which includes 11 primary breast cancer samples and 15 breast cancer mammospheres samples [36], we observed that MALS3 was notably reduced in breast cancer mammospheres (Fig. 2A). Through immunohistochemistry (IHC) assays, we evaluated MALS3 and ALDH1A1 expression in 49 breast carcinoma tissues and found an inverse correlation between MALS3 and ALDH1A1 expression levels (Fig. 2B, C). We categorized MALS3 expression into low, medium, and high groups based on immunohistochemical scoring. Among patients with low MALS3 expression, 68.8% exhibited moderate to high ALDH1A1 expression, while among those with high MALS3 expression, this proportion decreased to 25.0% (Fig. 2D). Furthermore, we examined c-MYC, OCT4, and NANOG, which are closely associated with CSCs, in MALS3 knockout and overexpression cells. Western blotting revealed that MALS3 knockout upregulated c-MYC, NANOG, and OCT4 expression, whereas MALS3 overexpression led to decreased protein levels (Fig. 2E). Flow cytometry assays demonstrated that MALS3 knockout within MCF10A cells increased the percentage of CD44^+^CD24^-^ and ALDH^hi^ subpopulations, whereas MALS3 overexpression reduced these subpopulations in MCF7 and BT549 cells (Figs. 2F–H and S2A, B). Cancer mammospheres assays demonstrated that when MALS3 was overexpressed, both number and volume of primary and secondary mammospheres were suppressed. In contrast, knocking out MALS3 boosted their formation (Fig. 2I–K). When MCF7 cells were exposed to doxorubicin or fulvestrant, and BT549 cells to doxorubicin or ifebemtinib, MALS3 overexpression increased cancer cell sensitivity to chemotherapy drugs compared to controls (Fig. 2L, M). These results demonstrate that MALS3 negatively regulates stem cell-like characteristics in breast cancer.

### High expression of MALS3 promotes inactivation and degradation of TAZ

**Fig. 3:**
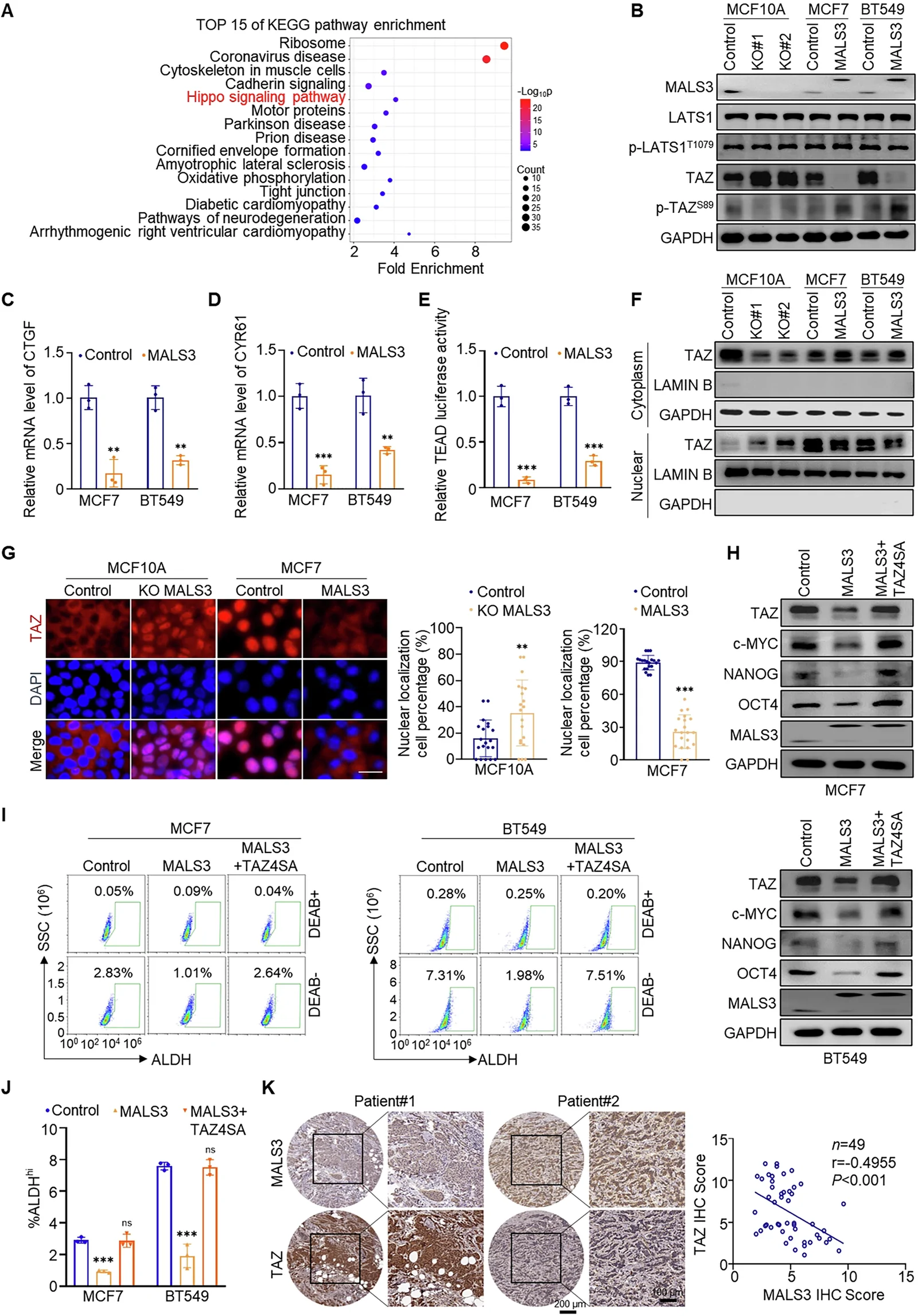
High expression of MALS3 promotes inactivation and degradation of TAZ. **A** KEGG pathways enriched with differentially expressed genes (DEGs) uncovered by IP-MS, comparing immunoprecipitation results between the IgG group and MALS3 group in MCF10A cells. **B** Western blotting detection of key Hippo pathway molecules following MALS3 knockout or overexpression. **C**, **D** mRNA levels of TAZ downstream CTGF and CYR61 following MALS3 overexpression, measured via RT-qPCR. **E** Luciferase reporter assay of TEAD following MALS3 overexpression. **F** Nuclear and cytoplasmic localization of TAZ assessed via Western blotting. Glyceraldehyde-3-phosphate dehydrogenase (GAPDH) served as an internal reference for cytoplasmic proteins. Lamin B served as an internal reference for nuclear proteins. **G** Immunofluorescence assay and quantification of TAZ subcellular localization, with at least 200 cells from 20 fields of view analyzed per condition. Scale bar: 25 μm. **H** Western blotting detection of c-MYC, OCT4, and NANOG expression in MCF7 and BT549 cells overexpressing MALS3 and transfected with TAZ4SA. **I**, **J** Flow cytometry detection and quantification of the ALDH^hi^ subpopulation proportion in MCF7 and BT549 cells overexpressing MALS3 and transfected with TAZ4SA. **K** Correlation of MALS3 and TAZ expression analyzed by immunohistochemical scoring in 49 breast cancer samples, along with representative immunohistochemical images in two patients. Scale bar: ×20 field of view = 200 μm; ×40 field of view = 100 μm. Data are indicated as the mean ± SD of 3 independent replicates. ns not significant. **P < 0.01, ***P < 0.001. Statistical comparisons were conducted using by two-tailed unpaired t-test (**C**, **D**, **E**, **G**, **J**) or Spearman’s *r* (**K**).

To further investigate the potential mechanisms by which MALS3 suppresses BCSC-like properties, we performed mass spectrometry (MS)-based proteomics analysis. The proteomics analysis represents all proteins bound to MALS3 (IP-MS) in MCF10A cells. The results revealed a potential association between MALS3 and the Hippo pathway (Fig. 3A). Subsequently, we investigated the protein expression of key molecules in Hippo pathway. The levels of LATS1 and phosphorylated LATS1 showed no significant changes in MALS3-knockout MCF10A cells. However, the levels of YAP and TAZ were elevated, accompanied by reduced phosphorylation of YAP at Ser127 and TAZ at Ser89. Consistently, elevated MALS3 expression reduced YAP/TAZ protein levels while increasing YAP^S127^ and TAZ^S89^ protein levels in MCF7 and BT549 cells (Figs. 3B and S3A). In MALS3 knockout cells, mRNA levels of the YAP/TAZ downstream CTGF and CYR61 increased, whereas MALS3 overexpression decreased CTGF and CYR61 mRNA levels (Figs. 3C and D and S3B). Subsequently, we assessed the activity of TEAD, a transcription factor downstream of TAZ, using an 8×GTIIC luciferase reporter assay. Our results showed that MALS3 knockout increased TEAD activity, whereas MALS3 overexpression decreased TEAD activity (Figs. 3E and S3C). Cytoplasmic and nuclear protein fractionation and immunofluorescence (IF) assays further confirmed that YAP/TAZ was more extensively transported into the nucleus in MALS3-knockout MCF10A cells, whereas MALS3 overexpression inhibited YAP/TAZ nuclear translocation (Figs. 3F, G and S3D–F).

As demonstrated by previous studies, the transcription coactivator TAZ exerts a more prominent function in modulating BCSC-like properties [18, 37]. Therefore, we will further explore the relationship between MALSS3 and TAZ. TAZ4SA, as a mutant carrier of TAZ, is constructed by site-directed mutagenesis targeting four key serine phosphorylation sites of the TAZ protein to generate a constitutively activated form of TAZ [38, 39]. Following treatment with TAZ4SA in MCF7 and BT549 cells, the downregulation of c-MYC, NANOG, and OCT4 induced by MALS3 overexpression was restored, and the reduction in the ALDH^hi^ subpopulation proportion caused by MALS3 overexpression was reversed (Fig. 3H–J). To investigate how MALS3 influences TAZ protein abundance in clinical samples, we assessed MALS3 and TAZ expression within breast cancer specimens (*n* = 49) using IHC. An inverse correlation was observed between MALS3 and TAZ expression (Figs. 3K and S3G). Collectively, these findings provide evidence that high MALS3 expression inhibits TAZ nuclear translocation and promotes degradation of TAZ.

### MALS3 suppresses stem cell-like characteristics of breast cancer in vivo

**Fig. 4:**
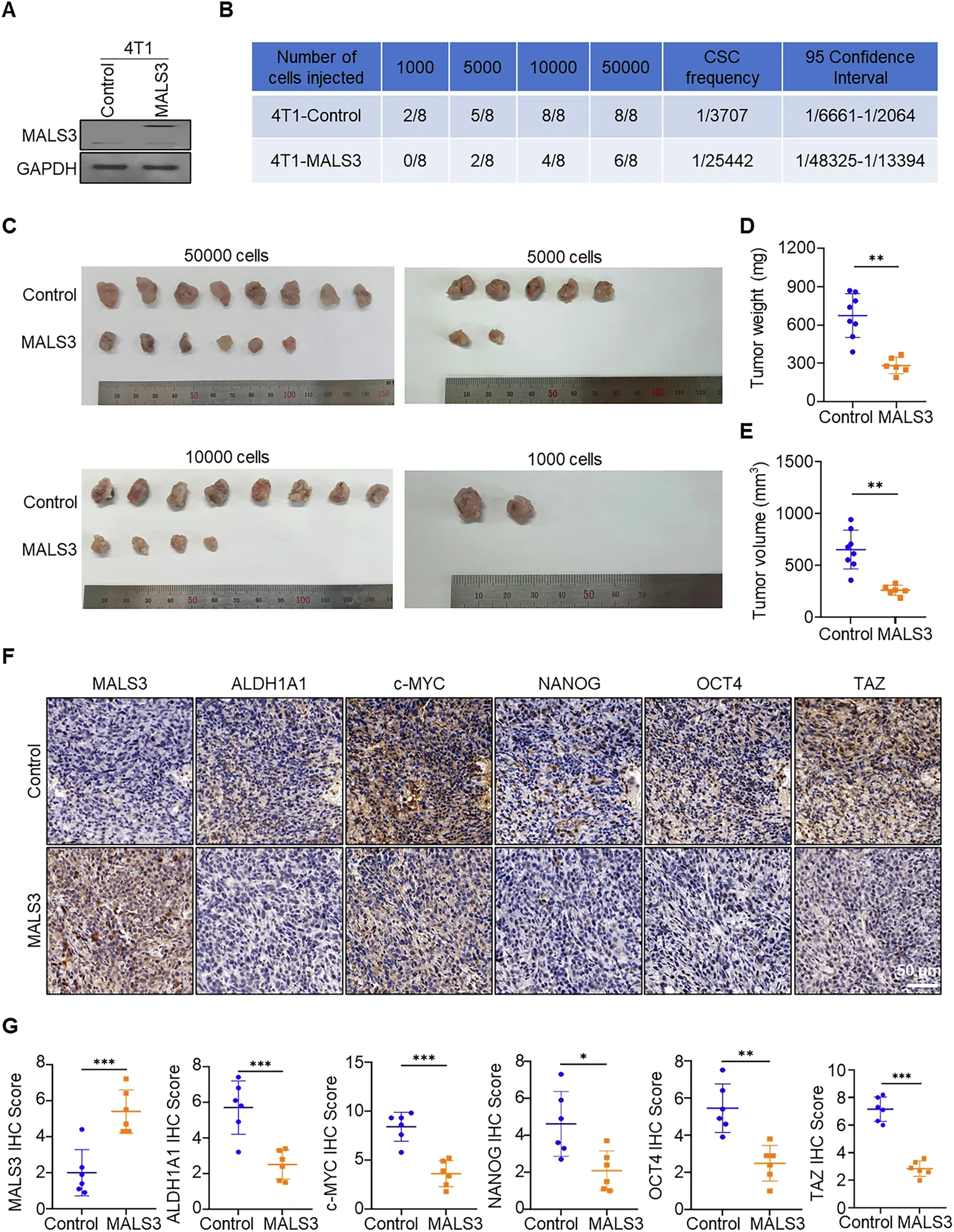
MALS3 suppresses stem cell-like characteristics of breast cancer in vivo. **A** Establishment of stable 4T1 cell lines overexpressing MALS3 and western blotting analysis. **B** Statistical table of tumor formation rates in mice across different cell number groups during the tumor formation assay. **C** Schematic representations of tumors in control and MALS3-overexpressing mice when inoculated with 5 × 10⁴/1 × 10⁴/5 × 10³/1 × 10³ cells per mouse (*n* = 8). Statistical assessment of tumor weight (**D**) and volume (**E**) in control and MALS3-overexpressing mice when inoculated with 5 × 10⁴ cells per mouse. **F** Representative immunohistochemical images of tumor tissues from mice inoculated with 5 × 10⁴ cells per mouse in the tumor formation dilution assay, showing staining for MALS3, ALDH1A1, c-MYC, NANOG, OCT4 and TAZ in tumors from control or MALS3-overexpressing mice. Scale bar: 50 μm. **G** Immunohistochemical staining for MALS3, ALDH1A1, c-MYC, NANOG, OCT4 and TAZ in tumor tissues from control or MALS3-overexpressing mice inoculated with 5 × 10⁴ cells per mouse, with quantitative scoring of results. All data are indicated as mean ± SD. Abbreviation: *P < 0.05, **P < 0.01, ***P < 0.001. Statistical comparisons were conducted using by two-tailed unpaired t-test (**D**, **E**, **G**).

For a deeper exploration of MALS3’s function in vivo, we orthotopically transplanted 1 × 10³–5 × 10⁴ 4T1 cells into the mammary fat pads of female Balb/c mice (Fig. 4A). In comparison to the control group, MALS3-overexpressing group exhibited fewer tumor formations. When 1 × 10³ cells were injected, two control mice developed tumors, whereas no tumor development was observed in the overexpression group. When 5 × 10⁴ cells were injected, only 6 out of 8 MALS3-overexpressing mice formed tumors, compared to all control mice developing tumors. Statistical analysis of these assay results and assessment of CSC frequency indicated that MALS3 overexpression reduced CSC frequency (Fig. 4B, C). Consistent with this, tumor weight and volume were significantly decreased in MALS3-overexpressing mice compared with control mice when inoculated with 5 × 10^4^ cells per mouse (Fig. 4D, E). Immunohistochemical analysis showed decreased expression of CSC-associated biomarkers ALDH1A1, c-MYC, NANOG, OCT4 and TAZ, in tumors from control and MALS3-overexpressing mice inoculated with 5 × 10^4^ cells per mouse (Fig. 4F, G). Consequently, these results illustrate that MALS3 suppresses tumorigenesis in vivo.

### MALS3 enhances the interaction between LATS1 and TAZ through the L27 domain

**Fig. 5:**
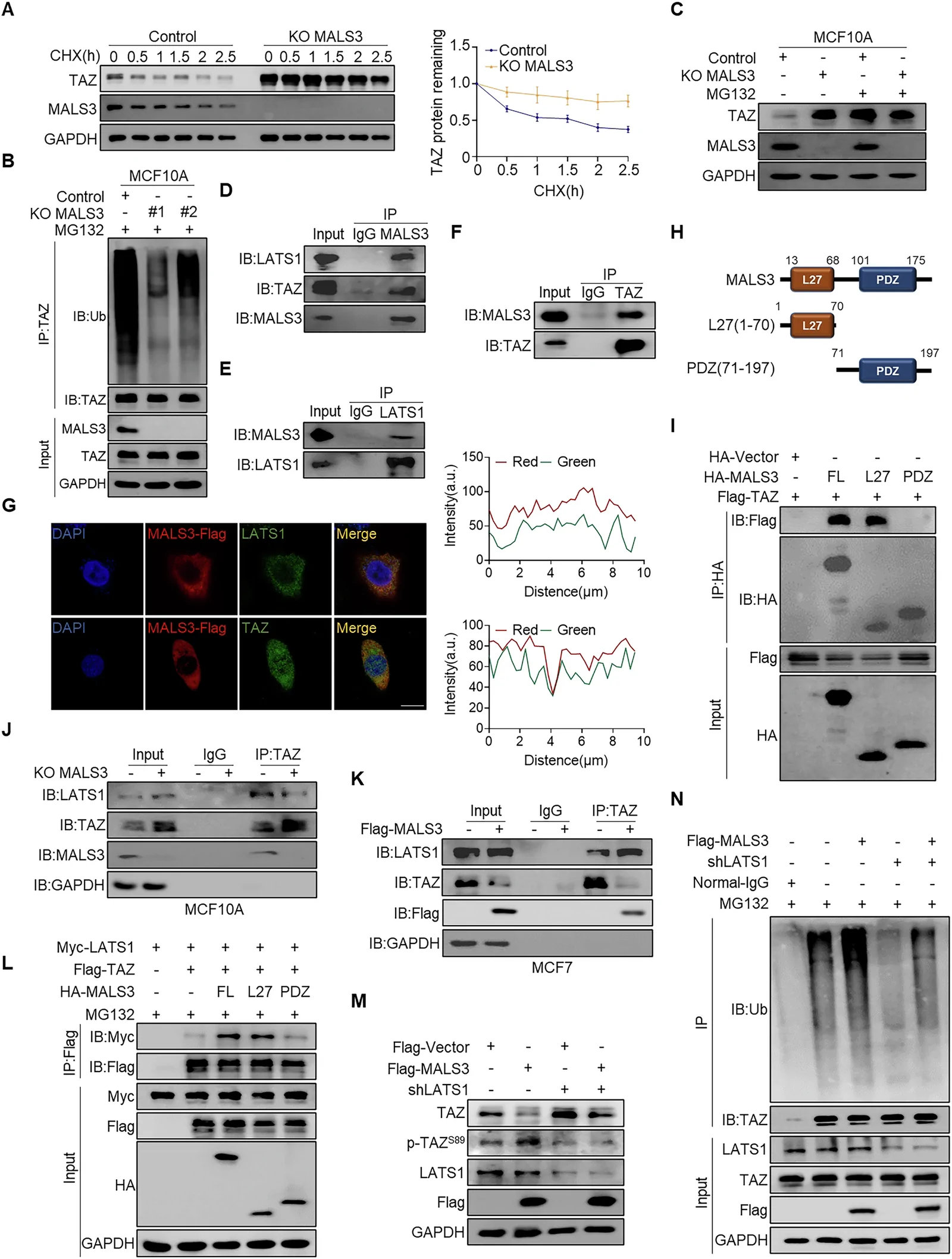
MALS3 enhances the LATS1-TAZ interaction through the L27 domain. **A** Cells (control or MALS3 knockout) were exposed to 100 μg/mL cycloheximide (CHX), followed by western blotting and quantification. **B** MG132 (10 μM) treatment in MALS3 knockout cells followed by TAZ ubiquitination levels detected by anti-TAZ antibody precipitation. **C** TAZ protein expression detected by western blotting in control, MALS3 knockout cells with or without MG132 (10 μM) treatment. Co-immunoprecipitation (co-IP) assays using antibodies against MALS3 (**D**), LATS1 (**E**), and TAZ (**F**) in MCF10A cell lysates to detect interactions among MALS3, LATS1 and TAZ. **G** Immunofluorescence colocalization staining of MALS3-Flag (red) and LATS1 (green), MALS3-Flag (red) and TAZ (green) in MCF7 cells. Scale bar: 10 μm. **H** Schematic illustration of MALS3 truncate variants used in this study. **I** Co-IP assays and immunoblotting performed in HEK293T cells transfected with different plasmids. **J**, **K** Co-IP assays and immunoblotting in MCF10A and MCF7 cells, respectively, under MALS3 knockout or overexpression conditions. **L** Co-IP assays and immunoblotting in HEK293T cells receiving transfection with different plasmids, followed by treatment with MG132 (10 μM). **M** Proteins were collected from MCF7 cells after LATS1 knockdown in both control and MALS3-overexpressing groups for Western blotting. **N** Cells were treated with the proteasome inhibitor MG132 to inhibit the proteasomal degradation of ubiquitinated TAZ, thereby uncoupling the sequential processes of ubiquitination and subsequent protein degradation to accurately detect changes in TAZ ubiquitination levels. Proteins were collected from MCF7 cells after LATS1 knockdown in both control and MALS3-overexpressing groups, treated with MG132, then subjected to co-IP assay and subsequent immunoblotting. Results in Figs. **A–F**, **I–N** were consistent across three independent assays.

To determine whether MALS3 regulates TAZ at the transcriptional or protein level, we first detected that MALS3 knockout or overexpression primarily affected YAP/TAZ at the protein level, with no significant impact at the mRNA level (Fig. S4A, B). More interestingly, we observed that upon cycloheximide (CHX) treatment, MALS3 knockout significantly extends the half-life of TAZ and thereby stabilizes its expression. Conversely, MALS3 overexpression markedly shortens the half-life of TAZ (Figs. 5A and S4C). Moreover, to determine whether MALS3 affects TAZ ubiquitination, we detected TAZ ubiquitination in MALS3 knockout or overexpression cell lines treated with the proteasome inhibitor MG132. We found reduced TAZ ubiquitination in MALS3 knockout cells and increased TAZ ubiquitination in MALS3 overexpression cells (Figs. 5B and S4D, E). Consistent with current findings, MALS3 knockout increased TAZ protein stability, but this effect was eliminated upon MG132 treatment. The MALS3 overexpression group exhibited downregulated TAZ expression, whereas MG132 treatment rescued TAZ protein levels (Figs. 5C and S4F, G).

Our research reveals that MALS3 promotes the proteasomal degradation of TAZ. This leads us to hypothesize that MALS3 may influence TAZ by participating in the canonical Hippo pathway. Previous studies have confirmed that LATS1, a core kinase in the Hippo signaling pathway, negatively regulates TAZ’s nuclear transcription activity by phosphorylating TAZ, trapping it in the cytoplasm, and facilitating its degradation [40]. Co-immunoprecipitation (co-IP) experiments verified interactions between MALS3 and both LATS1 and TAZ (Fig. 5D-F). Meanwhile, we observed an interaction between MALS3 and LATS2, as well as between MALS3 and YAP (Fig. S5A and B). In addition, IF staining in MCF7 cells visually confirmed the colocalization of MALS3 with both LATS1 and TAZ (Fig. 5G). To identify the critical region in MALS3 for TAZ binding, we constructed different domains of MALS3 (Fig. 5H). Co-IP assays using HA-tagged truncated MALS3 variants confirmed that the L27 domain, previously reported to promote protein interactions and assembly, is essential for MALS3-TAZ binding (Fig. 5I). More interestingly, co-IP assays in cells with MALS3 knockout or overexpression showed that MALS3 knockout reduced LATS1-TAZ interaction compared to controls, while MALS3 overexpression enhanced LATS1-TAZ interaction (Figs. 5J, K and S5C, D). Then, our study showed that the enhancement of LATS1-TAZ interaction by MALS3 depends on its L27 domain, further demonstrating the critical role of the L27 domain in LATS1-TAZ interaction (Figs. 5L and S5E). Although MALS3 promotes LATS1-TAZ interaction, we observed that silencing LATS1 in MALS3-overexpressing cells failed to fully restore the reduction in endogenous total TAZ and the enhancement of TAZ ubiquitination, while the increase in phosphorylated TAZ was eliminated (Fig. 5M, N). In summary, these data suggest that the L27 domain of MALS3 is significant in enhancing the LATS1-TAZ interaction.

### MALS3 promotes TRIM29-mediated K48-linked ubiquitination and degradation of TAZ by facilitating TRIM29-TAZ interaction

**Fig. 6:**
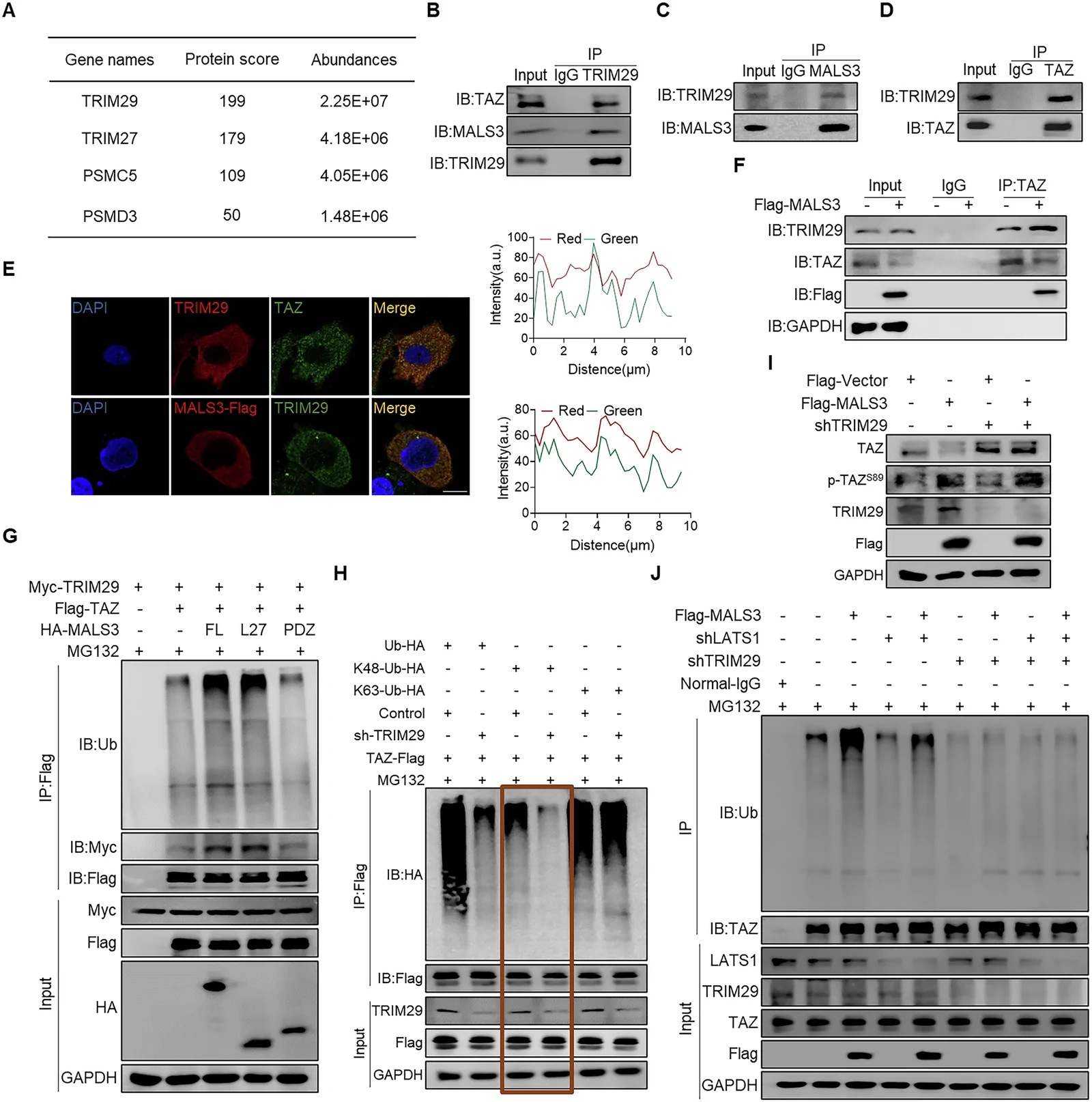
MALS3 promotes TRIM29-mediated K48-linked ubiquitination and degradation of TAZ by facilitating TRIM29-TAZ interaction. **A** Table showing IP-MS results for selected ubiquitin-associated proteins. Co-IP assays using antibodies against TRIM29 (**B**), MALS3 (**C**), and TAZ (**D**) in MCF10A cell lysates to detect interactions between TRIM29, MALS3, and TAZ. **E** Immunofluorescence colocalization staining of TRIM29 (red) and TAZ (green), and MALS3-Flag (red) and TRIM29 (green)in MCF7 cells. Scale bar: 10 μm. **F** Co-IP assays and immunoblotting in MCF7 cells following MALS3 overexpression, respectively. **G**, **H** Co-IP assays and immunoblotting performed in HEK293T cells after transfection with different plasmids followed by MG132 (10 μM) treatment. **I** Western blotting of proteins collected from MCF7 cells after TRIM29 knockdown in control or MALS3-overexpressing cells. **J** After knocking down LATS1, TRIM29, or simultaneously knocking down LATS1 and TRIM29 in control or MALS3-overexpressing MCF7 cells, followed by MG132 treatment, proteins were collected for co-IP assays and immunoblotting. Results in Figs. **B**–**D**, **F**–**J** were consistent across three independent assays.

As described above, MALS3 enhances TAZ phosphorylation levels by promoting the interaction between LATS1 and TAZ, but this is not the primary mechanism affecting total TAZ protein and its ubiquitination. We sought to further investigate other key molecules involved in the impact of MALS3 on TAZ. Analysis of ubiquitin-associated proteins in IP-MS results revealed that the E3 ubiquitin ligase TRIM29 exhibited the highest protein score and protein abundance (Fig. 6A). We verified that endogenous MALS3 interacts with TRIM29 in MCF10A cells, and endogenous TRIM29 interacts with TAZ (Fig. 6B–D). Subsequently, IF assays confirmed the colocalization of MALS3 and TRIM29, as well as the colocalization of TRIM29 and TAZ (Fig. 6E). In MCF10A and MCF7 cells with MALS3 knockout or overexpression, co-IP assays confirmed that MALS3 knockout reduced the interaction between TRIM29 and TAZ, while MALS3 overexpression enhanced this interaction (Figs. 6F and S5F). Additionally, we demonstrated that MALS3 enhanced the interaction between TRIM29 and TAZ via its L27 domain, thereby increasing K48-linked polyubiquitination of TAZ (Fig. 6G, H). In control cells, knocking down TRIM29 elevated total TAZ protein levels. In cells overexpressing MALS3, TRIM29 knockdown restored the reduction in total TAZ protein caused by MALS3 overexpression (Fig. 6I). Similarly, TRIM29 knockdown in control cells decreased TAZ ubiquitination levels. In MALS3-overexpressing cells, knocking down TRIM29 or co-knocking down TRIM29 and LATS1 eliminated the MALS3-induced increase in TAZ ubiquitination (Fig. 6J). Thus, we demonstrated that the E3 ubiquitin ligase TRIM29 regulates the degradation of TAZ via K48-linked polyubiquitination, and in this process, MALS3 can reduce TAZ stability in cancer by promoting the interaction between TRIM29 and TAZ.

### Mirtazapine as an upregulator of MALS3 enhances the therapeutic efficacy of chemotherapy drugs

**Fig. 7:**
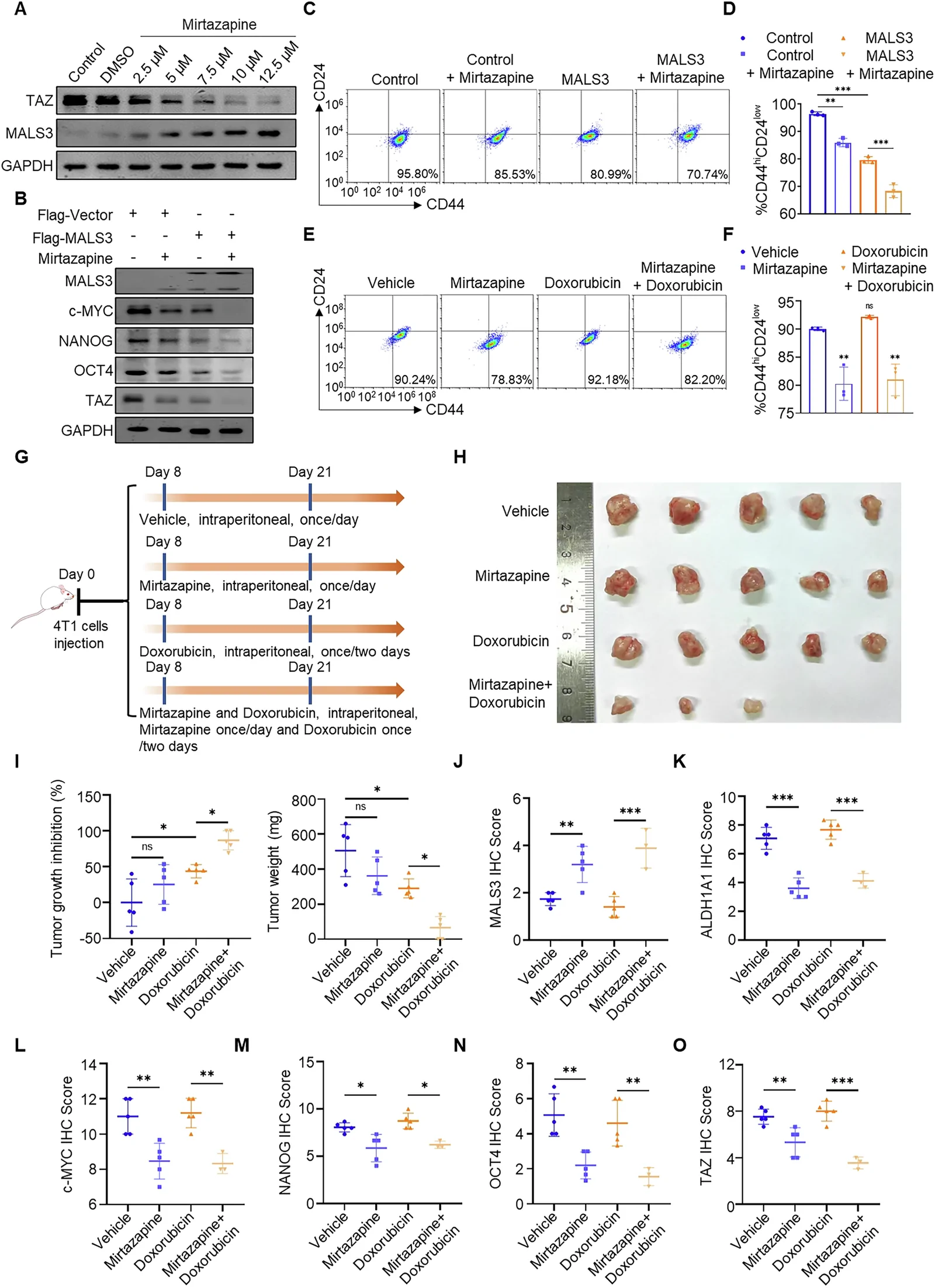
Mirtazapine as an upregulator of MALS3 enhances the therapeutic efficacy of chemotherapy drugs. **A** Proteins were collected from 4T1 cells exposed to different doses of mirtazapine for Western blotting. Control group: untreated 4T1 cells. **B** Add mirtazapine (10 μM) to control and MALS3-overexpressing 4T1 cells, then collect proteins for western blotting. **C**, **D** Add mirtazapine to control and MALS3-overexpressing 4T1 cells, then use flow cytometry to detect and quantify the percentage of the CD44^+^CD24^−^ subset. **E**, **F** After adding mirtazapine, doxorubicin, or combined mirtazapine and doxorubicin to 4T1 cells, the percentage of the CD44^+^CD24^−^ subset was detected and quantified by flow cytometry. **G** Schematic diagram of the drug-treated animal assays. **H** Schematic representation of tumors in mice from the drug-treated animal assay (*n* = 5). **I** Statistical chart of tumor volume inhibition rate and tumor weight in mice. **J–O** Immunohistochemical staining for MALS3, ALDH1A1, c-MYC, NANOG, OCT4, and TAZ was performed on tumor tissues from mice in different groups, followed by scoring and quantification. The drug concentrations used in the in vivo experiments were mirtazapine (10 mg/kg), doxorubicin (1 mg/kg). All data are indicated as mean ± SD. ns not significant; *P < 0.05, **P < 0.01, ***P < 0.001. Statistical comparisons were performed using by one-way ANOVA (**D**, **F**, **I**–**O**).

Upon which we wanted to explore the clinical application of MALS3. Based on former research, the antidepressant drug mirtazapine has been demonstrated to antagonize the 5-hydroxytryptamine 2C receptor (HTR2C), thereby upregulating its downstream molecule MALS3 and inhibiting tumor metastasis via the MALS3/β‑catenin pathway [41]. Thus, we assessed the effects of different mirtazapine concentrations on MALS3 upregulation and TAZ inhibition. Significant effects were observed at a mirtazapine concentration of 10 μM without obvious off‑target effects (Figs. 7A and S6A). Meanwhile, we also verified the inhibitory effect of mirtazapine on tumor metastasis in breast cancer cells, further supporting the findings of previous research (Fig. S6B, C). Consistently, adding mirtazapine to both control and MALS3-overexpressing cells led to differing extents of decrease in the protein levels of CSC-related molecules and the target gene TAZ (Figs. 7B and S6D, E). Flow cytometry results further showed that mirtazapine effectively reduced the percentage of CD44^+^CD24^−^ cell subset (Fig. 7C, D). Given our earlier finding that MALS3 overexpression enhances breast cancer cells’ sensitivity to doxorubicin chemotherapy, we sought to determine the efficacy of combining mirtazapine with doxorubicin. We then assessed the therapeutic outcomes of the combinatorial approach in vivo and in vitro. Compared to cells exposed to doxorubicin alone, the combination of mirtazapine and doxorubicin effectively reduced the proportion of the CD44^+^CD24^−^ cell subpopulation (Fig. 7E, F). Similar to in vitro results, in vivo experiments showed that addition of mirtazapine dramatically enhanced the tumor growth-inhibiting efficacy of doxorubicin compared to doxorubicin monotherapy (Fig. 7G–I). Immunohistochemical results in tumor tissues from mice revealed that adding mirtazapine effectively increased MALS3 expression while significantly decreasing the expression of CSC-associated molecules ALDH1A1, c-MYC, NANOG, OCT4, and TAZ (Fig. 7J–O and S6F). Meanwhile, the HE staining results of the mice’s internal organs showed that the combined treatment of mirtazapine and doxorubicin did not produce serious toxic side effects, demonstrating good biological safety (Fig. S6G). Together, these data indicate that MALS3 upregulator mirtazapine potentiates the therapeutic effect of the chemotherapeutic agent doxorubicin in inhibiting tumor growth.

## Discussion

Disruption of polarity proteins is recognized as a crucial driver of tumorigenesis, progression, and malignancy [1, 42, 43]. Previous studies have shown that CRB3, a core component of the CRB complex, functions as a tumor suppressor. As well as in breast cancer, CRB3 serves as a crucial regulator of stem cell-like properties through suppression of β-catenin pathway to reverse tamoxifen resistance [44–46]. This study focuses on another key molecule within the CRB complex known as the polarity protein MALS3, which contains both L27 and PDZ domains [47]. The L27 domain exerts a vital function in mediating protein-protein interactions between molecules, promoting protein complex assembly [48–50]. Current research on MALS3, except for its methylation regulation in oral squamous cell carcinoma and metastasis-inhibitory effects mediated by the MALS3/β-catenin pathway, has not fully explored its role in regulating CSCs [41, 51]. Here, we demonstrated that MALS3 is downregulated in breast cancer cells, particularly in luminal and TN subtypes. MALS3 expression levels negatively correlate with poor prognosis in breast cancer patients. Intriguingly, analyzing data from human breast cancer tissues confirmed that MALS3 also exhibits an inverse association with the protein expression of ALDH1A1, a marker specific to BCSCs. Moreover, MALS3 overexpression suppresses breast cancer stem cell-like characteristics and reduces tumorigenicity in vivo assays across varying concentration gradients. These findings suggest MALS3 may exert tumor-suppressing effects in breast cancer by suppressing CSCs.

BCSCs are increasingly recognized as a pivotal driver of therapeutic resistance, tumor recurrence, and metastatic advancement in breast cancer. These cells can undergo self-regeneration, multipotent differentiation potential, and unique capacity to initiate and maintain tumor development [52–54]. A crucial mechanism underlying their resilience lies in their frequent quiescence, which is beneficial for them to evade conventional chemotherapeutics such as doxorubicin [55, 56]. On the other hand, the ABC transporter family abundantly expressed in CSCs, including the drug resistance protein encoded by ABCG2 and P-glycoprotein encoded by ABCB1, pumps chemotherapy drugs that have entered the cells out using energy generated by ATP hydrolysis. This reduces the cytotoxic impacts of chemotherapy medications on CSCs, potentially resulting in tumor recurrence and growth [57–59]. For instance, in GBM, CSCs in G0 phase dormancy exhibit insensitivity to chemotherapy drugs. Following treatment, they reactivate and reinitiate the cell cycle, thereby promoting tumor proliferation [55]. Addressing the treatment resistance observed with doxorubicin, a commonly used chemotherapy drug in breast cancer therapy, combining drugs that eliminate CSCs with doxorubicin holds promise as an effective strategy to overcome drug resistance [60, 61]. In this present study, we verified that MALS3 shows notable suppression effects on BCSCs both in vivo and in vitro. Our findings reveal the therapeutic prospect of targeting BCSCs via MALS3 upregulation and represent mirtazapine as a promising candidate for repurposing in breast cancer therapy. More significantly, the combination of mirtazapine and doxorubicin enhances breast tumor sensitivity to doxorubicin treatment and inhibits tumor growth, this strategy may offer a clinically translatable potentiality to overcoming chemoresistance.

As a co-activator of transcription, TAZ acts as a crucial downstream effector of the Hippo signaling pathway, playing a pivotal function in modulating stem cell proliferation, differentiation, and survival [62–64]. TAZ is vital for maintaining CSC-like characteristics, contributing to tumor origination and therapeutic resistance. Classical Hippo signaling restrains TAZ activity through a kinase cascade that promotes its cytoplasmic retention and subsequent β-TRCP-driven ubiquitination and proteasomal degradation [65, 66]. Remarkably, current research on TAZ predominantly focuses on its classical inactivation and degradation pathway, but still exists many non-classical crucial TAZ regulatory factors deserve to be studied. Here, we elucidated that the E3 ubiquitin ligase TRIM29 acts on TAZ as a novel regulatory factor and mediates its degradation through K48-linked polyubiquitination. And this regulatory mechanism operates independently of the classical Hippo pathway. Interestingly, MALS3 further reduces total TAZ protein levels by promoting the binding between TRIM29 and TAZ. Data from human breast cancer tissues and mouse tumors indicate that MALS3 suppresses TAZ protein abundance, further supporting our findings. Additionally, we identified that MALS3 promotes the binding of the kinase LATS1 to TAZ, thereby increasing TAZ phosphorylation modification, though this has limited impact on total TAZ protein levels. Collectively, our results uncover a dual mechanism through which MALS3 regulates TAZ. In addition to the classical regulatory pathway, our work unveils a novel regulatory axis MALS3-TRIM29-TAZ that operates in BCSCs suppression, offering new insights into therapeutic targeting of TAZ-driven chemoresistance.

**Fig. 8:**
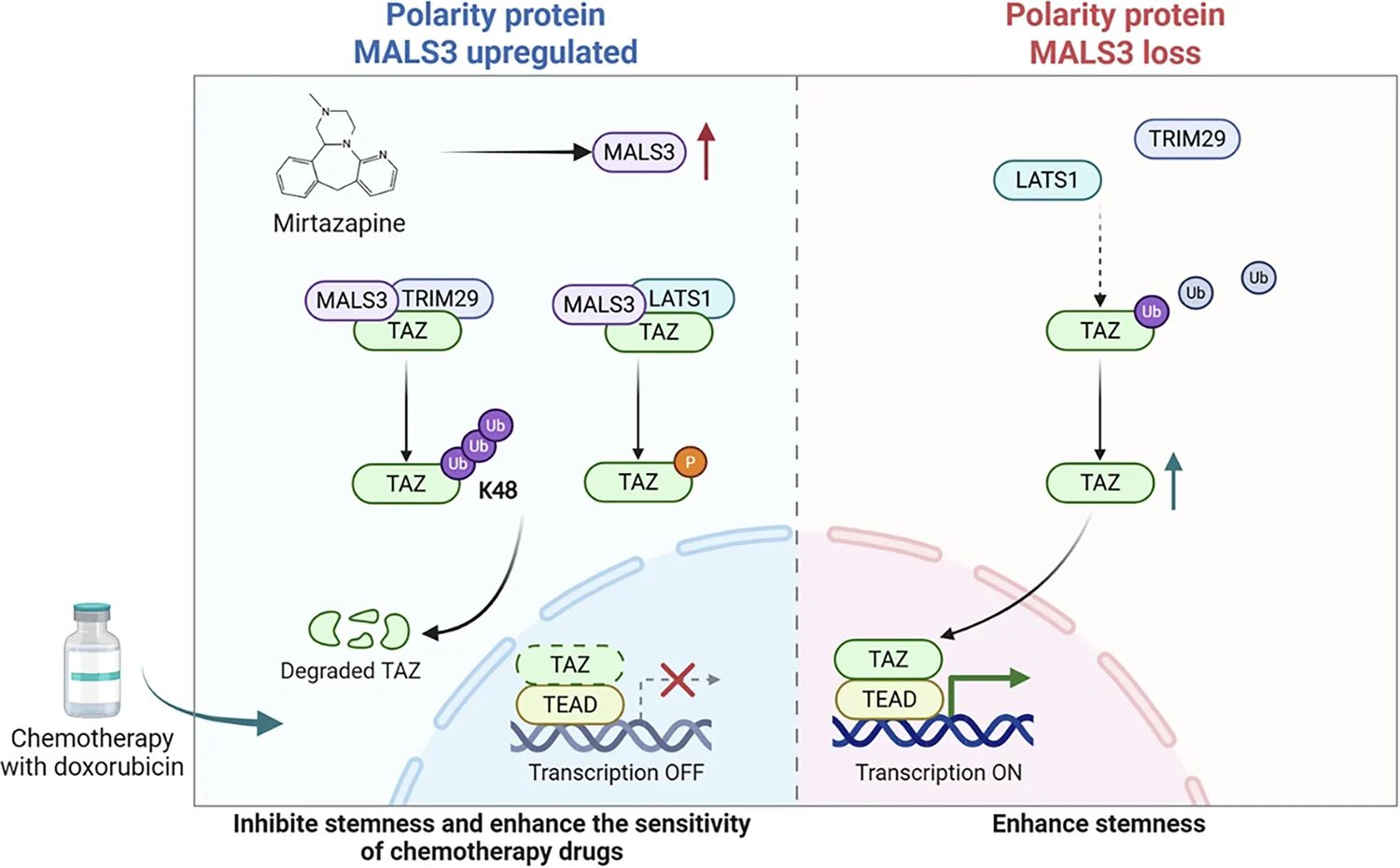
Schematic model of mirtazapine upregulated MALS3 inhibiting TAZ and cancer stem cell-like properties to enhance chemosensitivity in breast cancer. MALS3 reduces cancer stem cell-like properties through enhancing the interaction of kinase LATS1 or E3 ubiquitin ligase TRIM29 with TAZ, thereby increasing TAZ phosphorylation and K48-linked polyubiquitination. The MALS3 upregulator mirtazapine effectively elevates MALS3 protein levels, and its combination with the chemotherapeutic agent doxorubicin enhances tumor sensitivity to chemotherapy drugs. Created with BioRender.com.

In summary, our study reveals that the polarity protein MALS3 is a crucial signaling hub and a valuable node for overcoming treatment resistance in breast cancer by suppressing BCSCs. We identified a dual mechanism where MALS3 enhances the phosphorylated modification of TAZ via LATS1 through the L27 domain, while simultaneously recruiting the E3 ligase TRIM29 to induce TAZ degradation via K48-linked polyubiquitination. We further discovered that mirtazapine, as an MALS3 upregulator, effectively increases MALS3 protein levels and enhances breast cancer cells’ sensitivity to chemotherapeutic drugs (Fig. 8). This work not only reveals a new regulatory pathway but also proposes a direct strategy for therapeutic utilization through drug repurposing, providing a direct clinical translation approach to overcome chemotherapy resistance.

## Materials and methods

### Cell lines

The normal breast and BC cell lines used in this experiment were acquired from Procell Life Science and Technology (Wuhan, China). MCF7 and HEK293T cells were grown in DMEM medium supplemented with 10% FBS. MCF10A cells were grown in DMEM/F12 medium supplemented with 5% HS, 20 ng/ml human EGF, 10 μg/ml insulin, and 0.5 μg/ml hydrocortisone. BT549 cells were maintained in RPMI 1640 medium supplemented with 10% FBS and 1 μg/ml insulin. 4T1 cells were maintained in RPMI 1640 medium supplemented with 10% FBS. All cell lines were kept in an incubator at 37 °C with 5% CO₂. Cells were routinely tested for mycoplasma infection, with all results being negative.

### Antibody information

Primary antibodies used were MALS3 (Thermo, #51-5600), LLGL1 (Proteintech, #22496-1-AP), PATJ (Thermo, PA5-85444), MPP5/PALS1 (Proteintech, #17710-1-AP), c-MYC (Abcam, #ab185656), OCT4 (Abcam, #ab181557), NANOG (Abcam, #ab109250), LATS1 (Cell Signaling Technology, #3477), phospho-LATS1^T1079^ (Cell Signaling Technology, #8654), GAPDH (Proteintech, # HRP-60004), YAP (Cell Signaling Technology, #14074), phosphor-YAP^S127^(Cell Signaling Technology, #13619), TAZ (Cell Signaling Technology, #83669), phosphor-TAZ^S89^(Santa Cruz, #sc-17610), LAMIN B (HUABIO, #R1508-1), TRIM29 (Santa Cruz, #sc-376125), TRIM29 (abcam, #ab108627), HA (Sigma, #H6908), Flag (Sigma, #F9291), CD44-APC (Biolegend, #103102), CD24-PE (Biolegend, #311106), CD24-FITC (Biolegend, #W20001B).

### Cell transfection and stable cell line generation

Cell transfection was carried out with PEI (Yeasen), with plasmids transfected into cells according to the manufacturers’ instructions. To establish stable MALS3-knockout cell lines using the CRISPR-Cas9 system, mammary epithelial cells MCF10A were cultured in medium containing 6 μg/mL polybrene and infected with a dual-sgRNA CRISPR/Cas9 lentiviral knockout vector (vector backbone: pLent-2in1-U6-spgRNA-EF-spCas9-P2A-PURO). The sgRNA sequences for KO#1 cell line were sgRNA1: GCTAGGGGAACCCGTGCGGC and sgRNA2: AAAGCCTGAAGTTTCTGTGG. The sgRNA sequences for KO#2 cell line were sgRNA3: CGGCGCTAGGGGAACCCGTG and sgRNA4: TGAACATGTCTATGAGACTG. 48 h after infection, cells were selected in medium supplemented with 2 μg/mL puromycin for 3 consecutive days to obtain stable MALS3-knockout cell lines. To establish MALS3-overexpressing stable cell lines, breast cancer cells were infected with the N-terminal 3×Flag-tagged MALS3 overexpression lentivirus (vector backbone: pLent-CMV-P2A-PURO) in culture medium containing 6 μg/mL polybrene. At 48 h post infection, cells were selected in medium supplemented with 2 μg/mL puromycin for 3 consecutive days, and MALS3-overexpressing stable cell lines were finally obtained.

### Immunohistochemistry

Using standard immunohistochemical methods, specific staining was performed with the corresponding MALS3, ALDH1A1, and TAZ antibodies. Semi-quantitative scoring of staining in each tissue section: Percentage of positively stained cells (<10% = 0 points, 10–25% = 1 point, 25–50% = 2 points, 50–75% = 3 points, >75% = 4 points), and staining intensity (1–3 points). The final score was calculated by multiplying these two scores.

### Bioinformatic analysis of the TCGA dataset

Bioinformatic analysis was conducted based on the TCGA cohort. Gene expression profiles and corresponding clinical follow-up data were obtained from the TCGA database. To investigate the prognostic significance of MALS3, survival analysis was performed. Patients were dichotomized into MALS3-high and MALS3-low expression groups based on an optimal cutoff value determined using the surv_cutpoint function in the “survminer” R package.

### Western blotting

Collect cells and lyse them using Western and IP cell lysis buffer (Beyotime) containing protease inhibitors (MCE) and phosphatase inhibitors (MCE). Centrifuge the obtained lysate at 12,000 *×* *g* for 20 min at 4°C, collect the supernatant, and quantify it using the Quick Start™ Bradford Protein Assay Dye Reagent (Bio-Rad). Subsequently, proteins were mixed with 5× SDS loading buffer and boiled for 10 min to obtain protein samples. Proteins were separated by SDS-PAGE gel electrophoresis and transferred to a PVDF membrane. The membrane was blocked with 5% skim milk powder at room temperature for 1 h, followed by incubation with primary and secondary antibodies. Finally, chemiluminescent signals were detected using ECL (MCE) detection reagent.

### 3D morphogenesis

Add 50 μl of growth factor-reduced matrix gel (Corning, #354230) to each well of an 8-well chamber slide (Thermo, #154534PK). Incubate in an incubator for 15 min to allow solidification. Resuspend cells in experimental basal medium, then mix 1:1 with experimental enhanced medium. Seed the cell suspension onto pre-coated chamber slides, ensuring a final cell concentration of 5000 cells per well. The chamber slide was then placed in an incubator, with medium replaced every 2 days.

### Immunofluorescence

Washed cells once with PBS, then fixed with 4% paraformaldehyde for 10 min. Permeabilized with 0.2% Triton X-100 and blocked with 5% BSA. Samples were incubated with primary antibody overnight at 4 °C. The next day, they were incubated with fluorescently labeled secondary antibody. Finally, samples were stained with DAPI and mounted on glass slides. Image acquisition was performed using a Leica inverted fluorescence microscope and a Leica laser scanning confocal microscope.

### Flow cytometry

After collecting the cells, resuspend them following two washes with PBS. Add the antibody concentration recommended in the manufacturer’s instructions to the cell suspension and incubate at 37 °C in the dark for 30 min. Resuspend cells after two additional PBS washes. Pass the cell suspension through a 70 μm cell strainer and analyze by flow cytometry. Detect the CD44^+^CD24^−^ cell subset using PE- or FITC-CD24 antibody (Biolegend) and APC-CD44 antibody (Biolegend). ALDH^hi^ cells were detected using the ALDHFLUOR assay kit (StemCell Technologies), with cells exposed to the specific ALDH inhibitor diethylaminobenzaldehyde (DEAB) serving as the negative control. Data analysis was carried out using FlowJo 10.6.2 software.

### Cancer mammospheres formation assay

Seed 5000 cells from each treatment group into ultra-low adhesion 6-well plates (Corning, #3471). Culture for one to two weeks using DMEM/F12 medium (Gibco) supplemented with 20 ng/ml epidermal growth factor (Beyotime) and 2% B27 (Invitrogen). Count the number of primary mammospheres, defined as spheres larger than 70 μm. Subsequently, the primary mammospheres were filtered through a 70 μm mesh and the suspension collected. The cells were further digested into single cells using trypsin, then re-seeded and cultured as described above. Finally, the results were tallied and analyzed.

### Cell viability assays

Cells were seeded at a density of 6000 cells per well into 48-well plates. After cell attachment, different concentrations of chemotherapy drugs were added to the control group or stably transfected cell wells. Following 48 h of continuous cell culture, MTT (methylthiazoltetrazolium, Sigma, #298-93-1) was added to the wells according to the manufacturer’s instructions to assess cell viability. Measure absorbance at 490 nm using an Epoch microplate spectrophotometer (BioTek).

### Co-IP and IP-MS analysis

For co-IP analysis, cells were lysed on ice for 20 min using NP40 lysis buffer supplemented with protease inhibitors. Following centrifugation at 12,000 × *g* for 20 min at 4 °C, the supernatant was collected and incubated with pre-coated protein A/G magnetic beads (Thermo, #20423) or magnetic beads pre-coated with tag-specific antibodies. Incubate overnight at 4 °C with gentle rotation. The following day, wash the beads with IP wash buffer. Combine the beads with 1× SDS loading buffer and boil for 10 min to obtain the protein sample. Perform protein loading and band analysis via immunoblotting (IB).

For IP-MS analysis, protein samples obtained via co-IP assays were subjected to overnight digestion with sequencing-grade modified trypsin (Promega Corporation, #PRV5111). The resulting peptides were analyzed using a nano-flow EASY-nLC 1200 system (Thermo) coupled with an Orbitrap Exploris 480 mass spectrometer (Thermo).

### Total RNA extraction and real-time qPCR (RT-qPCR)

Total RNA was extracted from cells using RNAfast 200 Reagent (Fastagen, #220010) according to the manufacturer’s instructions. Subsequently, reverse transcribe it into cDNA using PrimeScript™ RT Master Mix (Takara, #RRO36A) according to the manufacturer’s instructions. Quantify the relative levels of mRNA using RT-qPCR with SYBR-Green Reagent Kit (Tsingke, #TSE202).

### Luciferase reporter assay

Seed cells at a density of 20,000 cells per well in a 24-well plate. Then, cells were co-transfected with 1 μg TEAD firefly luciferase reporter (Addgene) and 0.5 μg sea cucumber luciferase plasmid using Lipofectamine 2000 (Invitrogen). After 48 h, perform the dual luciferase assay (Yeasen) according to the manufacturer’s instructions. Standardize TEAD firefly luciferase activity relative to sea urchin luciferase activity as an internal control.

### In vivo experiments

Animal experiments were approved by the Ethics Committee of Xi’an Jiaotong University. Animals were randomly assigned to experimental groups. In the limited dilution experiments, varying numbers of cells were resuspended in 100 μl serum-free medium, mixed with matrix gel in a 1:1 ratio, and then implanted into the right fourth mammary fat pad of 6-week-old Balb/c female mice. Tumor incidence was assessed via palpation and mammary surgical incision examination. Tumor volume = (long axis × short axis^2^)/2, and tumor weight was measured. In the treatment experiments, 5 × 10^4^ cells were resuspended in 100 μl serum-free medium, mixed with matrix gel, and then implanted. Mice were treated 1 week post-inoculation via intraperitoneal injection with solvent control, mirtazapine (10 mg/kg), doxorubicin (1 mg/kg), or combined mirtazapine + doxorubicin. Tumor volume and weight were assessed as outlined previously.

### Statistics

Figures were analyzed and generated using GraphPad Prism 8 software. Data presentation and statistical analyses were described in the Figure legends. All data are presented as mean ± standard deviation or as specific values as appropriate. Differences between groups were compared using t-tests or two-way analysis of variance (ANOVA) in GraphPad Prism 8. We considered *P* < 0.05 to be statistically significant.

## Supplementary information

Supplementary figures

Supplementary table

Original western blots

## Data Availability

All data supporting the findings described in this manuscript are available upon reasonable request to the corresponding author. Relevant data are available from the corresponding author upon reasonable request.
