# Supplementary material for: Polarity protein MALS3 suppresses breast cancer stem cell-like properties by promoting TAZ inactivation and ubiquitination-dependent degradation
Source: Cell Death Dis. 2026 Jun 21;17(1):947. doi: 10.1038/s41419-026-09010-7 (PMC13743759; doi:10.1038/s41419-026-09010-7)
Supplement: Supplementary file 1 [file 41419_2026_9010_MOESM1_ESM.pdf]

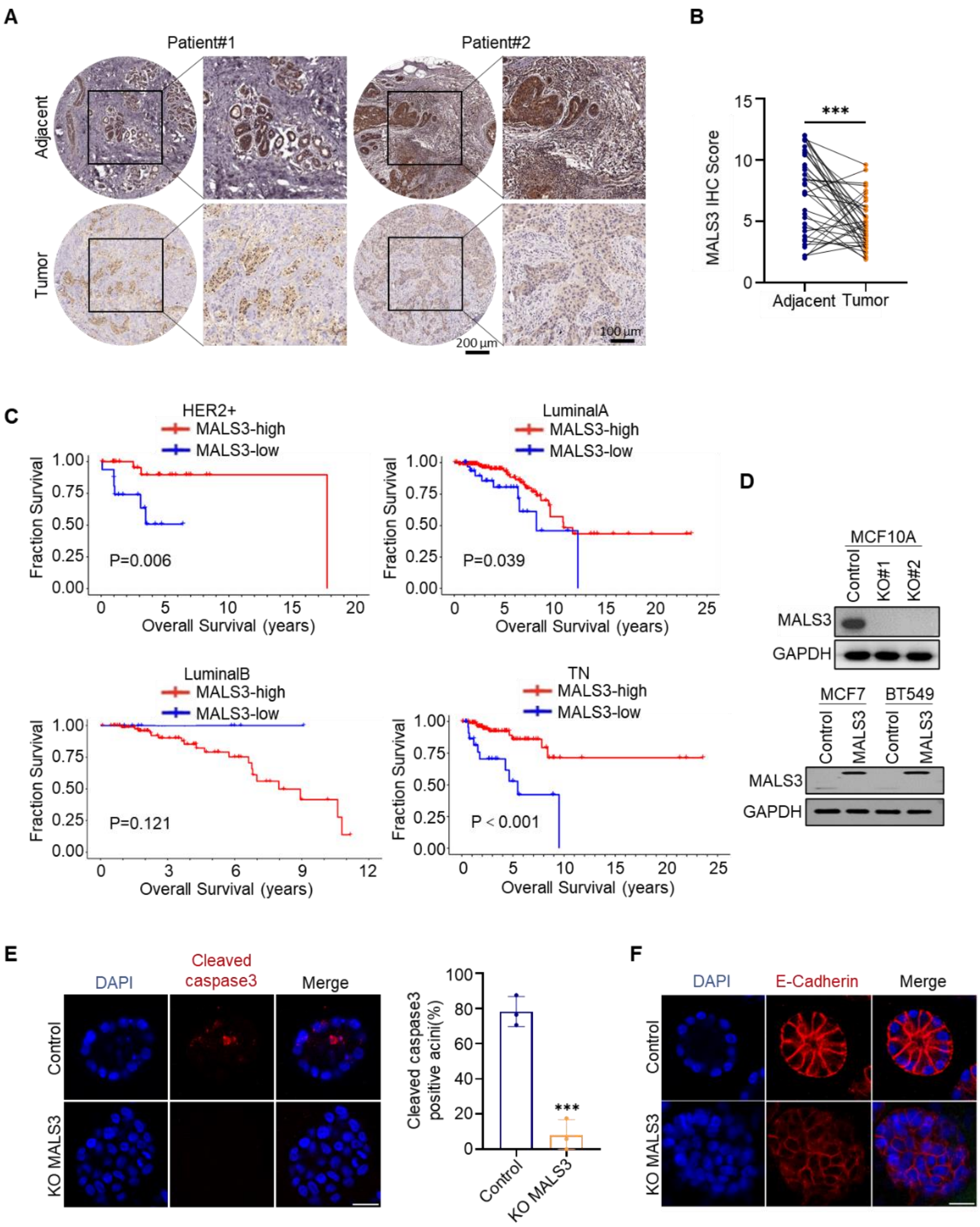

Fig. S1 (A) Representative images of immunohistochemical staining in paired breast cancer and adjacent breast tissue specimens. Scale bar: ×20 field of view = 200 μm; ×40 field of view = 100 μm. **(B)** Quantification of MALS3 immunohistochemical scores in paired breast cancer and adjacent breast tissue samples. **(C)** Survival analysis was performed on breast cancer cases of different subtypes derived from the TCGA breast cancer dataset. **(D)** Western blotting for MALS3 expression levels in MCF10A, MCF7, and BT549 cells with MALS3 knockout or overexpression. **(E)** Proportion of Cleaved caspase3-positive cells in control or MALS3-knockout MCF10A cells cultured in 3D, analyzed by immunofluorescence. Scale bar: 50 μm. **(F)** E-cadherin staining of control versus MALS3-knockout MCF10A cells cultured in 3D. Scale bar: 50 μm. Data are indicated as the mean ± SD of 3 independent replicates. Abbreviation: ns, not significant. **P*<0.05, ***P*<0.01, ****P*<0.001. Statistical comparison was performed using by two-tailed paired t-test **(B)**, log-rank test (**C**) or two-tailed unpaired t-test **(E)**.

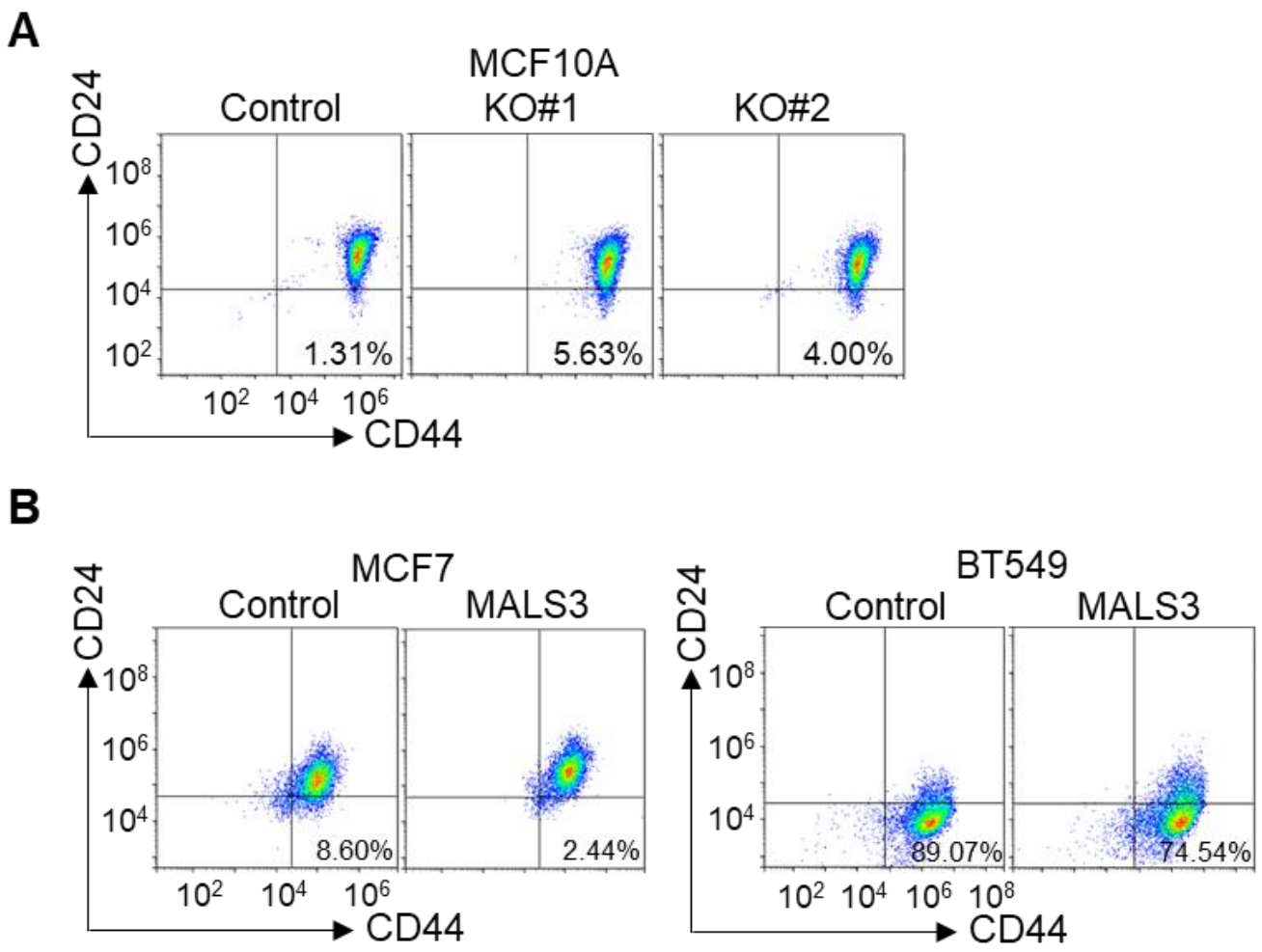

Fig. S2 (A) Flow cytometric analysis of the proportion of $CD44^{+}CD24^{-}$ subpopulations in MALS3-knockout MCF10A cells. **(B)** Flow cytometric analysis of the proportion of $CD44^{+}CD24^{-}$ subpopulations in MALS3-overexpressing MCF7 and BT549 cells. Data are indicated as the mean ± SD of 3 independent replicates.

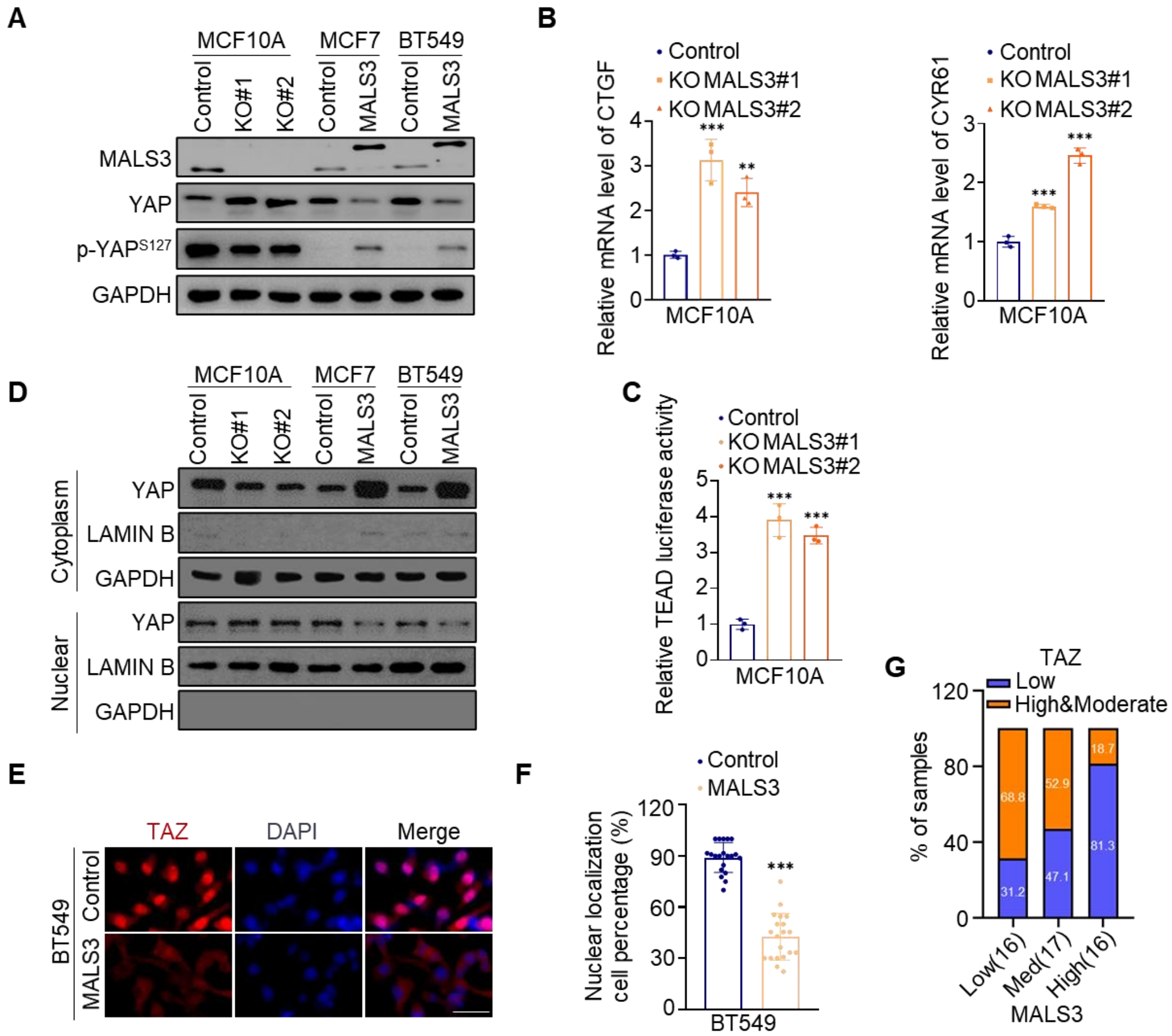

Fig. S3 (A) Western blotting analysis in MCF10A, MCF7, and BT549 cells with MALS3 knockout or overexpression. **(B)** Detection of mRNA levels of TAZ downstream target genes CTGF and CYR61 following MALS3 knockout via RT-qPCR. **(C)** Luciferase reporter assay of TEAD following MALS3 knockout. **(D)** Western blotting analysis of YAP expression in the nucleus and cytoplasm. **(E** and **F)** Immunofluorescence assay and quantification of TAZ subcellular localization. Scale bar: 50 μm. **(G)** Immunohistochemical scoring analysis of TAZ expression levels in breast cancer tissue samples with varying MALS3 expression levels. Data are indicated as the mean ± SD of 3 independent replicates. Abbreviation: ***P*<0.01; ****P*<0.001. Statistical comparisons were performed using a two-tailed unpaired t-test **(B**, **C** and **F)**.

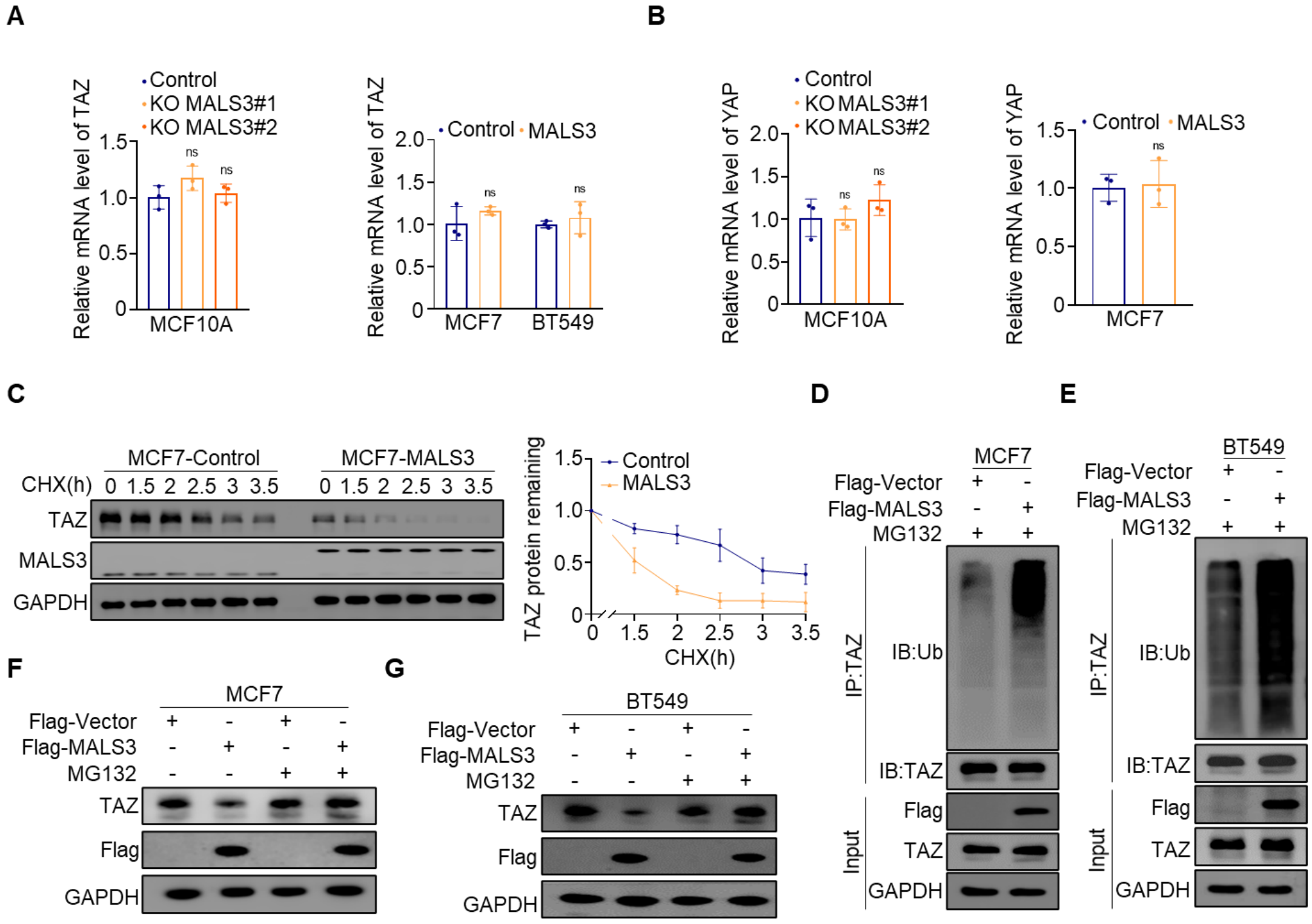

Fig. S4 (A) TAZ mRNA levels were assessed via RT-qPCR after MALS3 knockout or overexpression. **(B)** Detection of YAP mRNA levels following MALS3 knockout or overexpression. **(C)** Cells (control or overexpression) were exposed to cycloheximide, followed by Western blotting and quantification. **(D** and **E)** MG132 treatment in MALS3 overexpressed cells followed by TAZ ubiquitination levels detected by anti-TAZ antibody precipitation. **(F** and **G)** TAZ protein expression detected by Western blotting in control, MALS3 overexpressed cells with or without MG132 (10μM) treatment. Data are indicated as the mean ± SD of 3 independent replicates. Abbreviation: ns, not significant. Statistical comparisons were performed using a two-tailed unpaired t-test **(A** and **B)**.

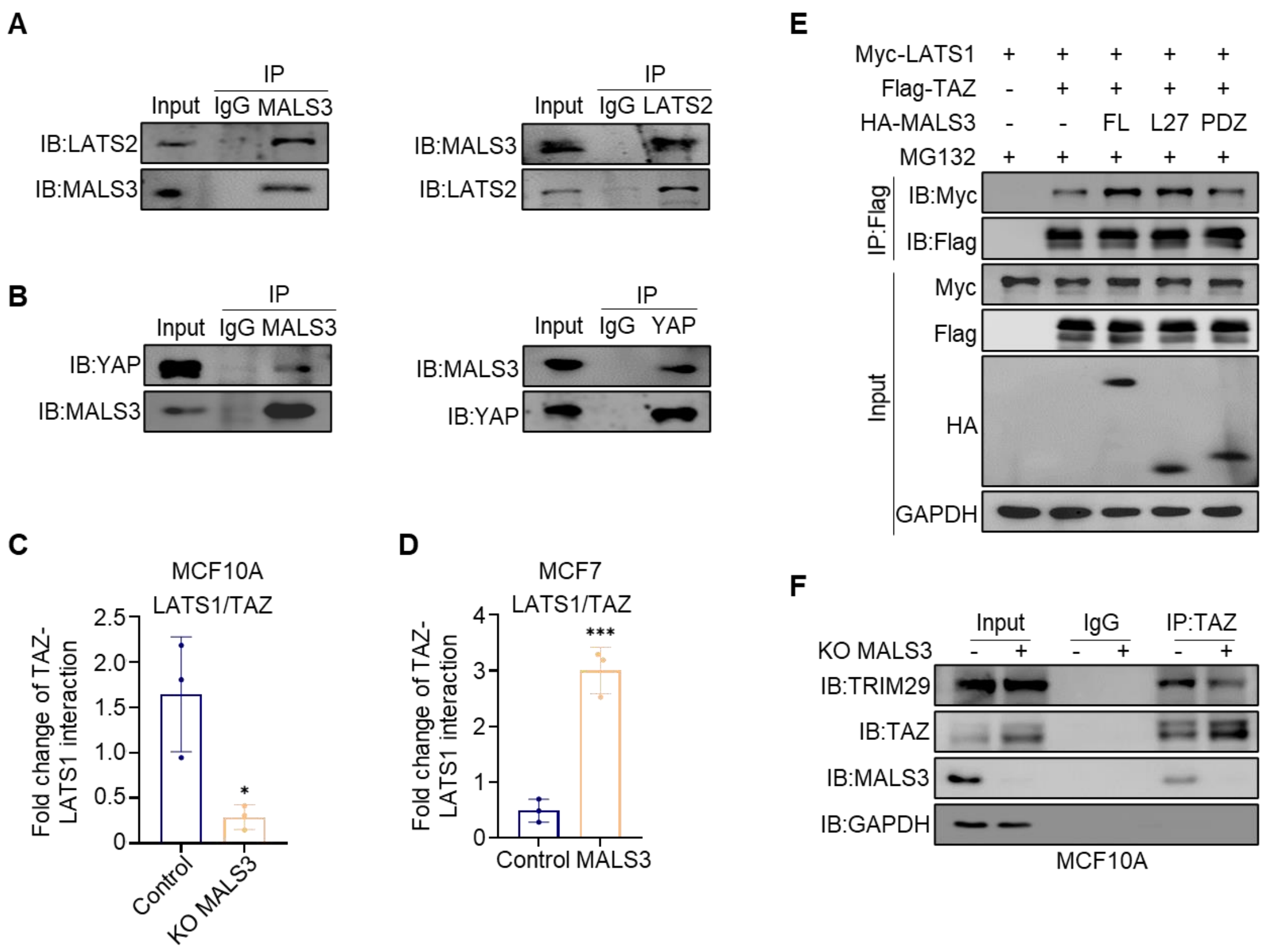

Fig. S5 (A) Co-immunoprecipitation (co-IP) experiments using anti-MALS3 and anti-LATS2 antibodies to detect interactions between MALS3 and LATS2. **(B)** Co-IP experiments using anti-MALS3 and anti-YAP antibodies to detect interactions between MALS3 and YAP. **(C and D)** Quantification of the TAZ–LATS1 interaction normalized to total TAZ. **(E)** Co-IP experiments followed by immunoblotting in MCF7 cells transfected with different plasmids and treated with MG132. **(F)** Co-IP experiments and immunoblotting performed under MALS3 knockout conditions. Data are indicated as the mean ± SD of 3 independent replicates. Abbreviation: **P*<0.05; ****P*<0.001. Statistical comparisons were performed using a two-tailed unpaired t-test **(C and D)**.

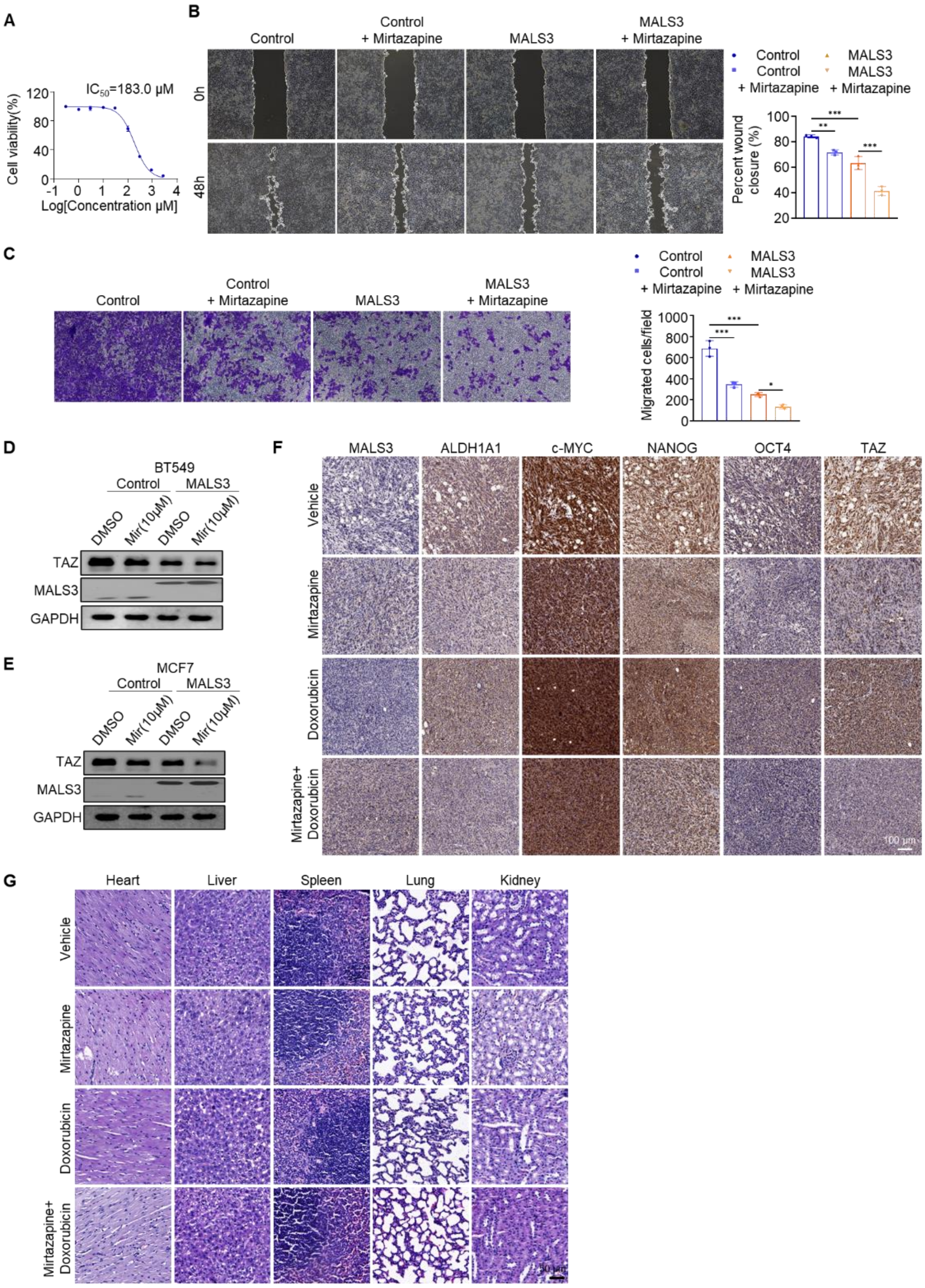

Fig. S6 (A) Dose-response curve of mirtazapine in 4T1 breast cancer cells. **(B)** Wound-healing assay in 4T1 cells (control and MALS3-overexpressing) treated with mirtazapine. **(C)** Transwell assay in 4T1 cells (control and MALS3-overexpressing) treated with mirtazapine. **(D** and **E)** Western Blotting analysis of proteins collected from BT549 and MCF7 cells (control and MALS3-overexpressing) treated with mirtazapine. **(F)** Representative images from immunohistochemical staining for MALS3, ALDH1A1, c-MYC, NANOG, OCT4, and TAZ in tumor tissues from different mouse groups. Scale bar: 100 µm. **(G)** HE staining results of heart, liver, spleen, lung and kidney tissues in each group of mice. Scale bar: 50 µm. The drug concentrations used in the in vivo experiments were mirtazapine (10mg/kg), doxorubicin (1mg/kg). All data are indicated as mean ± SD. Abbreviation: **P*<0.05, ***P*<0.01, ****P*<0.001. Statistical comparisons were performed using by one-way ANOVA (**B** and **C**).
