# Supplementary material for: Polarity protein MALS3 suppresses breast cancer stem cell-like properties by promoting TAZ inactivation and ubiquitination-dependent degradation
Source: Cell Death Dis. 2026 Jun 21;17(1):947. doi: 10.1038/s41419-026-09010-7 (PMC13743759; doi:10.1038/s41419-026-09010-7)
Supplement: Supplementary file 2 [file 41419_2026_9010_MOESM2_ESM.pdf]

Table S1. Detailed information of sgRNA sequences used in the experiments.

sgRNA	KO#1 sgRNA Sequence
sgRNA1	GCTAGGGGAACCCGTGCGGC
sgRNA2	AAAGCCTGAAGTTTCTGTGG

sgRNA	KO#2 sgRNA Sequence
sgRNA3	CGGCGCTAGGGGAACCCGTG
sgRNA4	TGAACATGTCTATGAGACTG

Table S2. Proteins interacting with MALS3 identified by IP-MS.

No.	Gene names	No.	Gene names
1	PLEC	151	DSG2
2	ACTB	152	CLTC
3	ACTG1	153	SFXN1
4	VIM	154	ARF3
5	ACTA1	155	DDOST
6	POTEE	156	CAD
7	ACTBL2	157	NTPCR
8	FSCN1	158	RPL28
9	DLG1	159	ITIH2
10	LAD1	160	MGST1
11	LIMA1	161	MAP4

12	TUBB	162	RPL22
13	DSP	163	RPS14
14	FAM83H	164	ELAVL1
15	CASK	165	ILF2
16	TUBB4B	166	RPLP0
17	FLNB	167	PPP1CA
18	TUBA1B	168	RPS23
19	MPRIP	169	RPN1
20	PKP3	170	RPS15A
21	PKP2	171	RPS8
22	FLNA	172	NCL
23	LIN7C	173	SERPINC1
24	SVIL	174	HNRNPCL1
25	PALS2	175	PKP4
26	EEF1A1	176	RPL13A
27	HSPA9	177	RPS11
28	SPTAN1	178	ABLIM3
29	ACTN4	179	RPL10L
30	TUBB6	180	APOB
31	SFPQ	181	MT-CO2
32	GLB1	182	HSPD1
33	PALS1	183	ILF3

34	HSPA8	184	ATP5PD
35	SPTBN1	185	PATJ
36	MYO1C	186	ATP5MF
37	ATP5F1A	187	RPLP2
38	MYH9	188	UQCRC2
39	ACTN1	189	PFKP
40	CORO1C	190	YWHAZ
41	MYO1B	191	RPLP1
42	EFHD2	192	CSNK1D
43	CAPZA1	193	DLST
44	HNRNPM	194	RPL27A
45	ALDH1A3	195	RPL35A
46	GRN	196	SLC7A5
47	GAN	197	CRYBG1
48	SLC25A5	198	MYL6
49	RPS3	199	KIAA1671
50	HNRNPA1	200	RPS25
51	LCP1	201	RPS27L
52	CALM1	202	PSMD3
53	RBM14	203	RPL8
54	ANXA2	204	RPL31
55	DBN1	205	SLC25A13

56	ITGB4	206	RPL29
57	CAPZB	207	DLG4
58	MYO1D	208	RPL4
59	HSPB1	209	ITIH1
60	CSNK1A1	210	AP3B1
61	PYGB	211	CALD1
62	CAPZA2	212	PGAM5
63	ALB	213	RPL23
64	C3	214	CANX
65	PPP1R18	215	MATR3
66	HNRNPH1	216	PPP1R12A
67	MPP7	217	MLF2
68	TRIM29	218	DHCR7
69	H4C16	219	LAMA3
70	HNRNPU	220	RPL38
71	SLC25A3	221	RALGAPA2
72	C4A	222	NUDT21
73	CORO2A	223	MYO3B
74	TRIM27	224	LRRC59
75	FAM83B	225	RPL12
76	ATP5PO	226	RPL27
77	ATP5F1B	227	CNTNAP4

78	RPL13	228	SNRPB
79	HNRNPF	229	ITIH4
80	SLC25A6	230	HSD17B12
81	SLC3A2	231	POLDIP3
82	ATAD3A	232	S100A14
83	NPM1	233	IMMT
84	ATAD3B	234	RPL6
85	WWTR1	235	NDUFA13
86	MISP	236	TWF1
87	JUP	237	HNRNPA0
88	RPS18	238	OCIAD2
89	YAP1	239	KHDRBS1
90	MYO5A	240	RPL17
91	RPS5	241	DPM1
92	DDX3X	242	LDHC
93	DDX5	243	VDAC1
94	ARF4	244	ATP5MG
95	RPS10	245	SNTB2
96	LAMB3	246	SLC16A3
97	RPS13	247	TMED10
98	PCM1	248	SEC61A1
99	YBX1	249	IGLV3-9

100	RPS7	250	CAV1
101	HNRNPA2B1	251	FANCI
102	CTSA	252	KBTBD3
103	IGKV2-29	253	ABCA13
104	TPM3	254	H2BC21
105	RCN2	255	XPNPEP3
106	SERPIND1	256	PHB1
107	RPS16	257	GNA12
108	IQGAP1	258	PGD
109	FUS	259	RPS26
110	OAT	260	THOC7
111	RPS20	261	DYNLL2
112	RPL23A	262	RPS6
113	HSP90AB1	263	KIAA1217
114	HSPA5	264	LCMT1
115	ATP5F1C	265	NDUFA10
116	PSMC5	266	PPP2CB
117	PLS3	267	NDUFS8
118	LATS1	268	RRBP1
119	NONO	269	PSPC1
120	DDX17	270	TGFBI
121	PTBP1	271	PTPN14

122	RPS2	272	DNAJC11
123	MRPL14	273	CHRAC1
124	PABPC1	274	RPS29
125	ARHGEF2	275	PLEKHA6
126	H2AC20	276	NDUFS7
127	LGALS3BP	277	RPL19
128	UBA52	278	HRNR
129	HNRNPK	279	MAPK7
130	LATS2	280	CORO1B
131	A2M	281	AGPAT1
132	TMOD3	282	ART4
133	APOA1	283	IGLV2-11
134	TUBA4B	284	LAMC2
135	SQOR	285	HNRNPD
136	EWSR1	286	MDH2
137	AGK	287	CPSF7
138	TMEM33	288	RPL22L1
139	DERPC	289	MAP1LC3B
140	LDHA	290	TIMM50
141	MYO5B	291	LBP
142	RPS19	292	RPS17
143	F2	293	SLC25A12

144	IGHG1	294	CCDC158
145	MPDZ	295	LSM4
146	COL17A1	296	PPCDC
147	GAPDH		
148	RPL37A		
149	TRAP1		
150	LMNA		
