# Supplementary material for: Polarity protein MALS3 suppresses breast cancer stem cell-like properties by promoting TAZ inactivation and ubiquitination-dependent degradation
Source: Cell Death Dis. 2026 Jun 21;17(1):947. doi: 10.1038/s41419-026-09010-7 (PMC13743759; doi:10.1038/s41419-026-09010-7)

Fig1E.

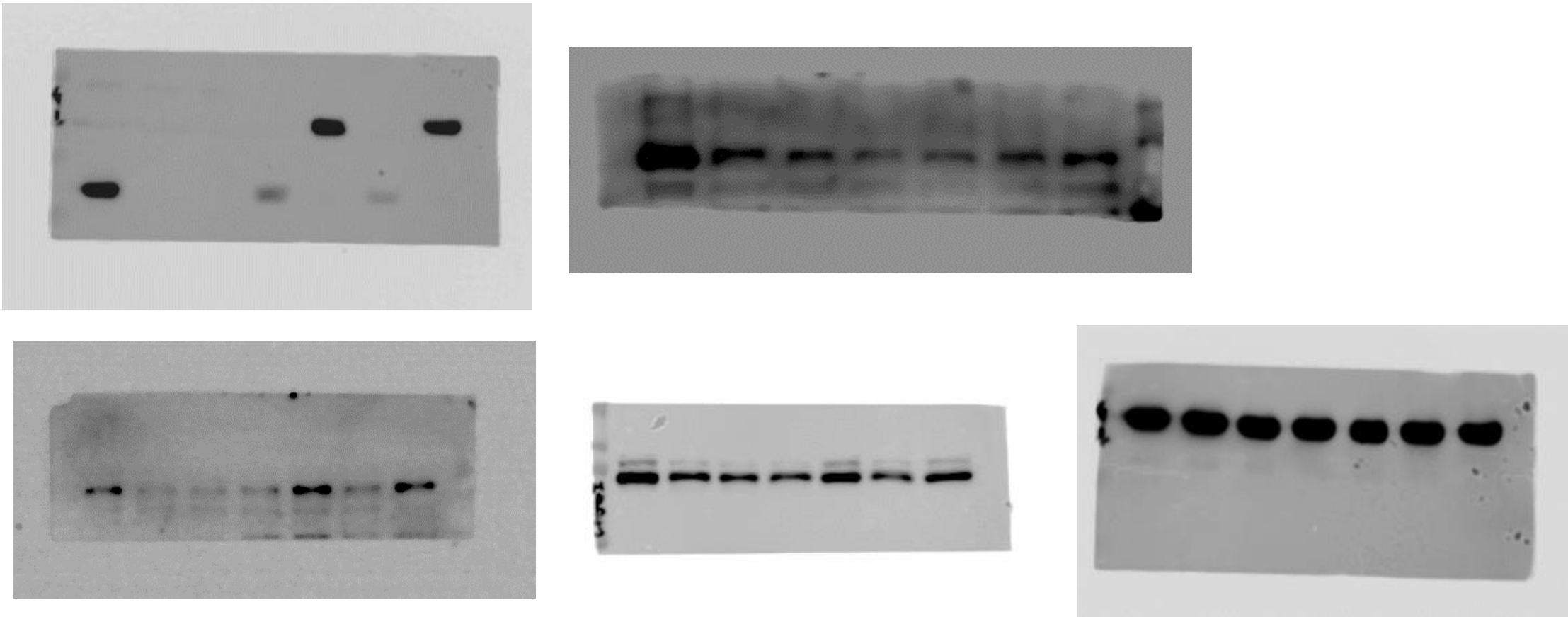

Fig2E.

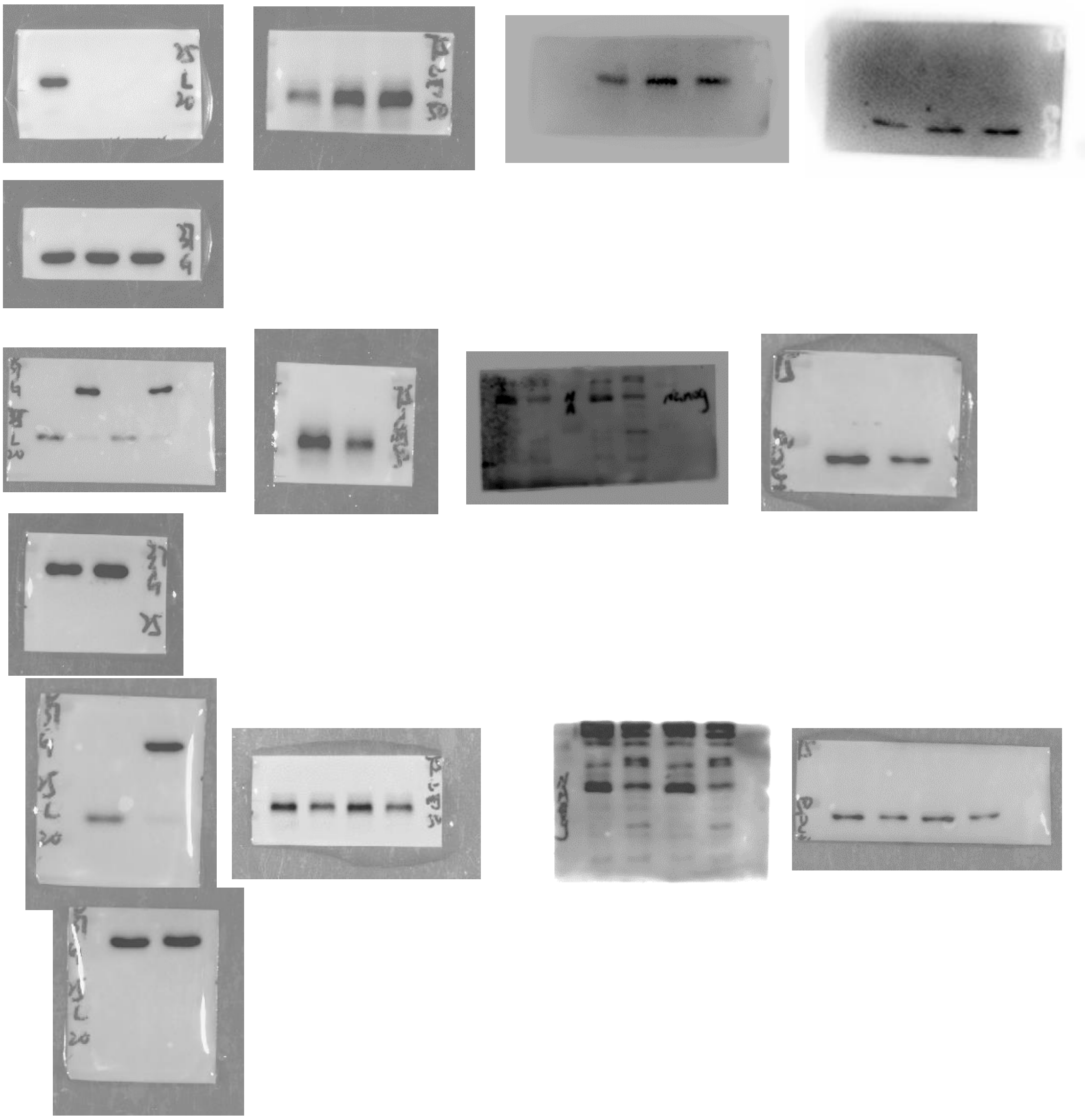

Fig3B.

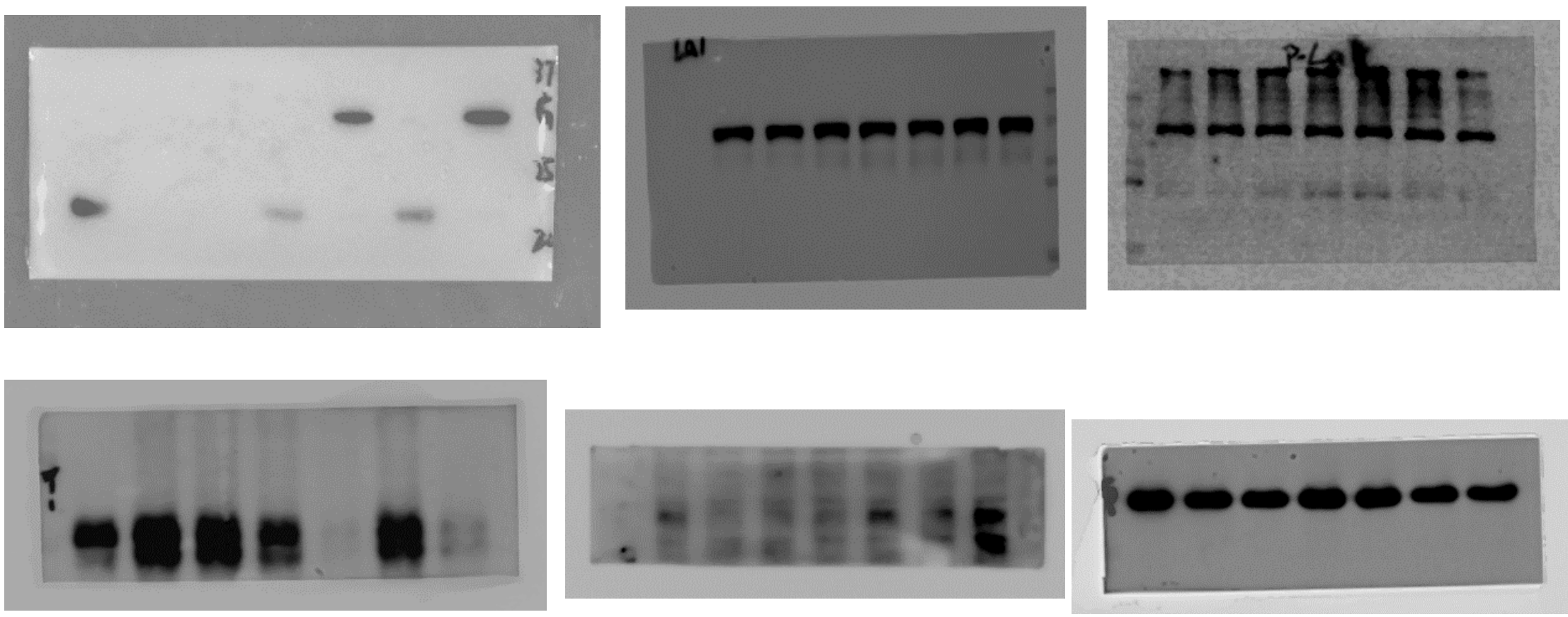

Fig3F.

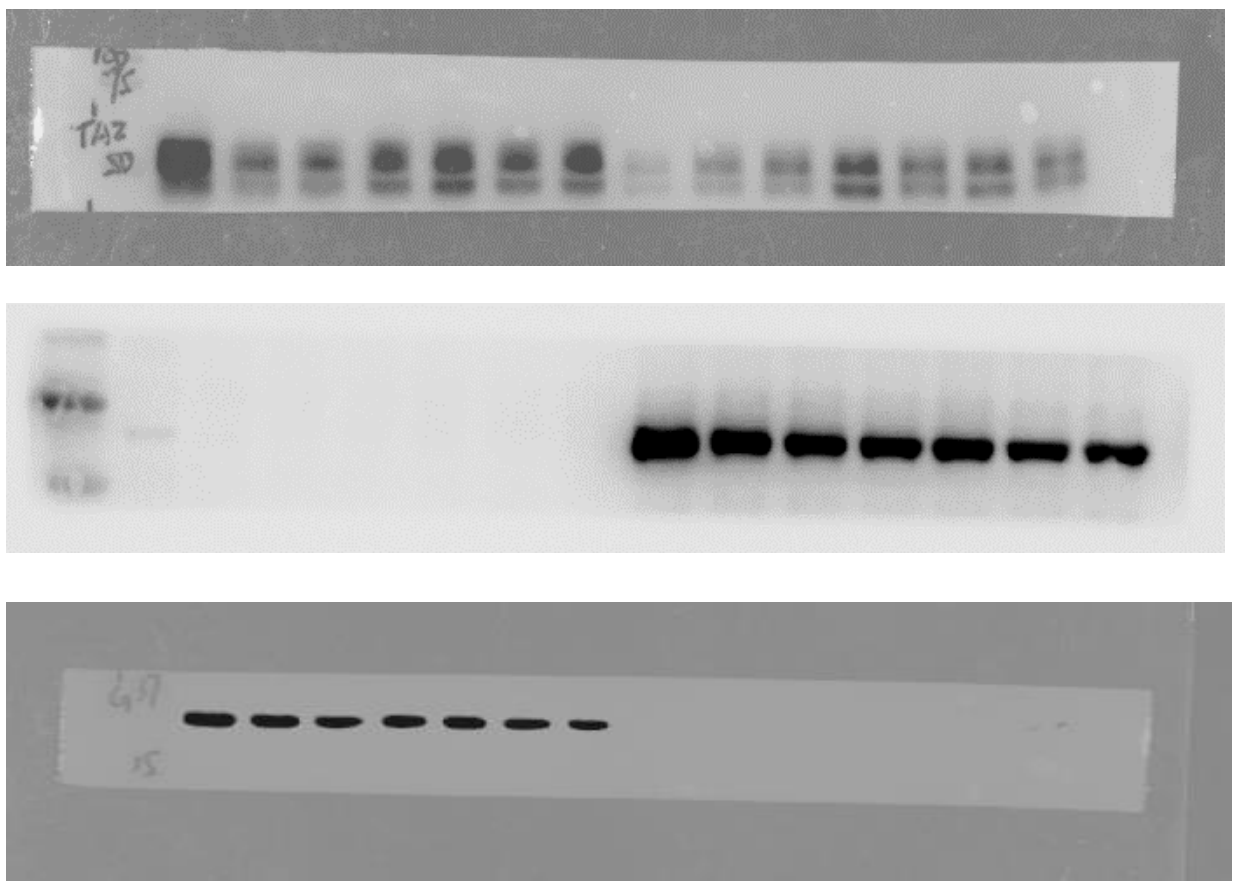

Fig3H.

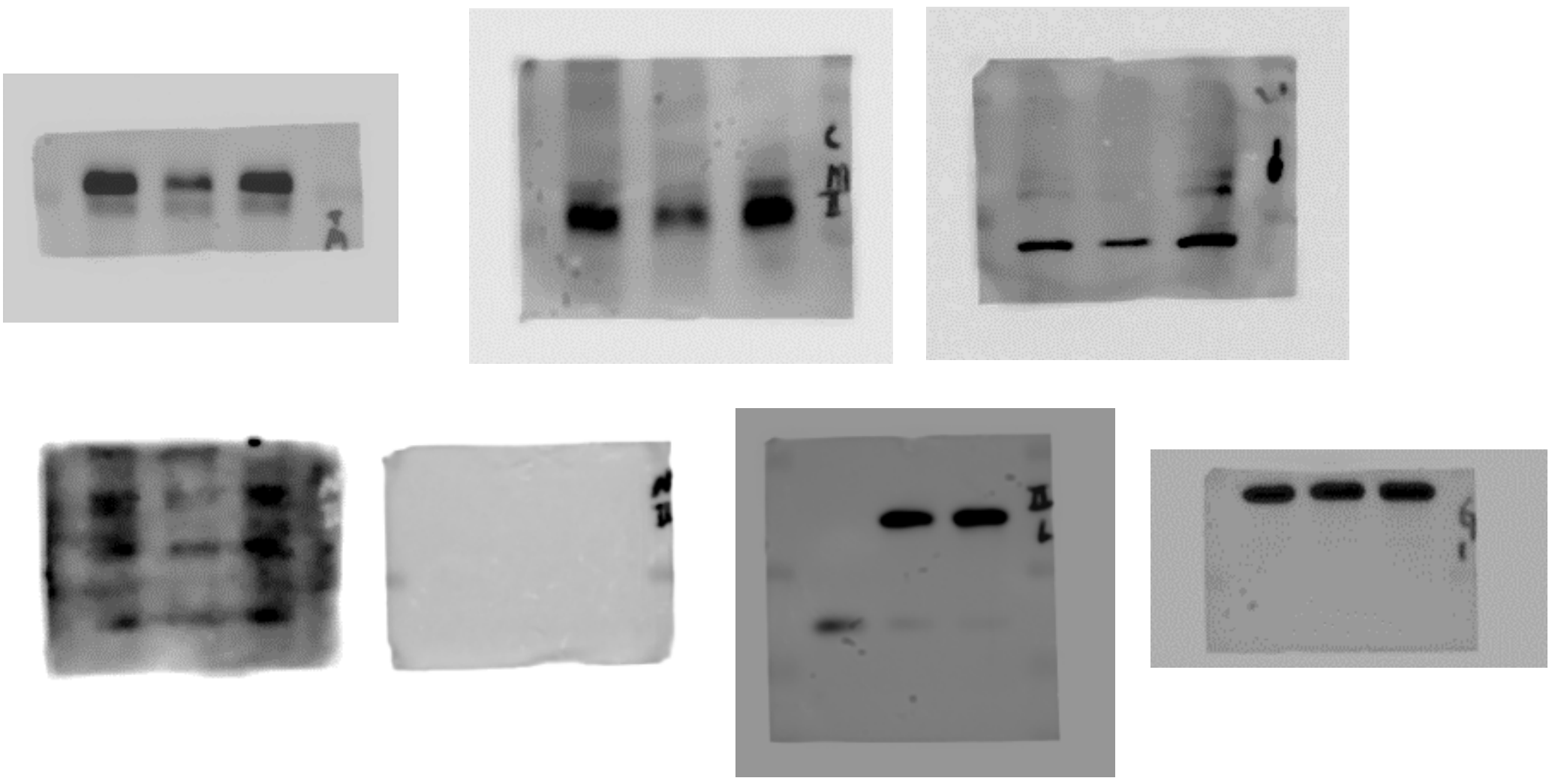

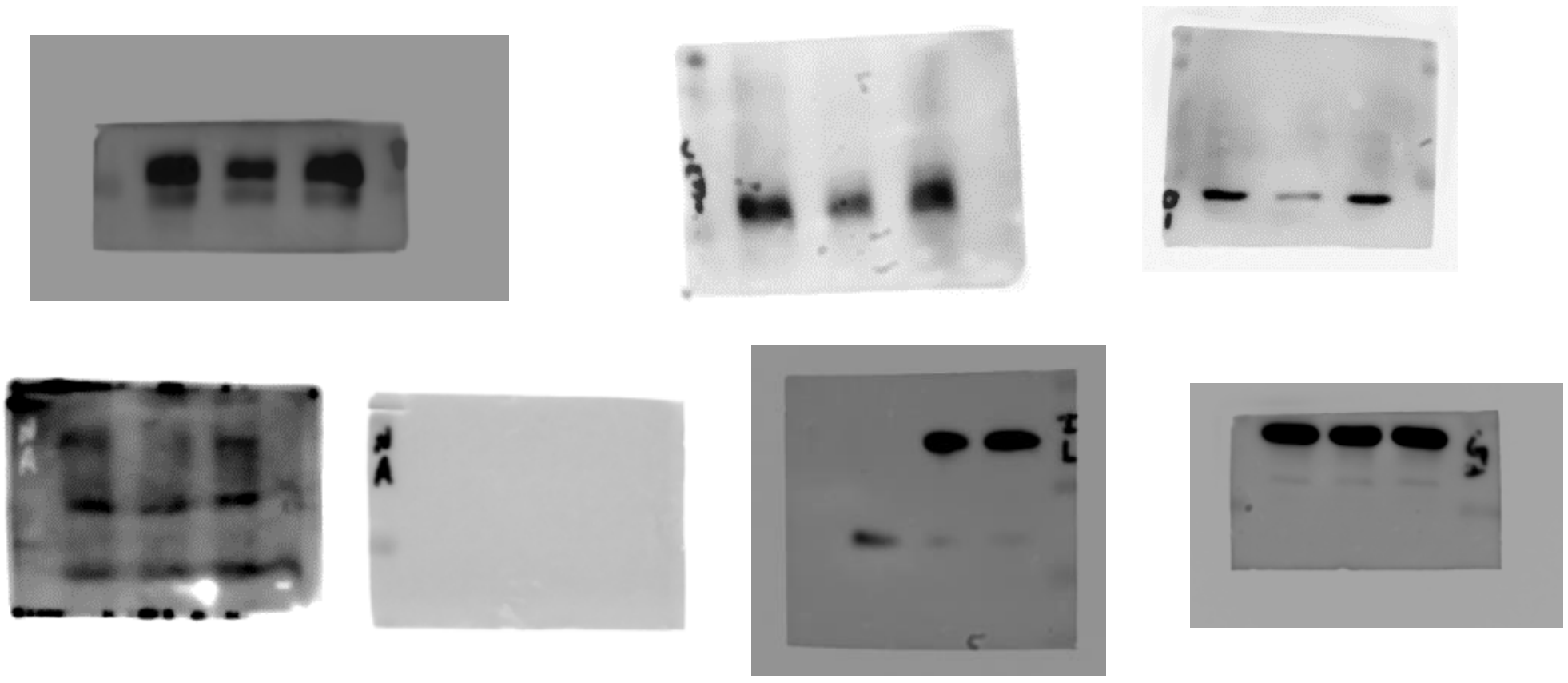

Fig4A.

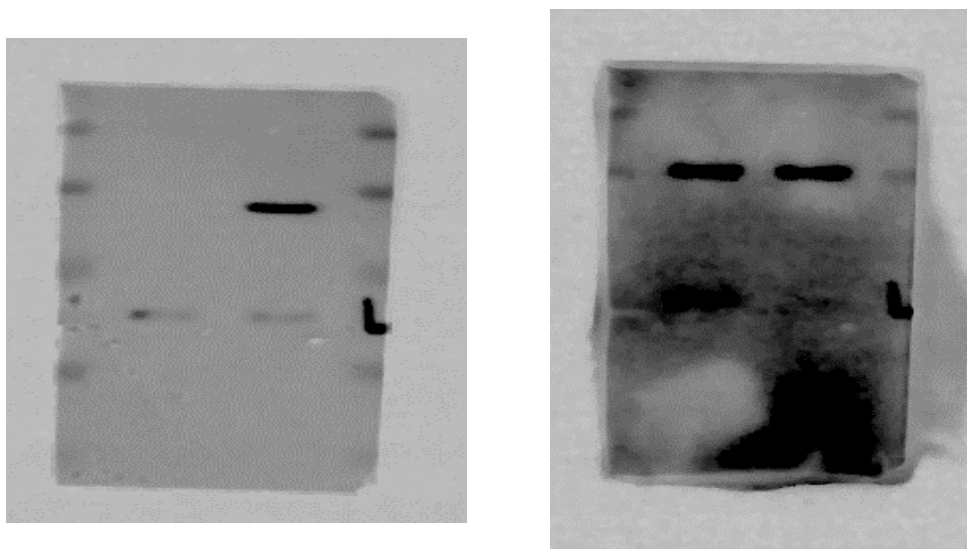

Fig5A.

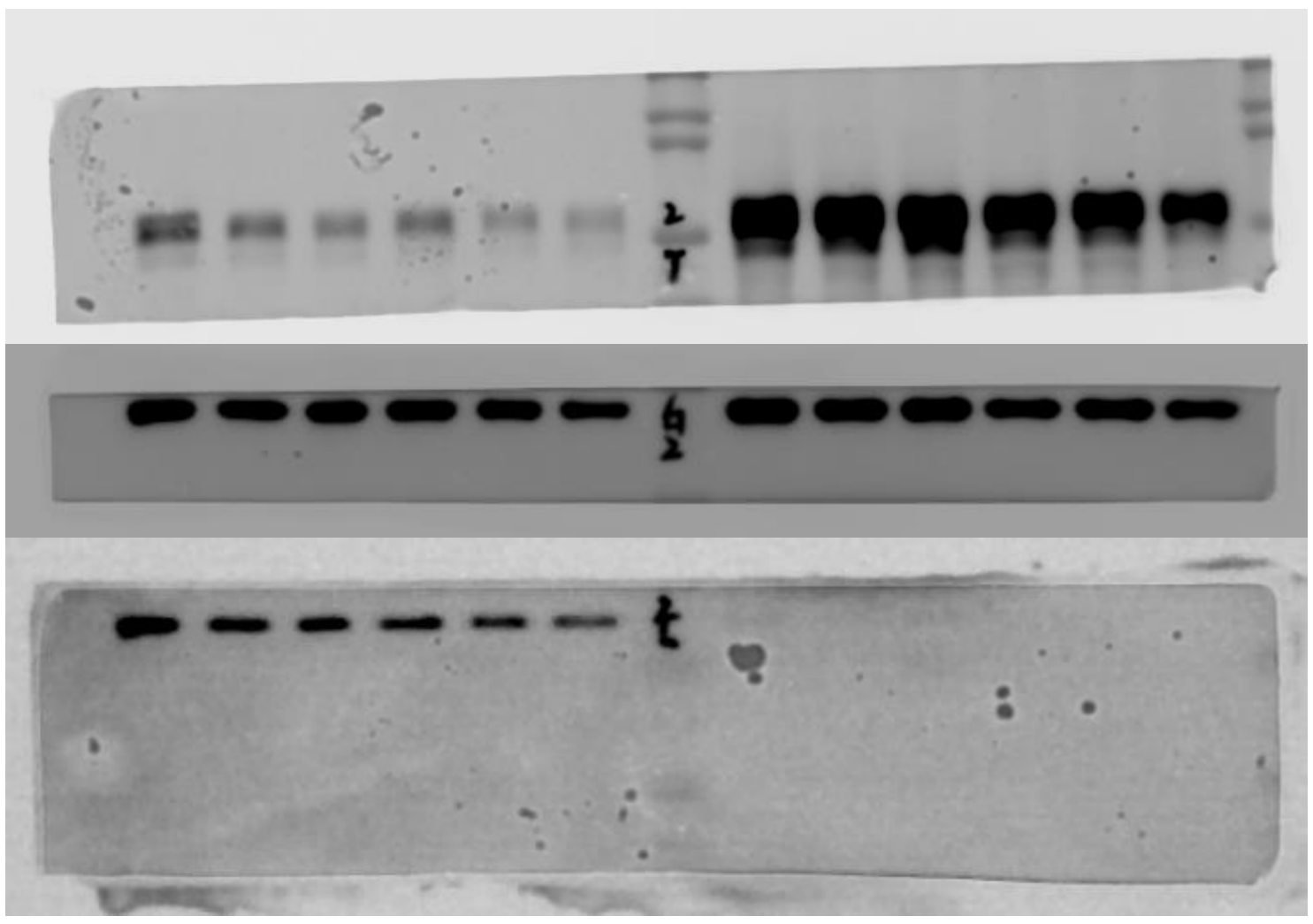

Fig5B.

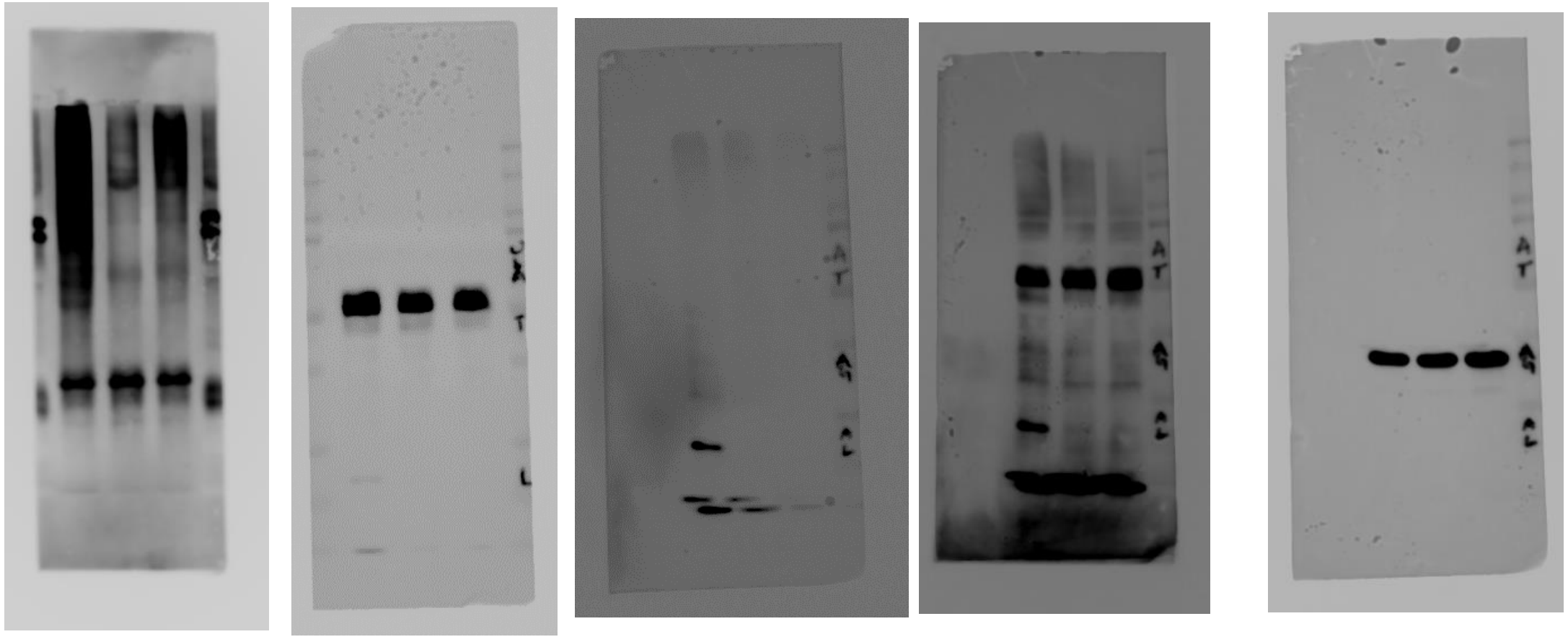

Fig5C.

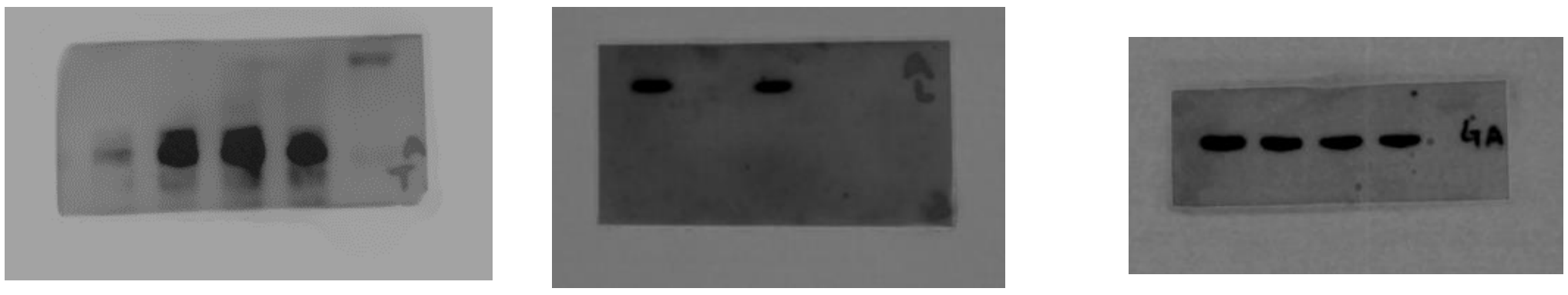

Fig5D.

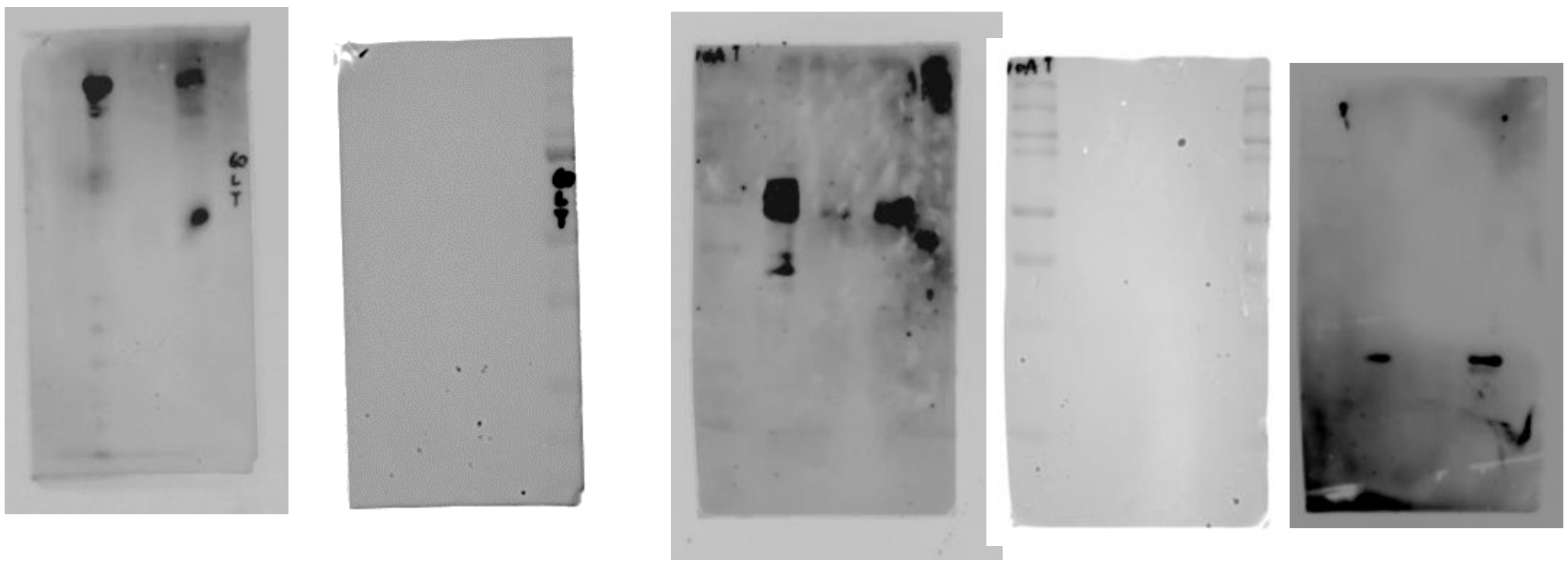

Fig5E.

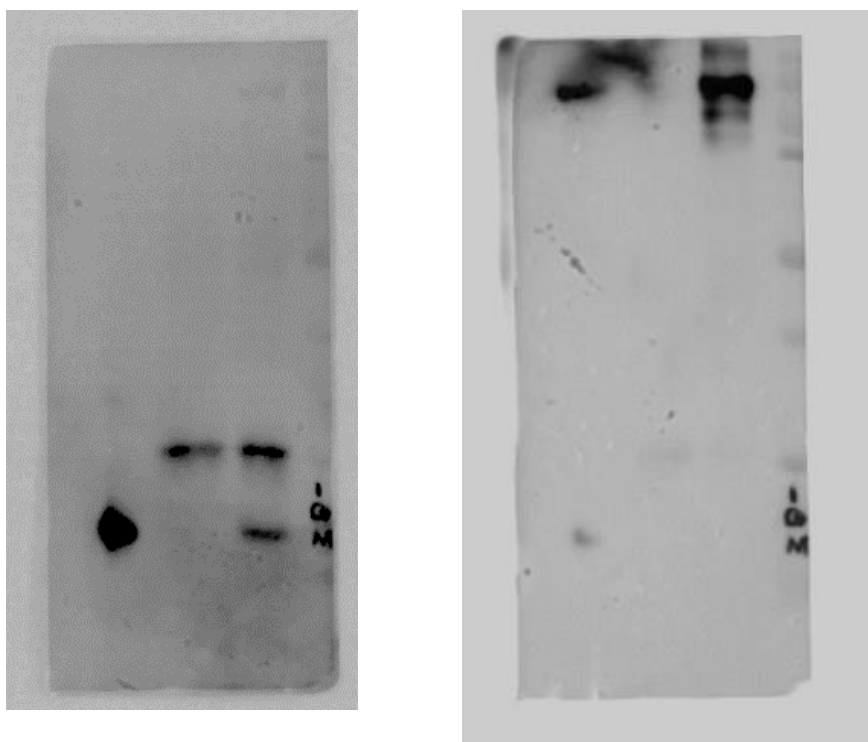

Fig5F.

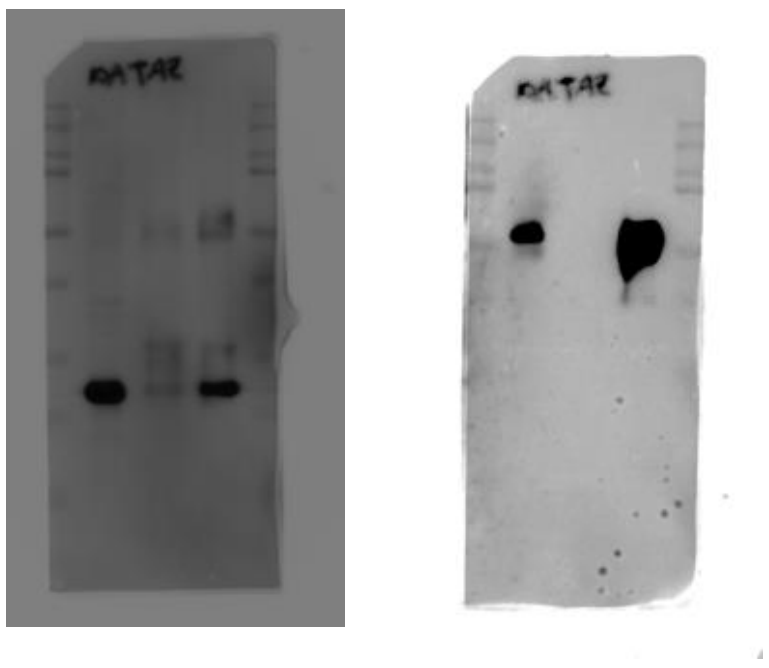

Fig5I.

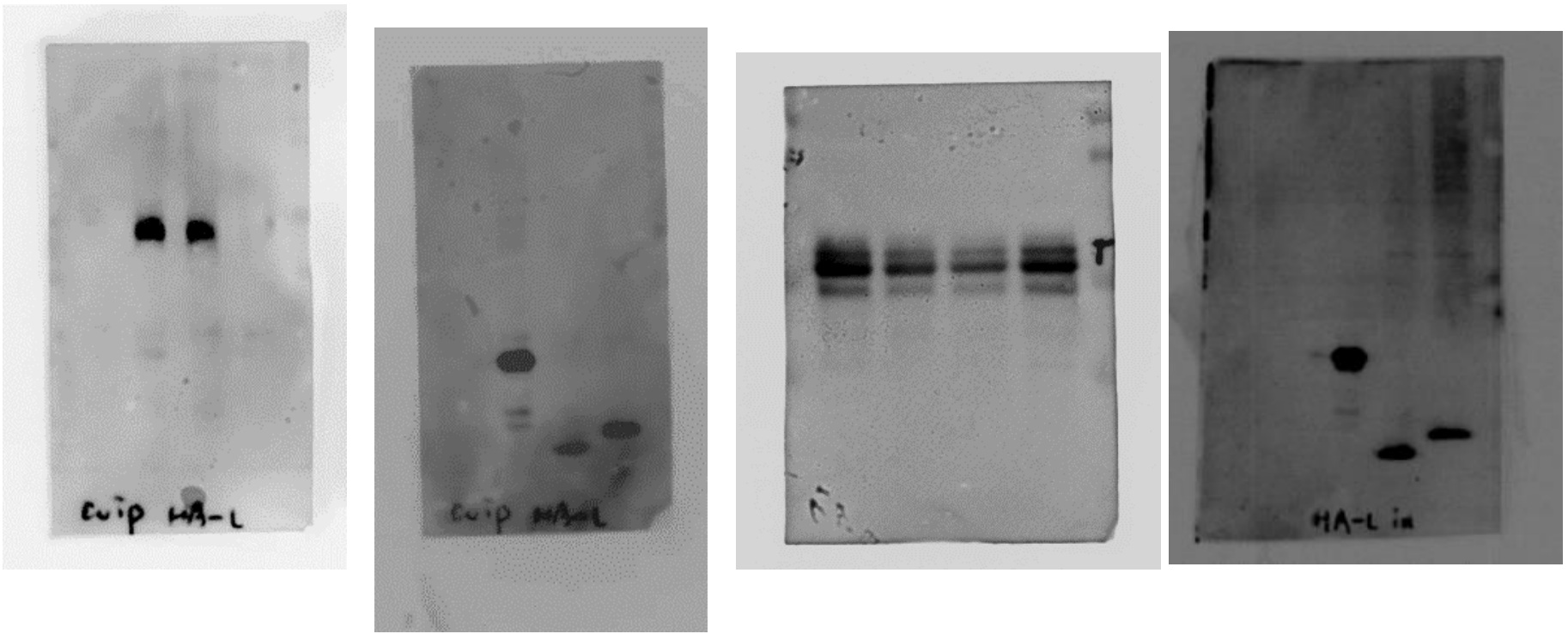

Fig5J.

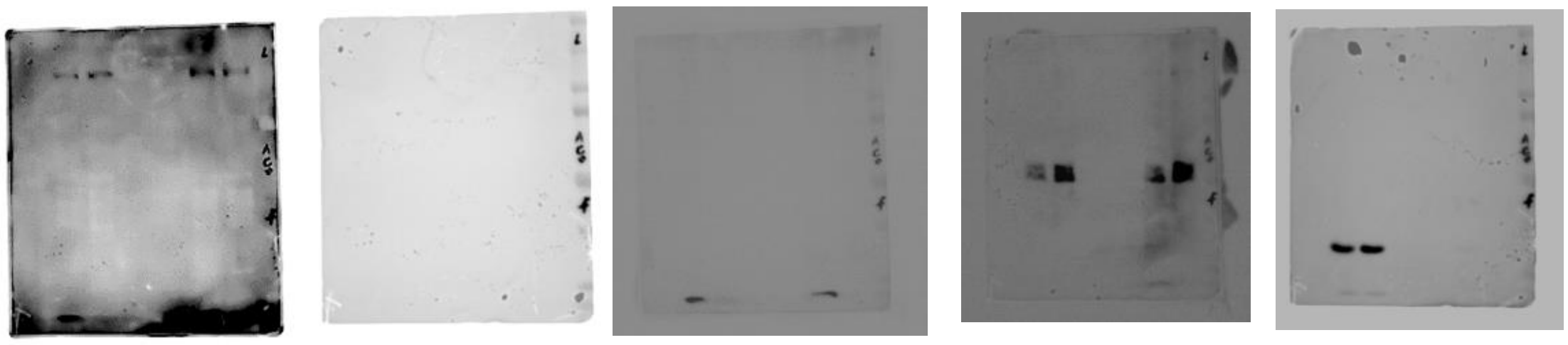

Fig5K.

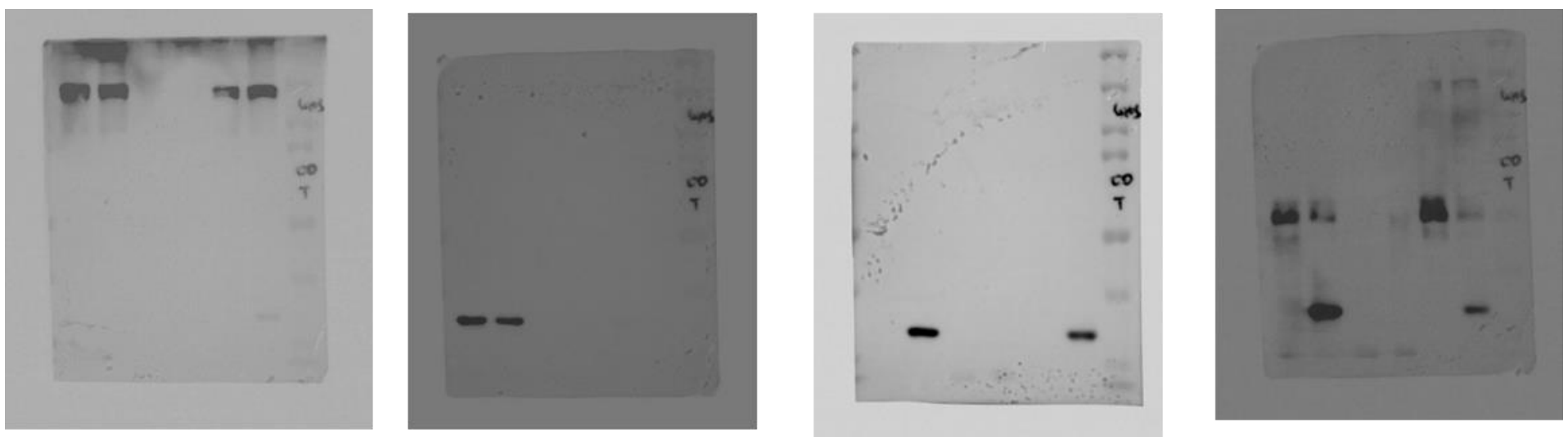

Fig5L.

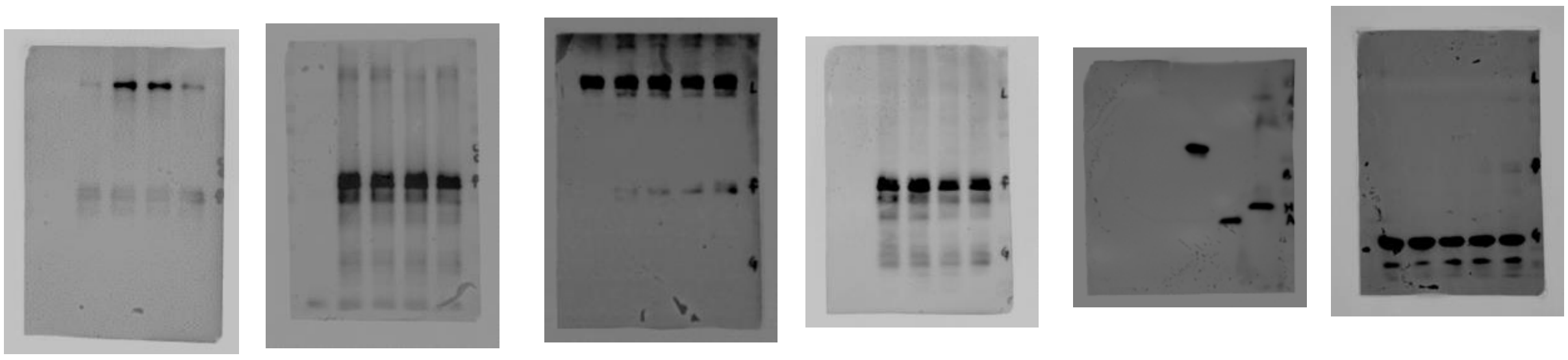

Fig5M.

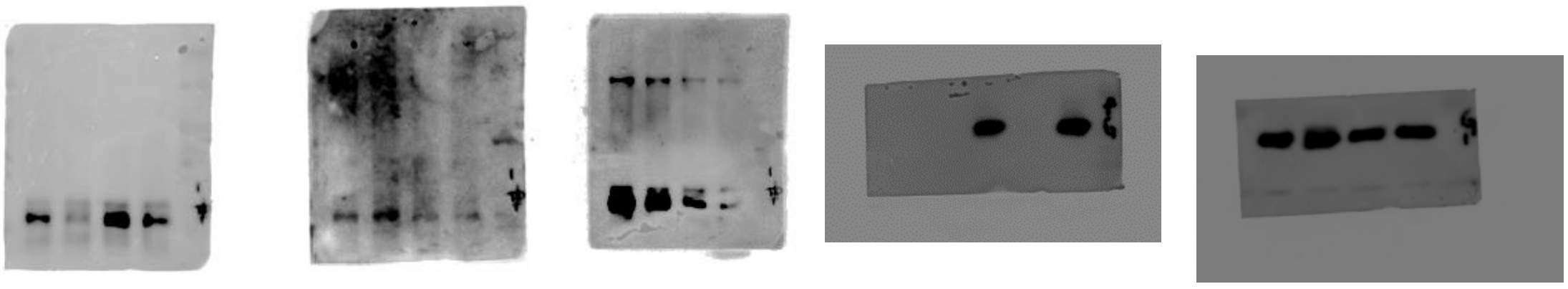

Fig5N.

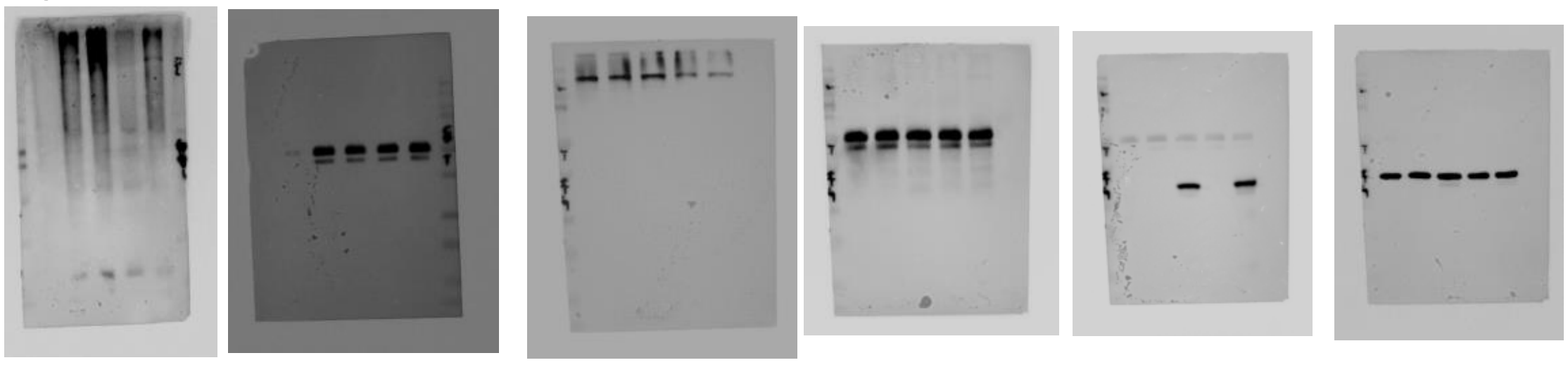

Fig6B.

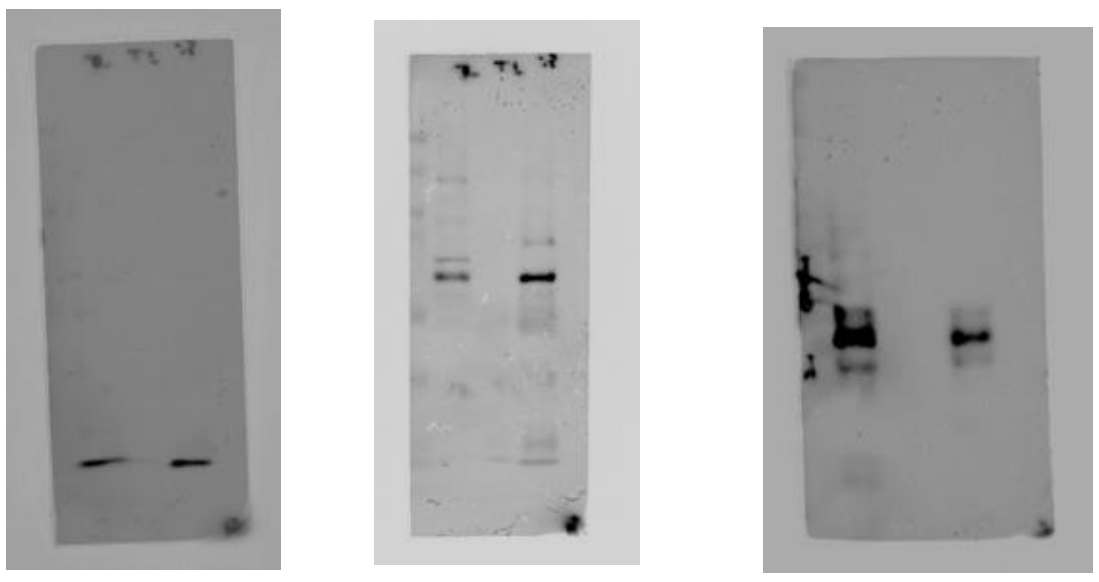

Fig6C.

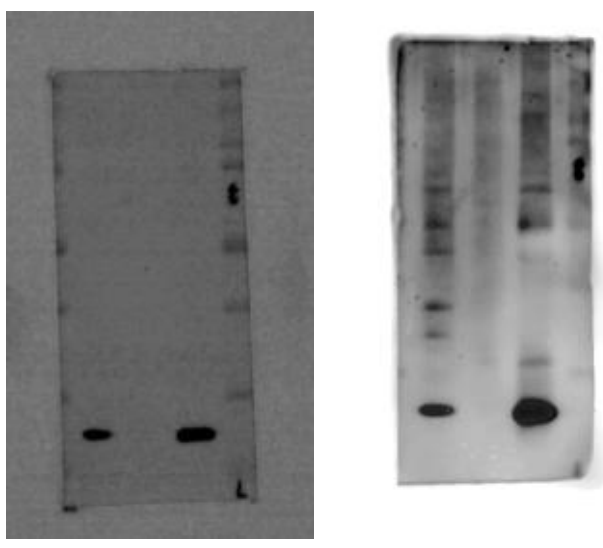

Fig6D.

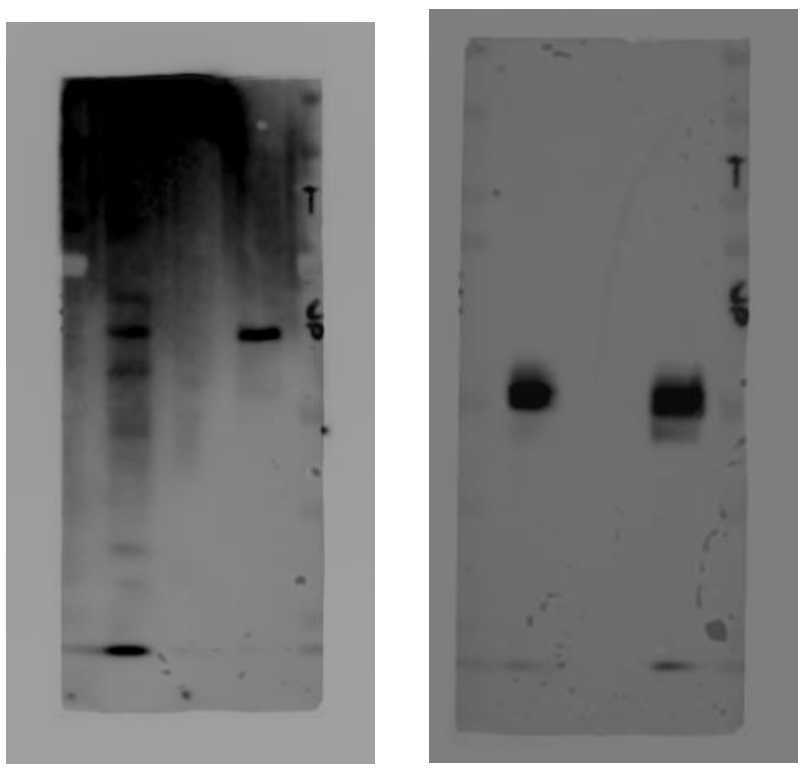

Fig6F.

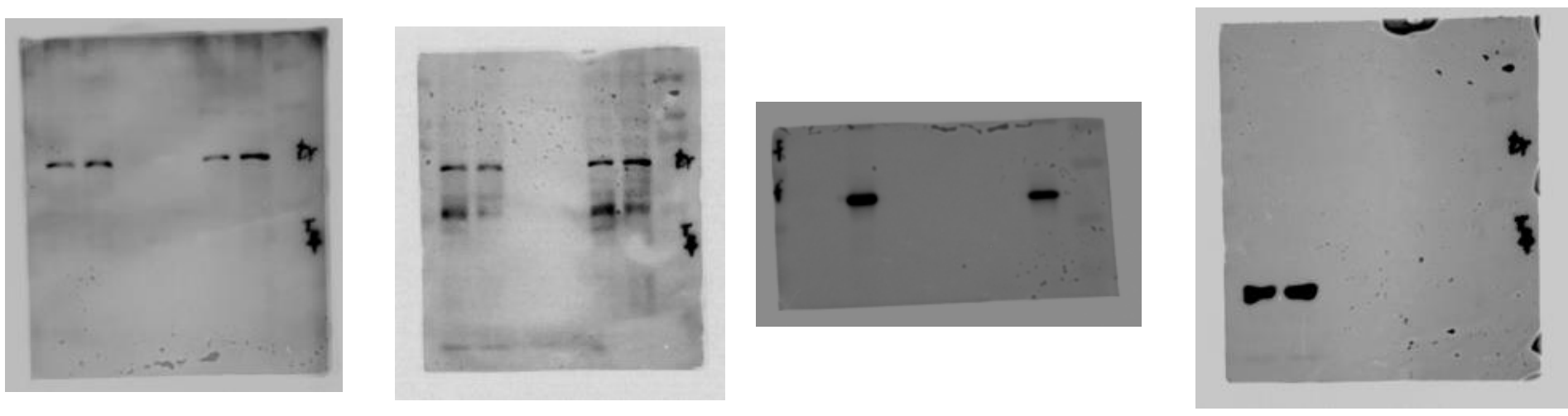

Fig6G.

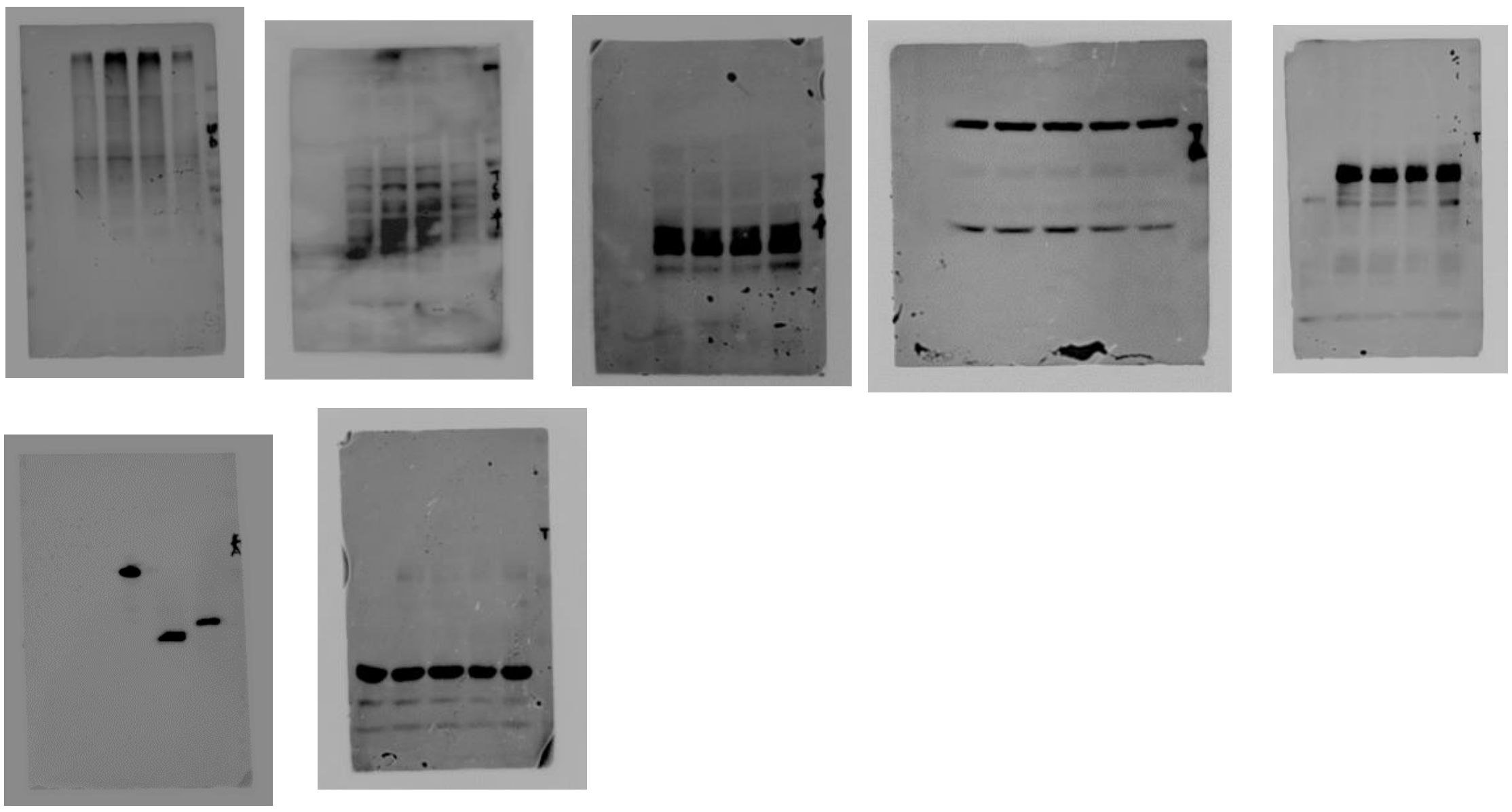

Fig6H.

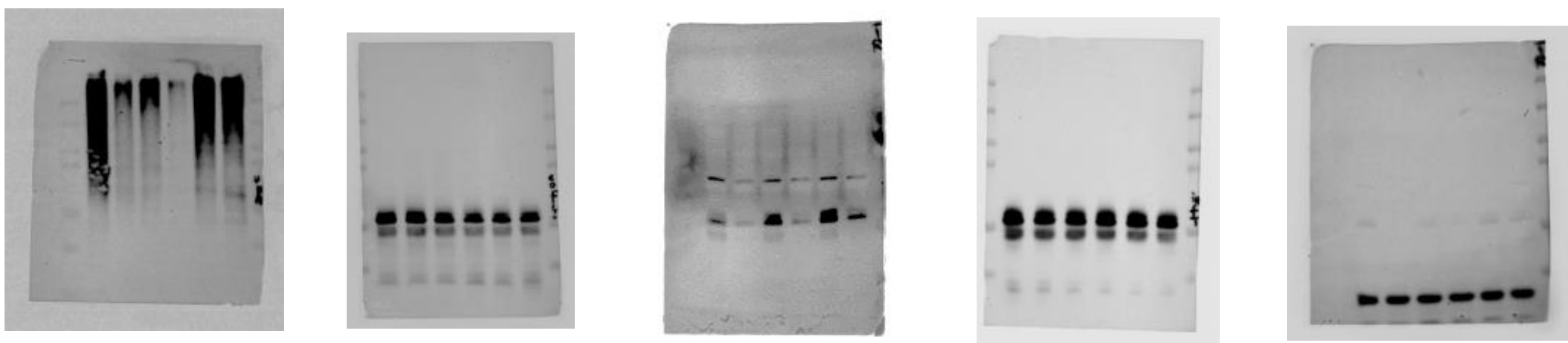

Fig6I.

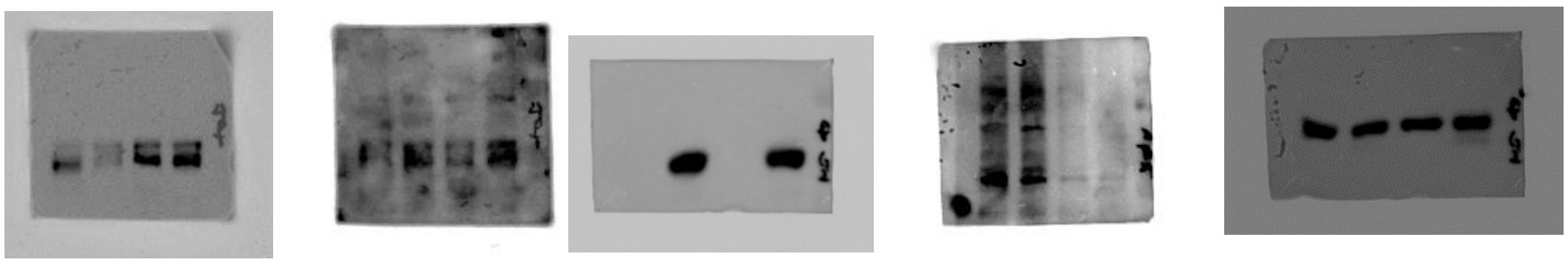

Fig6J.

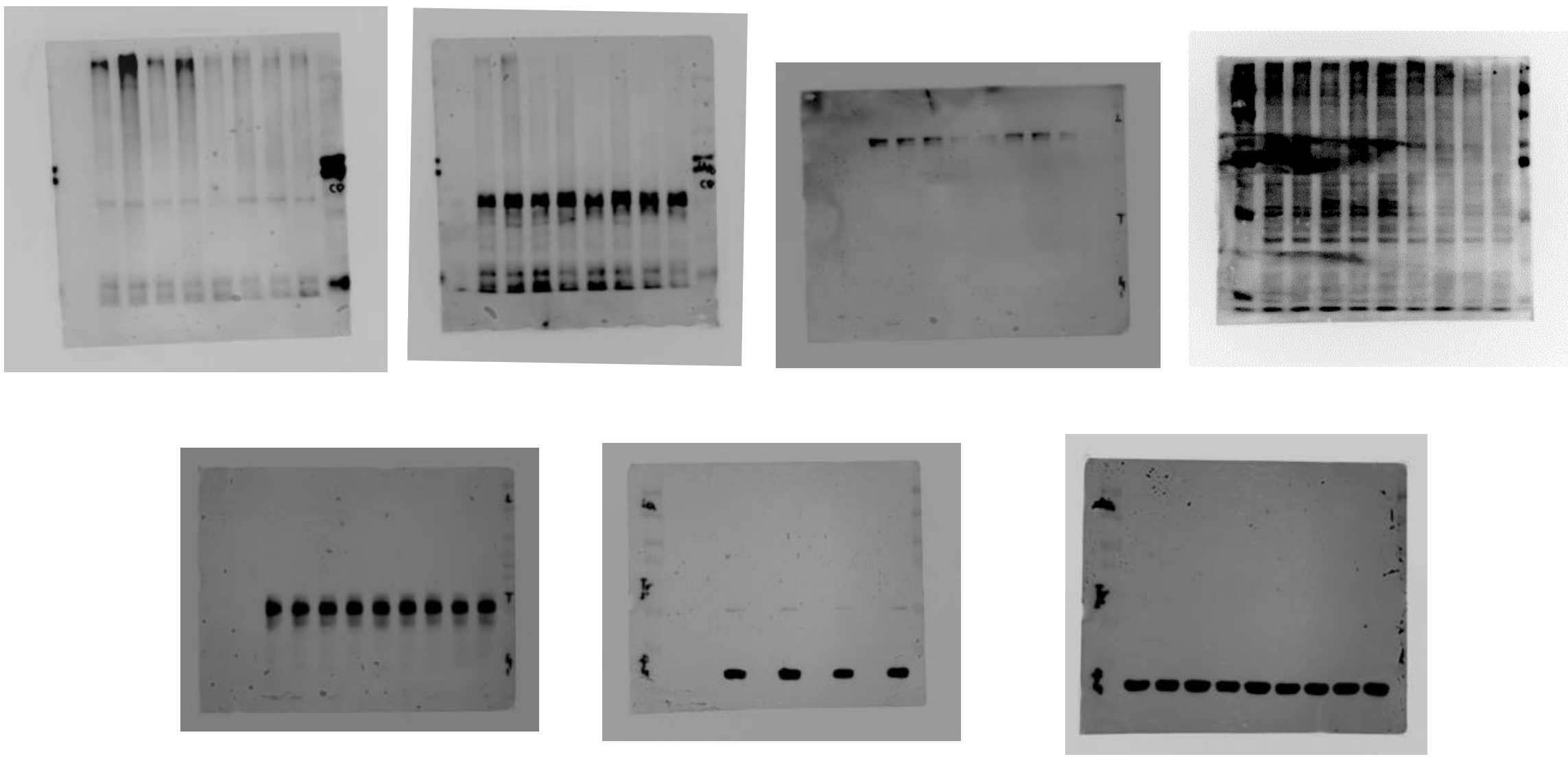

Fig7A.

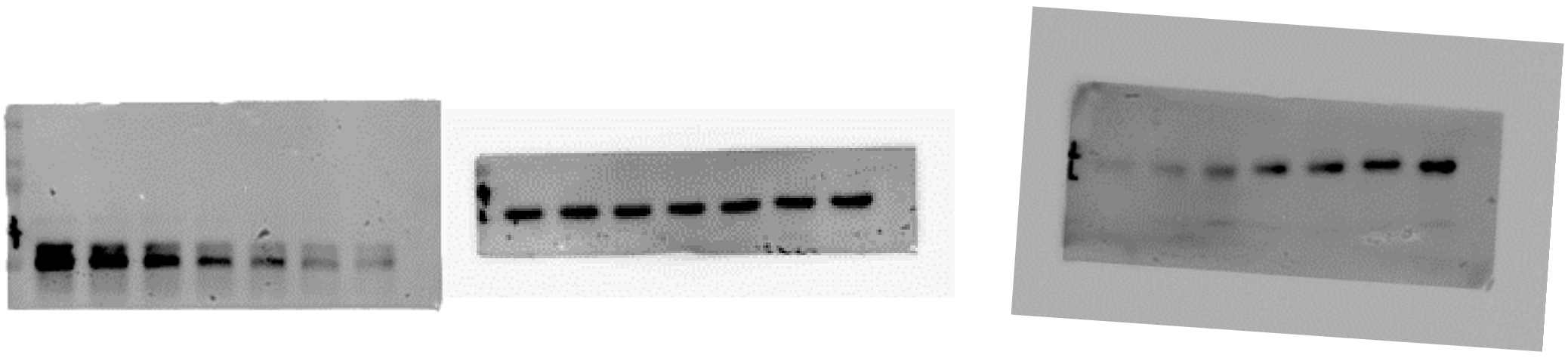

Fig7B.

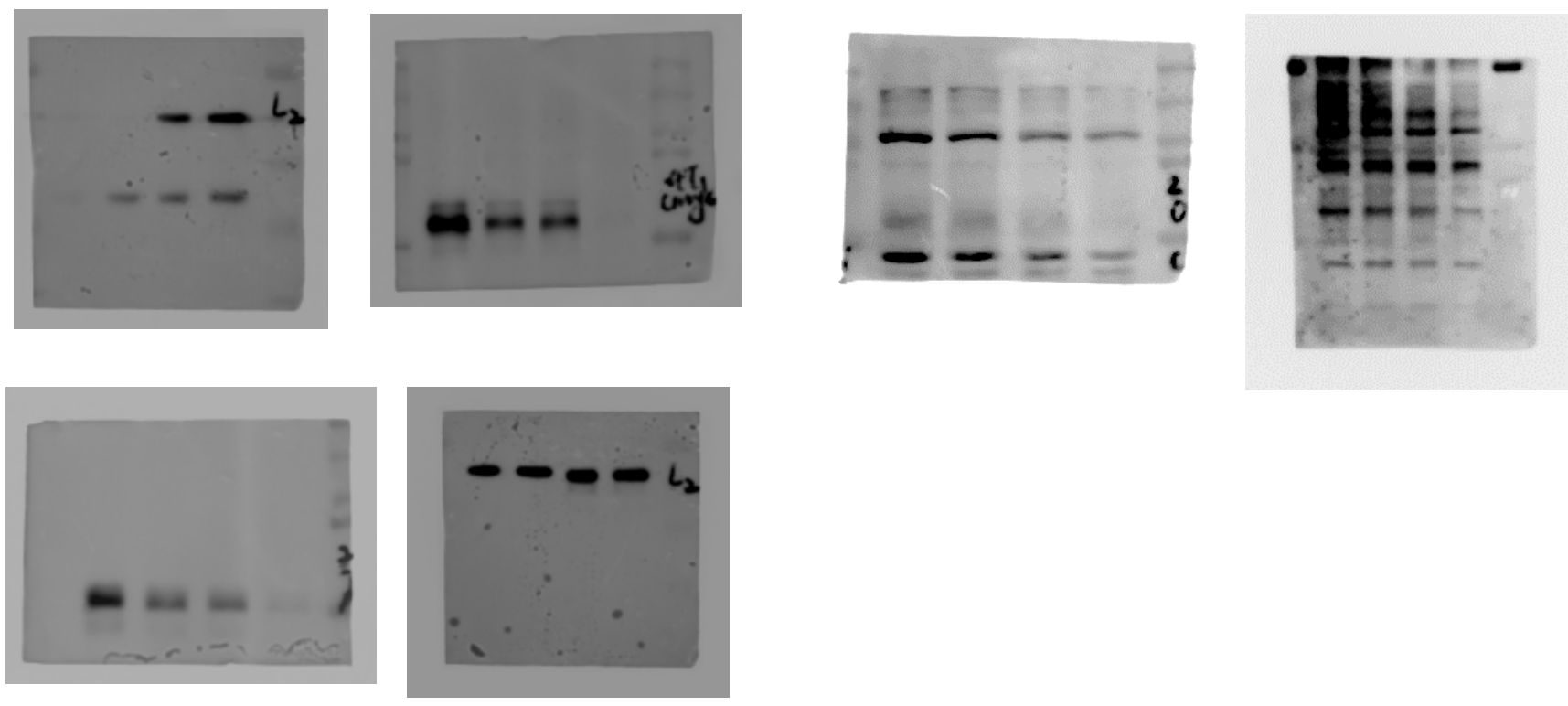

FigS1D.

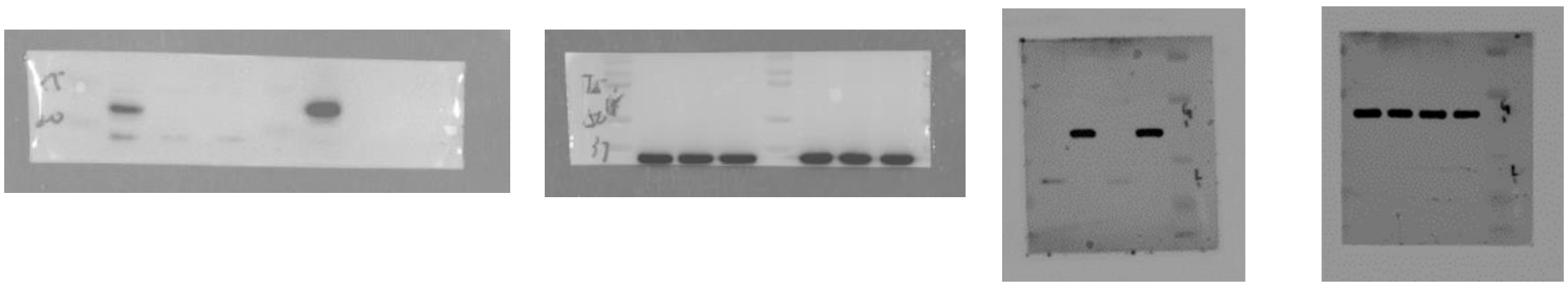

FigS3A.

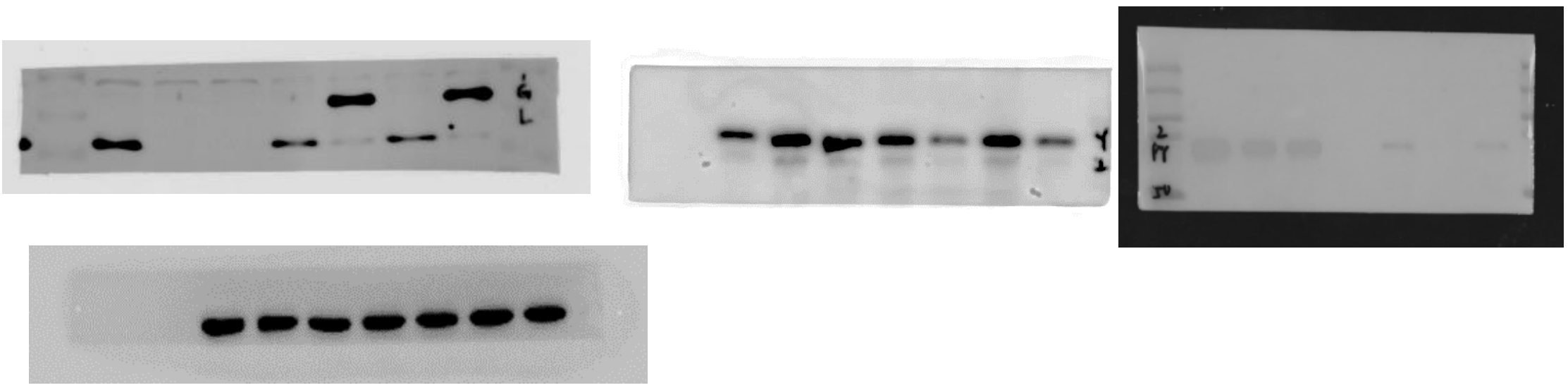

FigS3D.

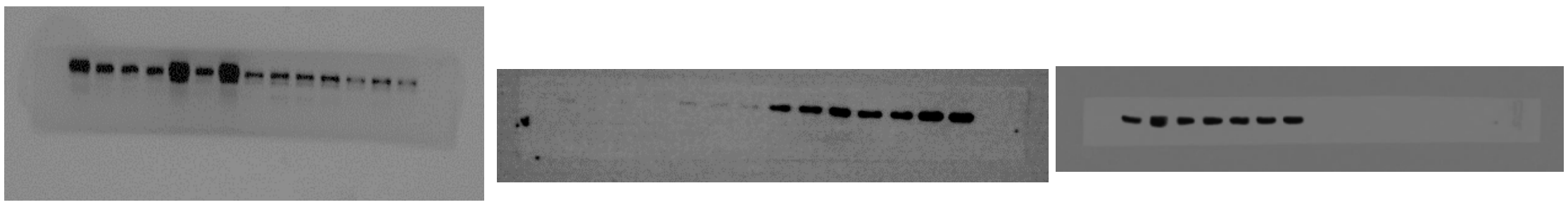

FigS4C.

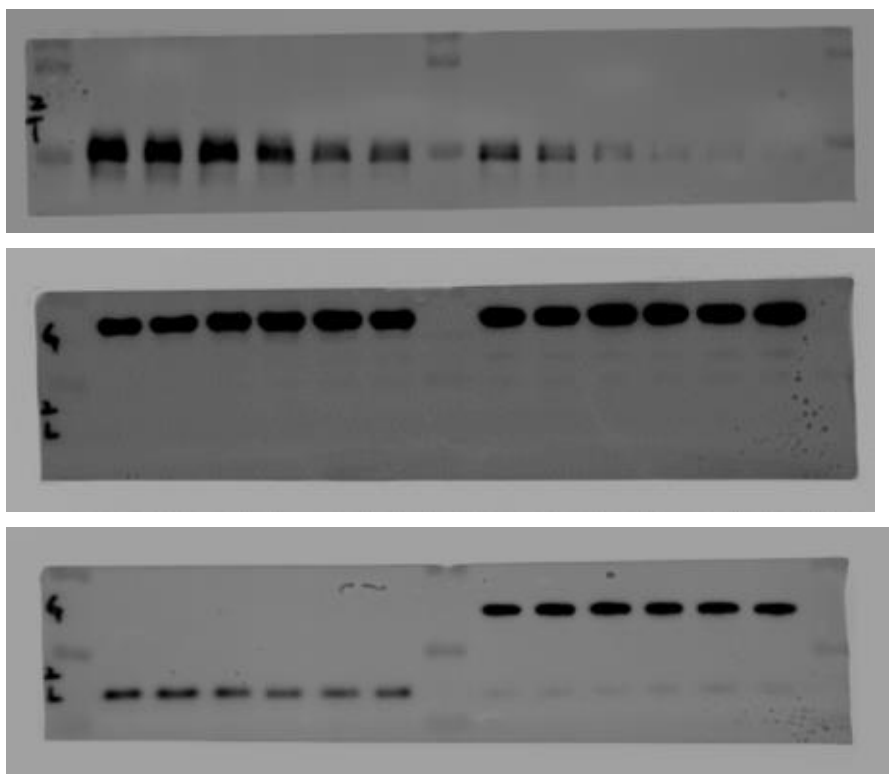

FigS4D.

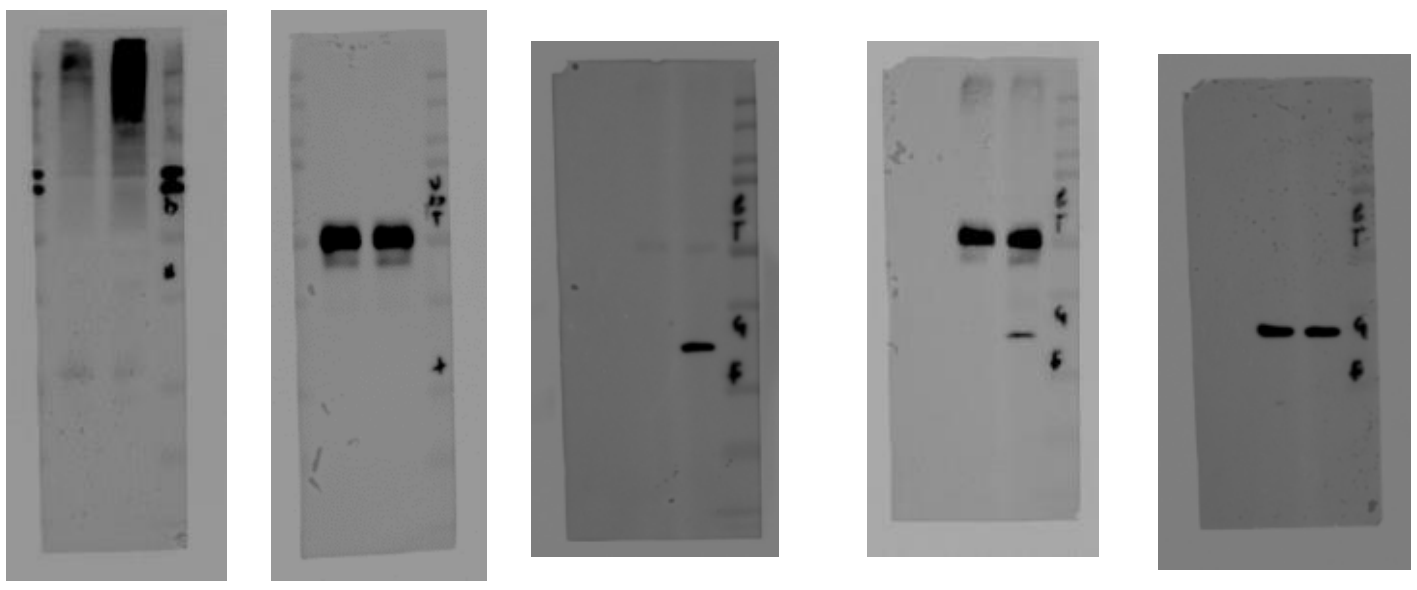

FigS4E.

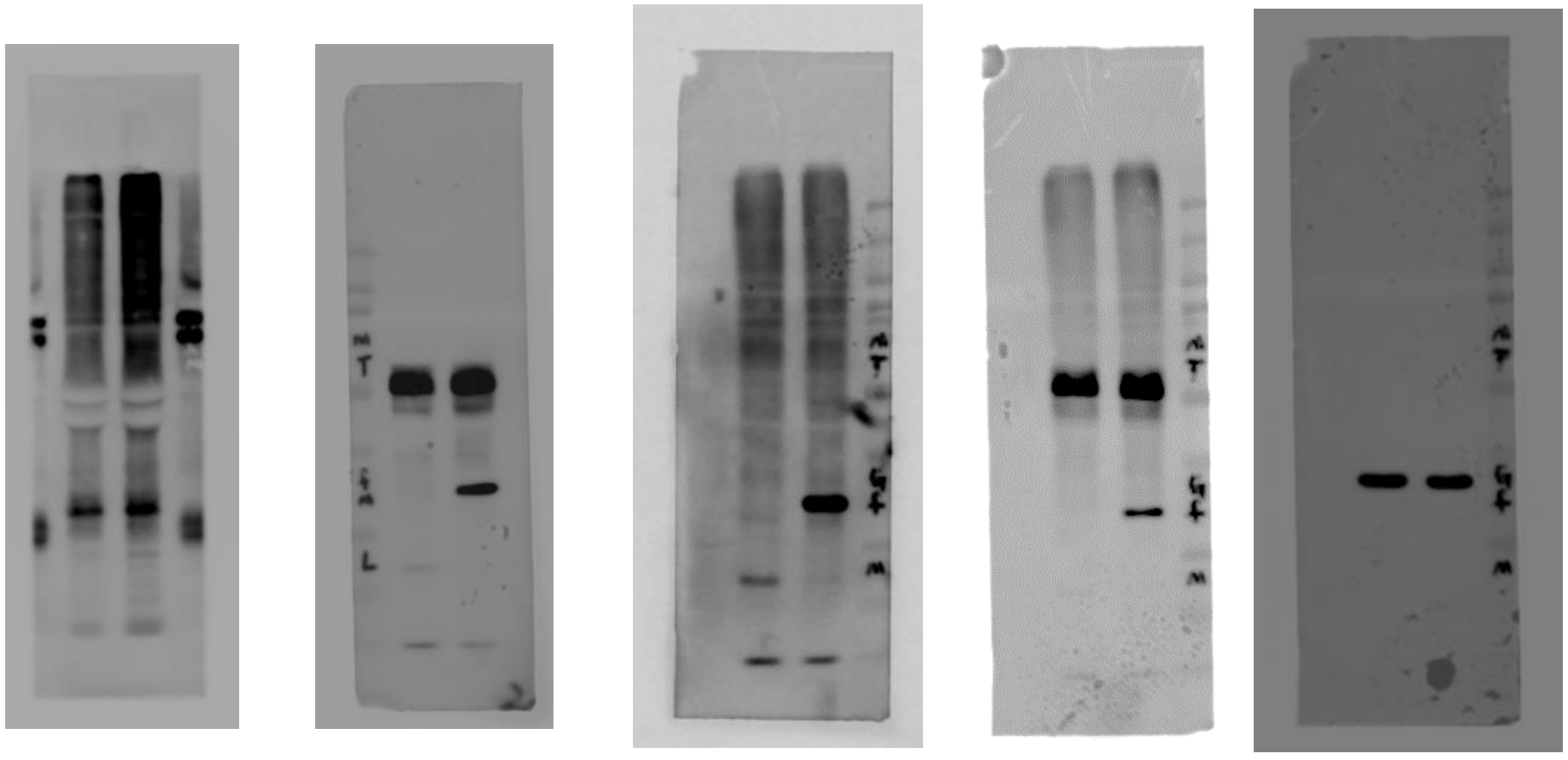

FigS4F-G.

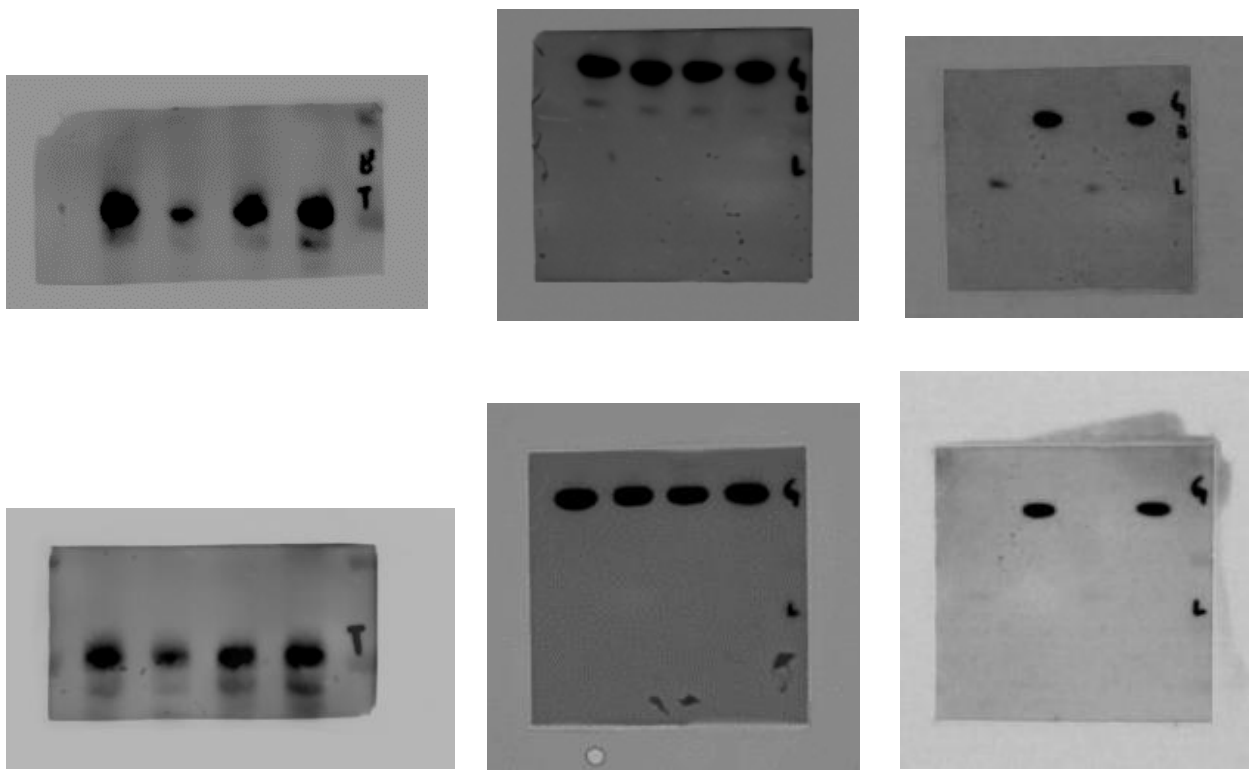

FigS5A.

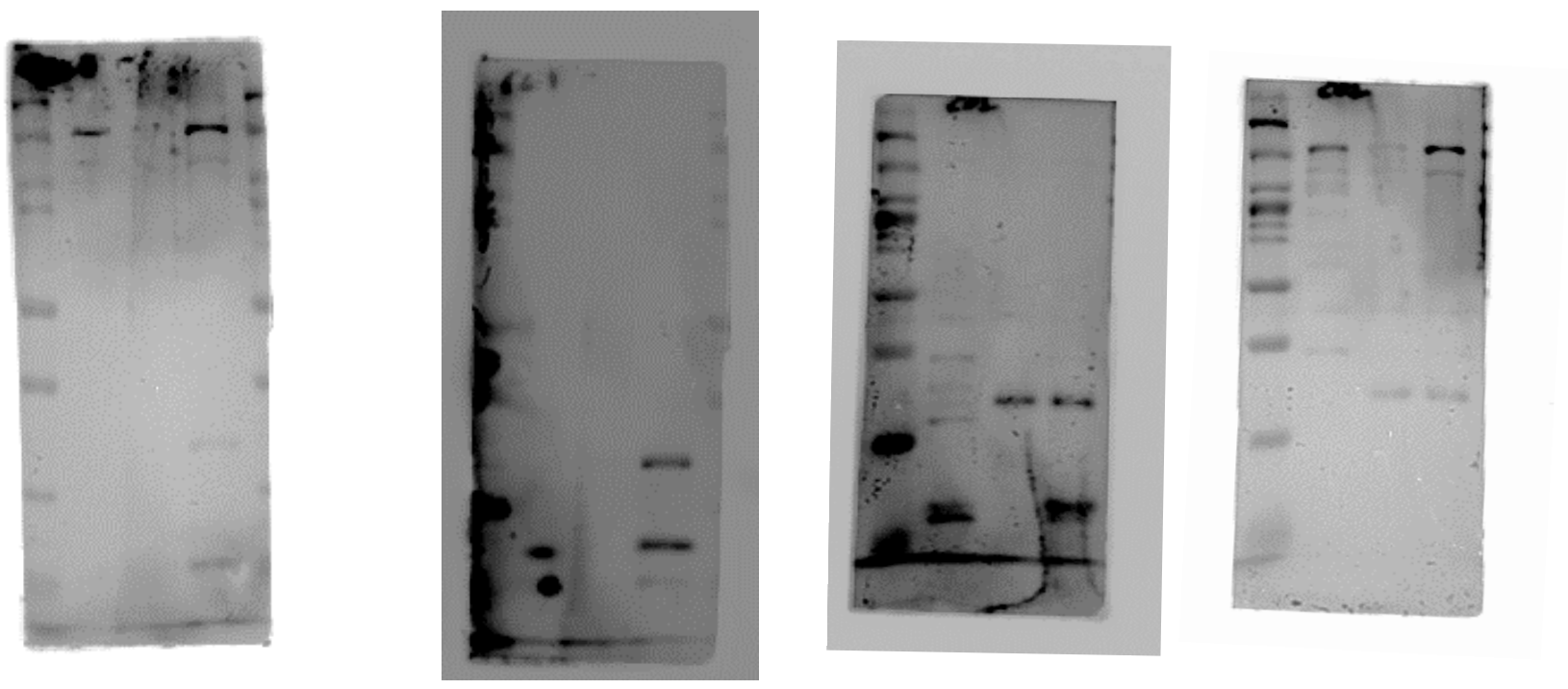

FigS5B.

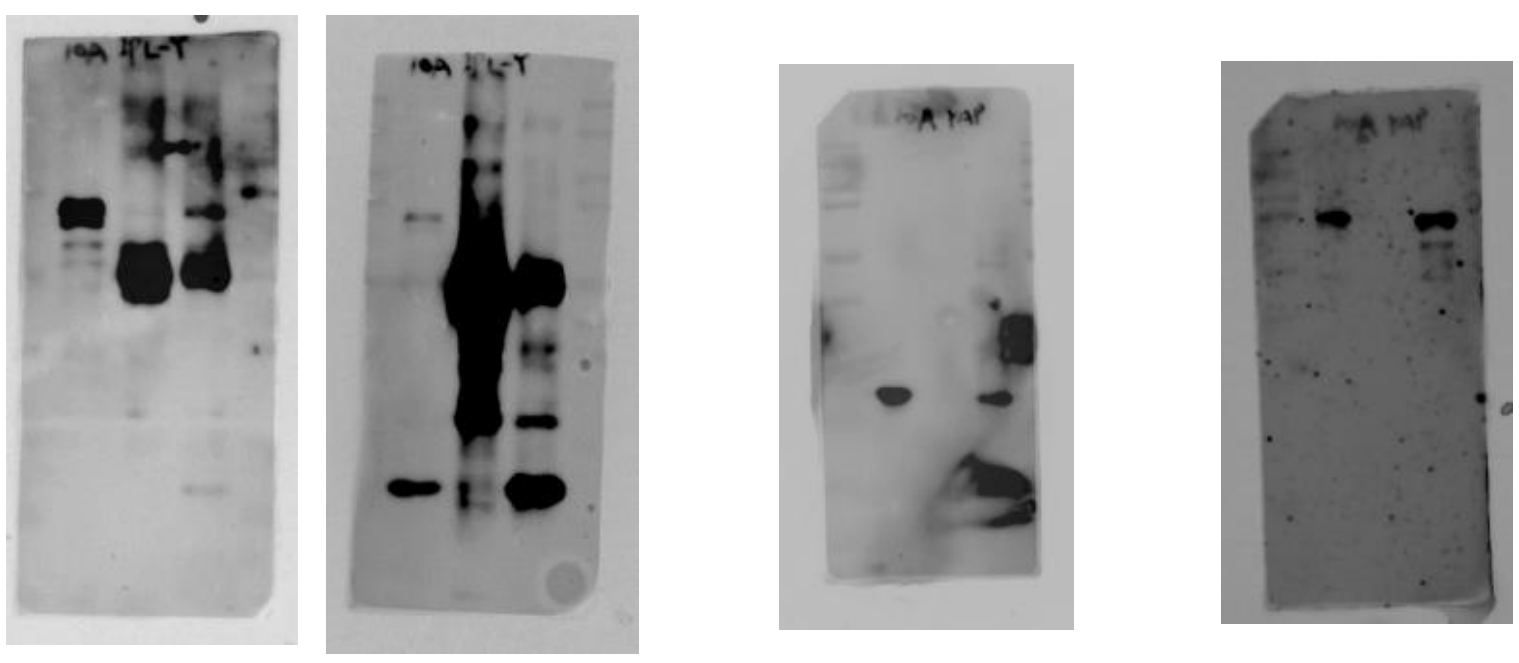

FigS5E.

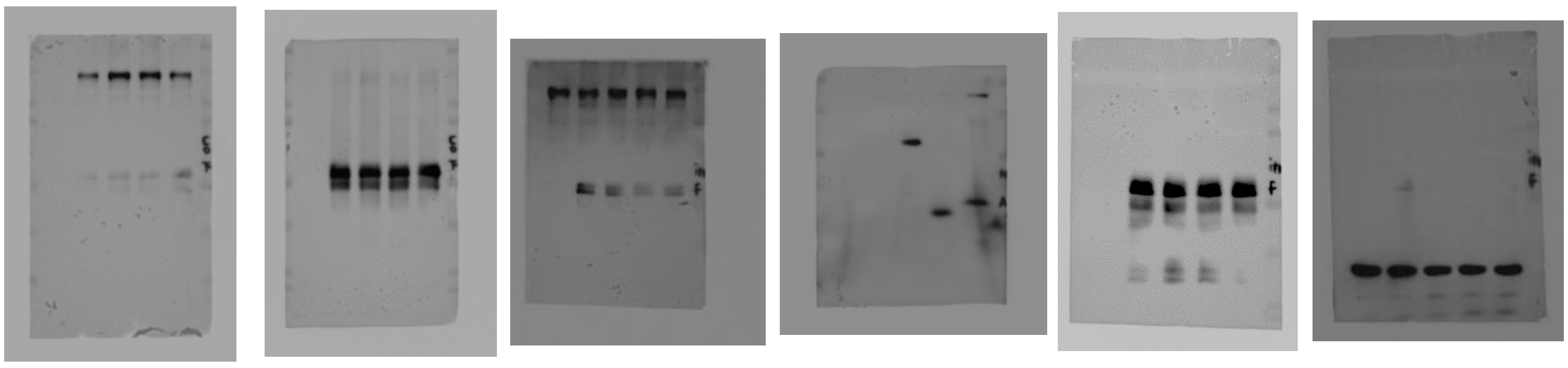

FigS5F.

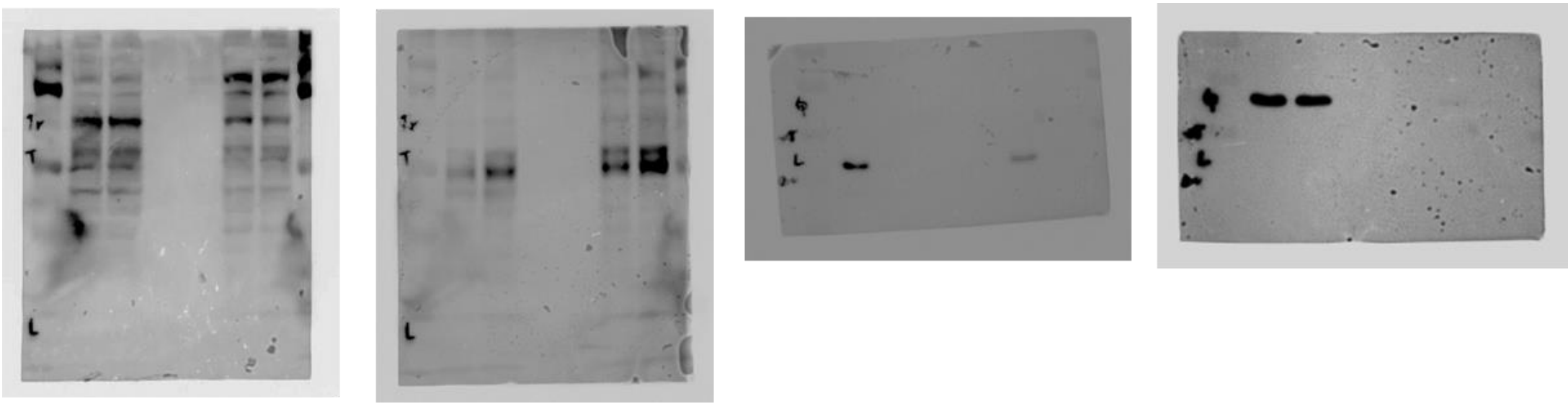

FigS6D-E.

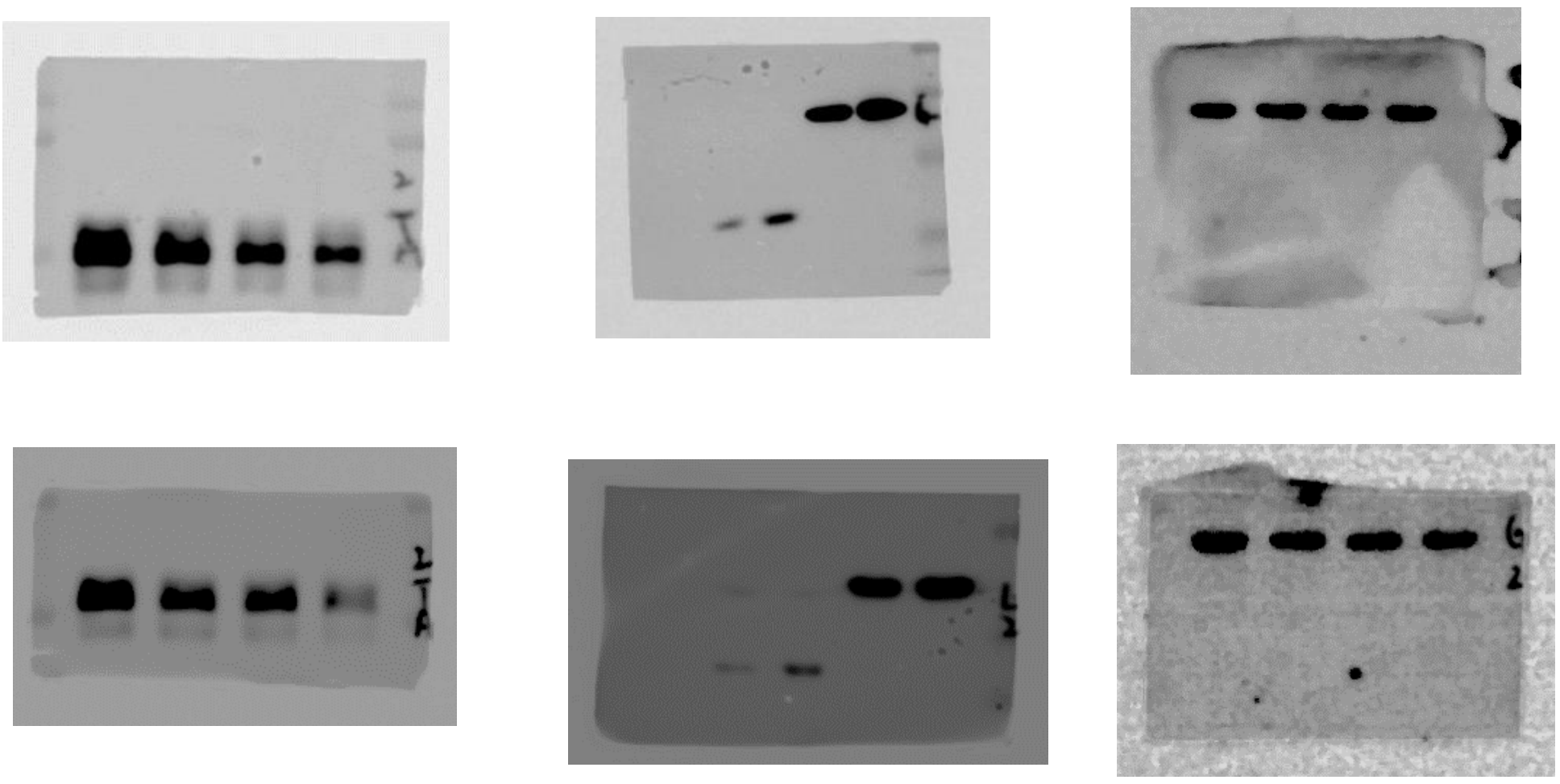

Supplement: Supplementary file 3 [file 41419_2026_9010_MOESM3_ESM.pdf]
